# Effectiveness of remote home monitoring for patients with Chronic Obstructive Pulmonary Disease (COPD): systematic review

**DOI:** 10.1186/s12913-022-07938-y

**Published:** 2022-05-14

**Authors:** Fernanda Inagaki Nagase, Tania Stafinski, Melita Avdagovska, Michael K. Stickland, Evelyn Melita Etruw, Devidas Menon

**Affiliations:** 1 School of Public Health, Health Technology and Policy Unit, University of Alberta, 3-021 Research Transition Facility, Edmonton, AB T6G 2V2 Canada; 2 Alberta Health Services, Edmonton, AB Canada; 3 Division of Pulmonary Medicine, Faculty of Medicine & Dentistry, University of Alberta, Edmonton, AB Canada; 4 G.F. MacDonald Centre for Lung Health, Covenant Health, Edmonton, AB Canada; 5 Faculty of Rehabilitation Medicine, University of Alberta, Edmonton, AB Canada

**Keywords:** COPD, Remote monitoring, Home-based, Systematic review

## Abstract

**Background:**

Although remote home monitoring (RHM) has the capacity to prevent exacerbations in patients with chronic obstructive pulmonary disease (COPD), evidence regarding its effectiveness remains unclear. The objective of this study was to determine the effectiveness of RHM in patients with COPD.

**Methods:**

A systematic review of the scholarly literature published within the last 10 years was conducted using internationally recognized guidelines. Search strategies were applied to several electronic databases and clinical trial registries through March 2020 to identify studies comparing RHM to ‘no remote home monitoring’ (no RHM) or comparing RHM with provider’s feedback to RHM without feedback. To critically appraise the included randomized studies, the Cochrane Collaboration risk of bias tool (ROB) was used. The quality of included non-randomized interventional and comparative observational studies was evaluated using the ACROBAT-NRSI tool from the Cochrane Collaboration. The quality of evidence relating to key outcomes was assessed using Grading of Recommendations, Assessment, Development and Evaluations (GRADE) on the following: health-related quality of life (HRQoL), patient experience and number of exacerbations, number of emergency room (ER) visits, COPD-related hospital admissions, and adherence as the proportion of patients who completed the study. Three independent reviewers assessed methodologic quality and reviewed the studies.

**Results:**

Seventeen randomized controlled trials (RCTs) and two comparative observational studies were included in the review. The primary finding of this systematic review is that a considerable amount of evidence relating to the efficacy/effectiveness of RHM exists, but its quality is low. Although RHM is safe, it does not appear to improve HRQoL (regardless of the type of RHM), lung function or self-efficacy, or to reduce depression, anxiety, or healthcare resource utilization. The inclusion of regular feedback from providers may reduce COPD-related hospital admissions. Though adherence RHM remains unclear, both patient and provider satisfaction were high with the intervention.

**Conclusions:**

Although a considerable amount of evidence to the effectiveness of RHM exists, due to heterogeneity of care settings and the low-quality evidence, they should be interpreted with caution.

**Supplementary Information:**

The online version contains supplementary material available at 10.1186/s12913-022-07938-y.

## Background

Chronic obstructive pulmonary disease (COPD) is a common, preventable lung disease characterized by long-term breathing problems and poor airflow due to airway and alveolar dysfunction commonly caused by smoking [1, 2]. COPD is one of the leading causes of morbidity and mortality worldwide, with a substantial economic and social burden on individuals and society [2, 3]. Patients with COPD often suffer from comorbid diseases including heart failure, diabetes, and depression, making management of these patients complex and multifactorial [4].

Previous studies have shown that acute exacerbation are common in patients with COPD, and increasing frequency of exacerbations is associated with a decrease in lung function [5, 6], and an increase in the use of health services [7]. Integrating remote home monitoring (RHM) into clinical care may support patient self management, and lead to improvements in symptoms and quality of life, while reducing COPD exacerbations burden and healthcare utilization [1, 2, 8–10]. Tomasic et al. have described remote monitoring as encompassing “automatic continuous physiological data transmission and processing decision support, the prediction of deterioration and alarming” [9]. Although, RHM has the capacity to prevent exacerbations, evidence concerning its safety and effectiveness remains unclear. Therefore, the objectives of this study was to determine the effectiveness of RHM programs for patients with COPD. This study was part of a project commissioned by Alberta Health to optimize care of patients with COPD in Alberta, Canada.

## Methods

A systematic review of peer-reviewed primary studies was conducted following the Cochrane Handbook and Preferred Reporting Items for Systematic Reviews and Meta-analysis (PRSMA) guidelines [11, 12].

### Search strategy

An experienced medical information specialist in consultation with the research team iteratively developed a comprehensive, structured search strategy. It was peer-reviewed by another senior information specialist for quality assurance using the Peer Review of Electronic Search Strategies (PRESS) checklist [13]. The search strategy was applied to the following databases: Ovid MEDLINE, Embase, Cochrane Central Register of Controlled Trials, Cochrane Database of Systematic Reviews, Database of Abstracts of Reviews of Effects, Health Technology Assessment and the NHS Economic Evaluation Database. We also searched CINAHL and EconLit on the Ebsco platform and Web of Science. Details of the search strategy are presented in online supplementary Table S1. The search was conducted from March 1^st^ to March 13^th^, 2020. The electronic searches were also supplemented by manual searches of reference lists from included studies. Results from the search strategy were compiled into Reference Manager which was used to manage all references.

### Eligibility criteria

Two reviewers independently screened the titles and abstracts of all citations to identify studies for a full review. Full papers corresponding to potentially relevant citations were retrieved, divided among, and assessed by three reviewers for inclusion/exclusion according to criteria (Table 1). Although RCTs are considered the gold standard in assessing interventions under specific settings, observational studies may provide evidence on the effectiveness of RHM compared to usual care in the “real world”. As this study was commissioned to inform policy decisions, studies were not exluded based on design and quality. Reviewers met to compare results and agree on the final set of studies to include. At both screening steps, consensus between reviewers was assessed using the Kappa statistics and found to be “substantial”.

**Table 1:** Remote Home Monitoring PICOS elements of the clinical effectiveness review protocol

Parameter	Inclusion Criteria	Exclusion Criteria
***P****articipants*	• Patients With COPD	• Patients with Asthma • No patients (simulation studies)
***I****ntervention*	• Remote home monitoring (home is defined as independent or supportive living environments)	• Remote home monitoring programs for patients living in Long Term Care Facilities or Nursing Homes • Remote monitoring that is part of an outpatient program delivered in a hospital or community setting • Remote monitoring that is part of an inpatient program
***C****omparator*	• Usual care (patients managed by their General Practitioner, specialist or both according to local practices)	
***O****utcomes*	• Health-related quality of life • Patient experience • Frequency of exacerbations • Healthcare resource utilization ◦ Hospital admissions ◦ ER visits ◦ Physician visits • Adherence to/ compliance with treatment • Safety • Exercise capacity and activity levels • Mental Health • Self-efficacy • Cost per patient • Provider experience • Lung function and symptoms	• Studies without any defined clinical outcomes • Studies with no relevant clinical outcomes
***S****tudy Design*	Comparative studies • Randomized and non-randomized controlled trials (RCTs and non-RCTs) • Cohort studies • Case–control studies	• Non-English language • Expert reviews • Editorials and opinion pieces • Studies published prior to 2010

### Data extraction and synthesis

Extracted data were tabulated to identify trends or patterns across studies and facilitate qualitative and quantitative comparative analyses. Key characteristics of included studies, their quality, potential sources of bias, and findings were synthesized narratively. A narrative synthesis of effectiveness outcomes across the studies was undertaken. Analysis was based on the types of technologies used for home monitoring which were grouped into three groups: (1) smartphones, apps, tablets; (2) dedicated home monitoring devices; (3) phone calls and text messages. Additionally, studies were assessed to determine whether patient populations, designs, and outcomes were similar enough to perform meta-analyses. Results were pooled if outomes were assessed with the same measures and follow-up times. Heretogeneity was assumed to be too substancial to pool data when the I^2^ statistic was equal to or greater than 50% [11]. Forest plots were used to display individual and pooled results. A p value < 0.05 was considered statistically significant in all analyses. Pooled risk ratios for categorical data and mean differences with 95% confidence intervals (CIs) for continuous outcomes were reported. Publication bias was assessed using funnel plots, where sufficient data (i.e. at least ten studies) were available from the meta-analyses [14]. Multiple studies published with an overlap of outcomes and patients were combined.

### Assessment of study quality

RCTs were appraised using the Cochrane Collaboration Risk of Bias tool (ROB) [15]. The methodological quality of the non-RCT interventional and comparative observational studies were evaluated using the Cochrane Risk of Bias Assessment Tool for Non-Randomized Studies (ACROBAT-NRSI [16]. The quality of evidence relating to key outcomes of interest were assessed using the Grading of Recommendations, Assessment, Development and Evaluations (GRADE) tool [17]. Prior to conducting the systematic review, a small questionnaire was conducted with members of an Expert Advisory Group (EAG) to rank the outcomes according to their importance. The six outcomes with the highest rank were included in the GRADE assessment [11, 18]. The EAG was arranged to oversee the project and involved clinicians, COPD program coordinators, policy makers and researchers. In this review, GRADE assessment was conducted by two independent reviewers and based on the following outcomes: health-related quality of life (HRQoL), patient experience and number of exacerbations, number of emergency room (ER) visits, COPD-related hospital admissions, and adherence as the proportion of patients who completed the study. Discrepancies between the reviewers were resolved through discussion.

## Results

### Search results

**Fig. 1:**
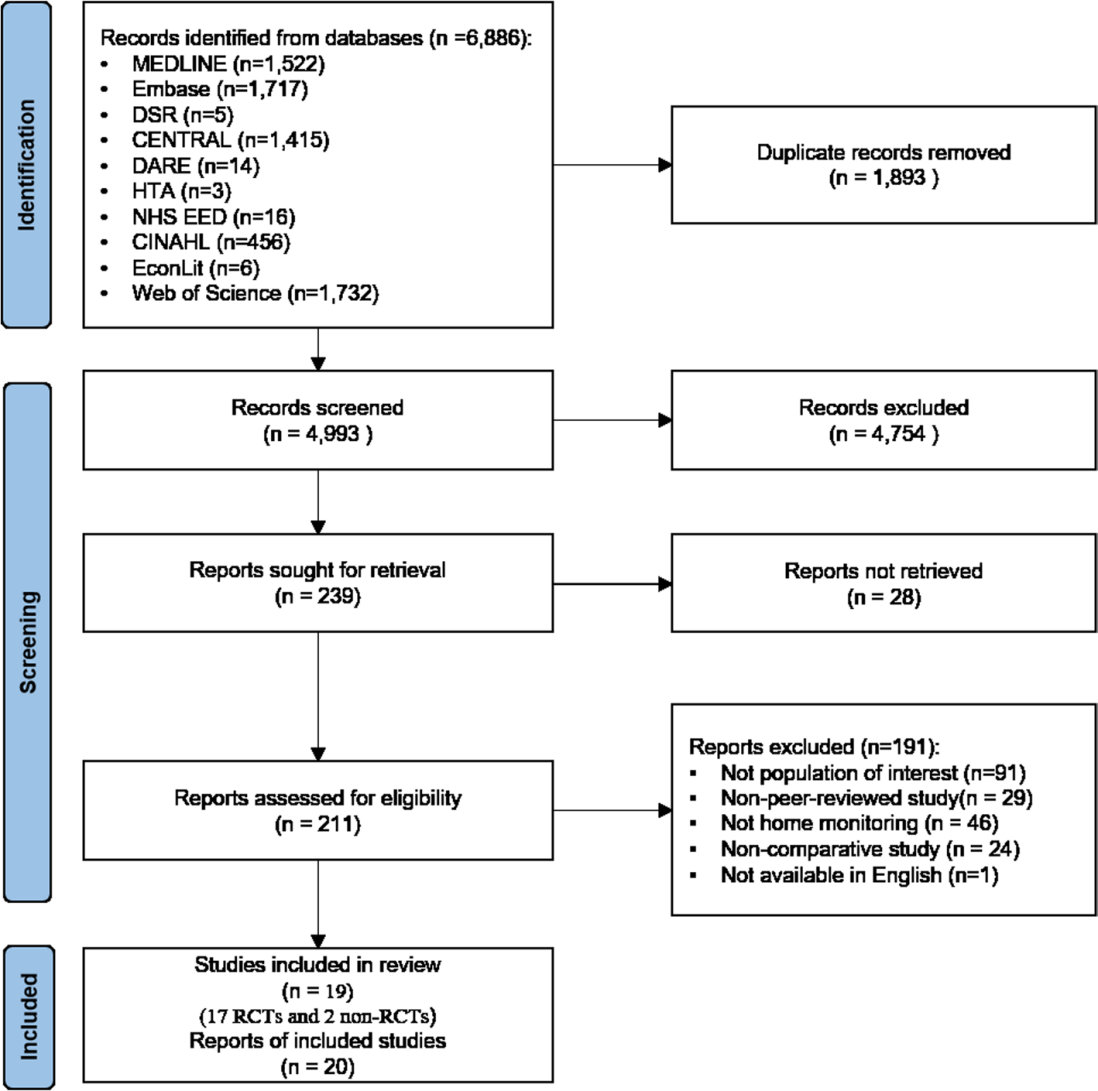
Preferred Reporting Items for Systematic Reviews and Meta-Analyses (PRISMA) diagram of literature search and study selection for efficacy/effectiveness review of remote home monitoring (RHM)

Four thousand nine hundred ninety-three discrete citations were identified through the literature searches and screened, of which 239 were retrieved for full consideration. Twenty papers met the criteria for inclusion in the review. They comprised 17 RCTs and 2 comparative observational studies. Literature search results described using the PRISMA flow diagram are shown in Fig. 1.

### Characteristics of studies

Seventeen [19–35] of the 19 studies [19–38] compared remote home monitoring (RHM) to ‘no remote home monitoring’ (no RHM), and two [36, 37] compared RHM with provider’s feedback to RHM without feedback. The 19 studies were conducted between 2006 and 2018 and had sample sizes ranging from 34 to 1,238 patients (details of participants' characteristics included in the studies are presented in online supplementary Table S3). Collectively, they included 3,144 patients with COPD. Ten studies [19–22, 25, 26, 30, 32, 34, 35] recruited patients from multiple centres, and one [21] spanned five European countries (Spain, the United Kingdom, Slovenia, Estonia and Sweden). The remaining eighteen studies were conducted in Australia (2) [24, 27], Canada (1) [30], Denmark (1) [32], Germany (2) [28, 37], Hong Kong (1) [31], Italy (1) [25], Netherlands (3) [20, 22, 23], South Korea (1) [19], Spain (2) [26, 29], the United Kingdom (1) [33], and the United States (3) [34–36] (Table 2).

**Table 2:** Characteristics of included studies

Study (country)	Study period (Design)	Study objective	Eligibility criteria	Number of centres	Number of participants	Follow-up	Outcomes
***RHM (smartphones, apps, tablets) vs no RHM***							
Park 2020 (South Korea) [19]	Mar 2016- Jun 2018 (RCT)	To examine the effect of a smartphone app-based, self-management program on self-care behavior	*Inclusion criteria:* • Age ≥ 45 years old • Mild, moderate or severe COPD • Had a smartphone and could text messages • Able to communicate *Exclusion criteria:* • Psychiatric disorder • COPD-related hospitalization in the last 2 months • Exacerbation • Oxygen saturation < 93% in a stable state or < 85% after a six minute walk test • Severe respiratory symptoms in a stable state • Attended PR in the previous year • Other diseases that made physical activity and/or exercise difficult • Use of assistive devices to walk or problems with balance	Multiple centres	RHM: 23 no RHM: 21	6 months	• Adherence • ER visits • Exacerbations • Exercise capacity and activity levels • Health-related quality of life • Hospital admissions • Lung function and other symptoms • Mental health • Patient experience • Safety • Self-efficacy • Visits to physician
Boer 2019 (Netherlands) [20]	Jun 2015- Jul 2016 (RCT)	To examine the effects of a smart mobile health (mHealth) tool that supports COPD patients in the self-management of exacerbations	*Inclusion criteria:* • Age ≥ 40 years old • Spirometry-confirmed diagnosis of COPD (FEV_1_/FEVC < 70%) • 2 or more exacerbations in the last year • Had experienced 2 or more symptom-based exacerbations *Exclusion criteria:* • Severe comorbid conditions that prohibited safe participation • Insufficient knowledge of the Dutch language • Persisting difficulties in using the mHealth system after a 2-week practice period and additional assistance	Multiple centres	RHM: 43 no RHM: 44	12 months	• Adherence • Exacerbations • Health-related quality of life • Hospital admissions • Lung function and other symptoms • Mortality • Patient experience • Self-efficacy • Visits to physician
Walker 2018 (Spain, UK, Slovenia, Estonia, Sweden) [21]	Oct 2013- Apr 2016 (RCT)	To evaluate the effectiveness of remote monitoring in reducing healthcare utilization	*Inclusion criteria:* • Age ≥ 60 years old • Moderate to very severe diagnosis of COPD • Acute exacerbation with or without hospitalization in the previous year • Smoking history of ≥ 10 pack-years • One or more chronic conditions (congestive heart failure, ischemic heart disease, hypertension, hyperlipidemia and clinically significant sleep-disordered breathing) • Clinically stable, with at least 4 weeks since the last exacerbation *Exclusion criteria:* • Significant visual disturbance or mental health disorders • Planned prolonged absence from home • Living in areas not covered by a mobile data network • Unable to use the study equipment	Multiple centres	RHM: 154 no RHM: 158	9 months	• Adherence • Cost • Exacerbations • Health-related quality of life • Hospital admissions • Length of hospitalization • Mental health • Mortality
Tabak 2014a (Netherlands) [22]	Dec 2011- Jul 2013 (RCT)	To investigate the satisfaction and use of telehealth in patients with COPD	*Inclusion criteria:* • Age > 40 years old • Diagnosis of COPD based on the GOLD criteria • Internet access at home • Able to understand Dutch *Exclusion criteria:* • Age < 75 years old • Exacerbation in the previous month • Three or more exacerbations in the previous two years • One hospitalization for respiratory problems in the previous two years • Serious disease with low survival rates • Other diseases influencing bronchial symptoms and/or lung function (e.g., cardiac insufficiency, sarcoidosis) • Severe psychiatric illness • Uncontrolled diabetes mellitus	Multiple centres	RHM: 15 no RHM: 14	9 months	• Adherence • ER visits • Exacerbations • Exercise capacity and activity levels • Health-related quality of life • Hospital admissions • Length of hospitalization • Lung function and other symptoms • Patient experience
Tabak 2014b (Netherlands) [23]	Oct 2010- Apr 2011 (RCT)	To assess the effectiveness of telerehabilitation in patients with COPD	*Inclusion criteria:* • Current or former smoker • Able to read and speak Dutch • Internet access at home *Exclusion criteria:* • Infection or exacerbation in the previous month • Impaired hand function causing inability to use the intervention • Disorders or progressive disease seriously influencing daily activities (e.g. amputation) • Other diseases influencing bronchial symptoms and/or lung function (e.g. sarcoidosis) • Regular oxygen therapy (> 16 h per day or pO2 < 7.2 kPa) • Asthma • Attended physiotherapy in the last six weeks	NR	RHM: 18 no RHM: 16	1 month	• Adherence • Exercise capacity and activity levels • Health-related quality of life • Lung function and other symptoms
***RHM (dedicated monitoring devices) vs no RHM***							
Shany 2017 (Australia) [24]	Mar 2009- Oct 2010 (RCT)	To investigate the effects of home tele monitoring in patients with severe COPD	*Inclusion criteria:* • At least one hospital admission for an exacerbation in the last year *Exclusion criteria:* • Insufficient English fluency • Motor deficits that might prevent the use of the telehealth • Cognitive impairment • Participation in another trial • No landline phone connection at home	Single centre	RHM: 21 no RHM: 21	12 months	• Adherence • Cost • ER visits • Hospital admissions • Length of hospitalization • Mental health • Mortality • Patient experience • Provider experience
Vianello 2016 (Italy) [25]	Nov 2011- May 2014 (RCT)	To investigate the benefits of a telemonitoring system in managing acute exacerbation advanced-stage COPD patients	*Inclusion criteria:* • Age ≥ 18 years old • Severe to very severe diagnosis of COPD • Life expectancy > 12 months • Capability of using, alone or assisted, the intervention *Exclusion criteria:* • Concomitant significant lung disease • Negative advice of the GP • Serious social problems, including lack of adequate family support and/or other social support networks	Multiple centres	RHM: 230 no RHM: 104	12 months	• Adherence • ER visits • Health-related quality of life • Hospital admissions • Length of hospitalization • Mental health • Mortality • Visits to physician
Segrelles 2014 (Spain) [26]	Jan 2010- Jul 2011 (RCT)	To assess the efficacy and effectiveness of a home telehealth program for COPD patients with severe airflow obstruction	*Inclusion criteria:* • Age ≥ 50 years old • Severe to very severe diagnosis of COPD (FEV_1_ < 50% predicted, FEV_1_/FVC ratio < 70%) • Long-term home oxygen therapy *Exclusion criteria:* • Current smoker • Enrolled in a palliative care program • Institutionalized or at risk of social exclusion • Unable to understand all procedures	Multiple centres	RHM: 30 no RHM: 30	7 months	• Adherence • ER visits • Hospital admissions • Length of hospitalization • Mortality
De San Miguel 2013 (Australia) [27]	NR (RCT)	To understand the impact of telehealth monitoring for COPD patients on health service utilization and cost-effectiveness	*Inclusion criteria:* • Diagnosis of COPD • Use of home oxygen therapy • Able to speak English *Exclusion criteria:* • Dementia • Palliative care • No telephone landline • Unable to use telehealth equipment due to cognitive impairment or physical disability	Single centre	RHM: 40 no RHM: 40	6 months	• Cost • ER visits • Health-related quality of life • Hospital admission • Length of hospitalization • Patient experience • Visits to physician
Jehn 2013 (Germany) [28]	Jan 2012- Jan 2013 (RCT)	To determine if the use of home monitoring reduces risk of exacerbations due to changes in the weather	*Inclusion criteria:* • Age ≥ 40 years old • Moderate to very severe diagnosis of COPD (FEV_1_ < 80% predicted and FEV_1_/FVC ratio < 70%) • At least one exacerbation in the previous year • Clinically stable for the last month *Exclusion criteria:* • Asthma • Long-term oxygen therapy • Severe heart, liver or kidney disease • Any end stage malignant disease with life expectancy of less than six months • Listed for a lung transplant • Severe depression • Residents in nursing home • Physical disabilities limiting them from performing six minute walk tests • Mentally disabled	Single centre	RHM: 32 no RHM: 30	9 months	• Adherence • Exacerbations • Exercise capacity and activity levels • Health-related quality of life • Hospital admissions • Length of hospitalization • Lung function and other symptoms • Visits to physician
Jodar-Sanchez 2013 (Spain) [29]	Sep 2010- May 2011 (RCT)	To analyze the effectiveness of a telehealth programme in patients with advanced COPD	Inclusion criteria: • Adult • Diagnosis of COPD • Long-term oxygen therapy • At least one hospitalisation for respiratory illness in the previous year • Clinically stable during the previous three months *Exclusion criteria:* • No home telephone line	Single centre	RHM: 24 no RHM: 21	4 months	• Adherence • ER visits • Health-related quality of life • Hospital admissions • Length of hospitalization • Mortality • Patient experience • Provider experience • Visits to physician
Pare 2013 (Canada) [30]	Sep 2010- Oct 2011 (RCT)	To assess the effectiveness of home monitoring in reducing costs associated with managing COPD	*Inclusion criteria:* • Very serious COPD requiring frequent home visits (FEV_1_ < 45% predicted) • At least one hospitalization in the previous year • Willingness to manage their health status (with or without an informal caregiver) • Able to communicate in English or French • An operational telephone line at the home *Exclusion criteria:* • Suffered from psychological or psychiatric problems • Cognitive deficit • Visual or motor deficit that would unable the use of the intervention unless an informal caregiver agreed to assist	Multiple centres	RHM: 60 no RHM: 60	Pre-phase: 12 months Post phase: 12 months	• Cost • ER visits • Hospital admissions • Length of hospitalization • Patient experience
Chau 2012 (Hong Kong) [31]	2010- NR (RCT)	To examine user satisfaction and effectiveness of telecare services in patients with COPD	*Inclusion criteria:* • Age ≥ 60 years old • Moderate or severe COPD • At least one hospital admission due to exacerbation in the previous year *Exclusion criteria:* • Impaired cognitive function • Illiterate • Hearing problems • Unable to operate the telecare device	Single centre	RHM: 30 no RHM: 23	Mean RHM: 65.18 days no RHM: 68.44 days	• Adherence • ER visits • Health-related quality of life • Hospital admissions • Length of hospitalization • Lung function and other symptoms • Patient experience
Dinesen 2012 (Denmark) [32]	NR (RCT)	To test whether preventive home monitoring in COPD reduced the admission rate to hospital and the cost of hospitalization	*Inclusion criteria:* • Age > 18 years old • Diagnosis of severe or very severe COPD • Able to understand oral and written information *Exclusion criteria:* • Heart disease that could limit physical activity • Mental illness • Terminal malignancy disease • Severe rheumatoid arthritis • Pregnancy	Multiple centres	RHM: 60 no RHM: 51	10 months	• Adherence • Cost • Hospital admissions
Lewis 2010 (UK) [33]	Nov 2007 – Mar 2009 (RCT)	To determine if telemonitoring in stable, and optimized COPD patients affects their health care utilization	*Inclusion criteria:* • Diagnosis of moderate to severe COPD • Completed at least 12 out of 18 sessions of outpatient PR • Have a GP • Have a standard telephone line *Exclusion criteria:* • Chronic asthma and interstitial lung disease • Unstable cardiac disease • Cognitive impairments • Other medical conditions that would unable the use of the intervention • Living in nursing or residential institution • Participation in any investigational drug trial in the last month • Mental condition rendering the patient unable to understand the nature, scope and possible consequences of the study	Single centre	RHM: 20 no RHM: 20	12 months	• Adherence • ER visits • Health-related quality of life • Hospital admissions • Length of hospitalization • Mental health • Mortality • Patient experience • Visit to physician
Au 2015 (USA) [34]	2006- 2007 (Observational)	To examine the effects of telemonitoring on resource use among Medicare patients with COPD	*Inclusion criteria:* • At least a diagnosis of COPD, congestive heart failure, or diabetes mellitus Exclusion criteria: • Comorbidities such as dementia or blindness that would limit interaction with the program	Multiple centre	RHM: 619 no RHM: 619	3 years	• Adherence • ER visits • Exacerbations • Hospital admissions • Length of hospitalization
Davis 2015 (USA) [35]	Oct 2010- Aug 2012 (Retrospective study)	To determine feasibility of a transitional care program that integrated mobile health technology and home visits for underserved COPD and HF patients	*Inclusion criteria:* • Diagnosis of COPD or HF • Underserved • Able to speak English or Spanish • US residence • Independent in their own care or with reliable caregiver *Exclusion criteria:* • End-stage COPD or HF • Hospice candidate • Cancer • Pulmonary fibrosis • On dialysis • Discharged to a setting other than home	Multiple centres	RHM: 58 no RHM: 174	3 months	• Adherence • ER visits • Health-related quality of life • Mortality • Patient experience
***RHM with feedback (phone calls, text messages) vs RHM with no feedback***							
Sink 2018 [39] (USA)	Jan 2016- Dec 2016 (RCT)	To study the effect of an automated telemedicine intervention on patients’ time-to-hospitalization	*Inclusion criteria:* • Diagnosis of COPD • Age > 18 years old • Willingness to provide a telephone number at which they can receive text messages or voice phone messages *Exclusion criteria:* • Intention to transfer care away from the clinic	Single centre	RHM: 83 no RHM: 85	8 months	• Adherence • Hospital admissions
Franke 2016 (Germany) [37]	Sep 2012- Mar 2015 (RCT)	The primary aim was to compare daily exercise times in patients with stable COPD, either with or without supporting phone calls	*Inclusion criteria:* • Moderate to very severe diagnosis of COPD *Exclusion criteria:* • Malignancy • Symptomatic cardiac disease	Single centre	Total: 53^a^	6 months	• Adherence • Exercise capacity and activity levels • Health-related quality of life

*Notes*: Tabak 2014a [22] and Tabak 2014b [23] used the same exercise monitoring device and smartphone technology. De San Miguel 2013  [27] and Lewis 2010 [33] used the same telemonitoring device. Segrelles 2014 [26] and Jodar-Sanchez 2013 [29] used the same devices to collect vital signs measures and modem technology to transmit collected measurements

^a^ Cross-over randomized trial

*COPD* Chronic obstructive pulmonary disease, *FEV*_*1*_ Forced expiratory volume in one second, *FVC* Forced vital capacity, *GP* General practitioner, *HF* Heart failure, *PR* Pulmonary rehabilitation, *RCT* Randomized controlled trial, *RHM* Remote home monitoring

### RHM program characteristics

The length of the monitoring period varied from one to 12 months (comparison of what was monitored remotely and when in the included studies is presented in Table 3). At the beginning of the program, nurses taught patients how to use the technology, typically in patients’ homes. However, two studies [19, 20] held group sessions at outpatient clinics and two studies [22, 23] trained patients in their homes and outpatient clinics. In four studies [19, 20, 23, 24], patients also participated in outpatient group education and exercise sessions (a detailed description of the home monitoring program, technology and its components is presented in online supplementary Tables S4, S5, and S6).

**Table 3:** Comparison of what was monitored remotely and when in the included studies

Study	Frequency of monitoring	Symptoms	Medication use	Oxygen saturation	Respiratory rate	Spirometry	Heart rate	Blood pressure	Temperature	Weight	Exercise
*RHM (smartphones, apps, tablets) vs no RHM*											
Park 2020* [19]	• At least 4 times per week and when experiencing an exacerbation • Exercises were only recorded on days patients exercised	✓	✓								✓
Boer 2019 [20]	• Every time patient experienced a change in disease symptoms or burden	✓	✓	✓		✓			✓		
Walker 2018 [21]	• Daily	✓		✓		✓	✓	✓	✓		
Tabak 2014a [22]	• Daily	✓	✓								✓
Tabak 2014b [23]	• Daily	✓	✓								✓
*RHM (dedicated monitoring devices) vs no RHM*											
Shany 2017* [24]	• Daily	✓	✓	✓		✓	✓	✓	✓	✓	
Vianello 2016 [25]	• Every other day and in the event of clinical worsening • Symptoms and medication use were reported only when requested by healthcare provider	✓	✓	✓			✓				
Segrelles 2014 [26]	• Daily			✓		✓	✓	✓			
De San Miguel 2013 [27]	• Daily	✓		✓			✓	✓	✓	✓	
Jehn 2013 [28]	• Daily in the morning during a specified 2 h window • Exercise was monitored weekly in the morning during a specified two-hour window	✓				✓					✓
Jodar-Sanchez 2013 [29]	• Daily • Pulmonary function was measured twice a week			✓		✓	✓	✓			
Pare 2013 [30]	• Daily	✓	✓								
Chau 2012 [31]	• Daily (three times a day)			✓	✓		✓				
Dinesen 2012 [32]	• As prescribed by physician	✓		✓		✓	✓	✓		✓	✓
Lewis 2010 [33]	• Daily (twice a day at specified times)	✓		✓			✓		✓		
Au 2015 [34]	• Daily	✓					✓	✓	✓	✓	
Davis 2015 [35]	• Daily	✓		✓			✓	✓		✓	
*RHM with feedback (phone calls, text messages) vs RHM with no feedback*											
Sink 2018 [39]	• Daily • Frequency reduced to twice a week if no worsening of symptoms for 30 consecutive days • Frequency temporarily resumed to daily if breathing assessment detected a worsening in breathing	✓									
Franke 2016 [37]	• Daily										✓

*Cross-over randomized trial

Comparator interventions in the studies were ‘no RHM’, or RHM without feedback and alerts from healthcare providers. ‘No RHM’ generally comprised usual care based on local practices, in which patients were instructed to contact their healthcare provider if they experienced worsening of symptoms. Five studies [19, 20, 24, 27, 32] reported that patients in the ‘no RHM’ group received education and exercise training sessions or materials similar to those received by the RHM group. RHM without feedback and alerts consisted of patients measuring parameters and transmitting data, but with no healthcare providers' feedback.

The RHM technology and integrated peripheral devices varied across studies; most of the interventions had a dedicated device for home monitoring. Four studies [19, 20, 22, 23] used smartphones and apps as the main device, and in one study [19], education material and exercise training were also available through the app. One study [21] used a tablet to facilitate recording and transmission of data. Two studies [36, 37] used telephone and text messages to monitor patients. Included integrated peripheral devices were: pulse oximeter (in 11 studies) [20, 21, 24–27, 29, 31–33, 35], blood pressure cuff (7) [21, 24, 26, 27, 29, 32, 35], spirometer (6) [20, 24, 26, 28, 29, 32], thermometer (5) [20, 21, 24, 27, 33], heart rate monitor (4) [21, 24, 26, 29], weight scale (4) [24, 27, 32, 35], accelerometer (3) [22, 23, 28], pedometer (2) [19, 32], glucometer (1) [24], peak flow meter (1) [26], and respiration sensor (1) [31]. In the majority of studies, patients were asked to collect data daily. The most common parameters monitored were symptoms (15), oxygen saturation (11) [20, 21, 24–27, 29, 31–33, 35], blood pressure (8) [21, 24, 26, 27, 29, 32, 34, 38], and lung function (7) [20, 21, 24, 26, 28, 29, 32]. All studies reported that transmission of data occurred immediately. In ten studies [19, 24, 27, 28, 30–34, 37] healthcare providers and/or nurses were responsible for monitoring data. Four studies [25, 26, 29, 35] used a central management unit to monitor data and five [20–23, 36] used algorithms and decision trees to monitor and detect changes in symptoms and clinical parameters. In most studies, healthcare providers or nurses contacted patients if clinical worsening was observed and/or data were not recorded for several days. None of the studies discussed data privacy.

### Risk of bias

#### Results of risk of bias assessment

Quality assessment was conducted for the seventeen [19–30, 32, 33, 36–38] RCTs and two non-randomized studies [34, 35] (detailed description in online supplementary Table S7).

##### RCTs

**Fig. 2:**
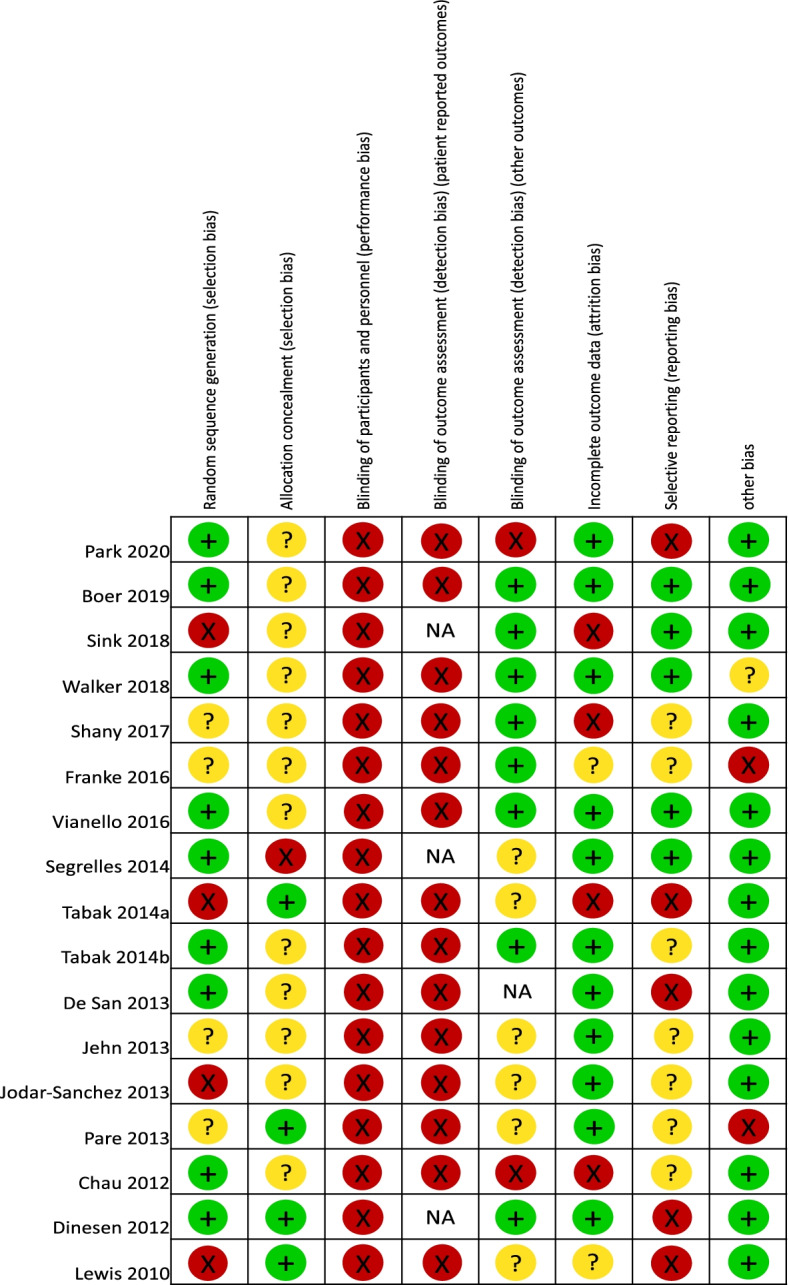
Cochrane risk of bias summary

**Fig. 3:**
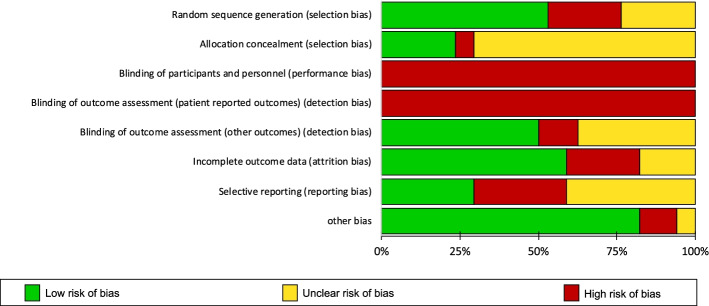
Risk of bias graph with each risk of bias presented as a percentage across all included RCTs

Six studies [19–21, 23, 25, 27] assigned patients to treatment groups via a computer-generated sequence and three [31, 32, 40] used drawing of lots. Four trials [23, 29, 33, 36, 38] were likely at high risk of selection bias since three [23, 29, 33, 38] reported an unequal distribution of patients’ characteristics between groups, and one [36] had assigned seventeen patients to the control group without randomization. Only five [23, 26, 30, 32, 33, 38] provided details around allocation concealment. Four [23, 30, 32, 33, 38] adhered to adequate methods for keeping patients and investigators unaware of treatment allocation prior to assignment. One study [26] randomized the clinics, rather than patients themselves. Due to the nature of remote monitoring, neither patient nor staff were blinded to the intervention. Thus, the risk of performance bias was high. Patient reported outcomes were also at high risk of observer bias because patients were the assessors and not blinded to the type of intervention they had received. Eight studies [20–22, 24, 25, 32, 36, 37] were at low risk of observer bias. Six RCTs [22, 26, 28–30, 33, 38] provided insufficient information to determine the presence of observer bias. Four RCTs [23, 24, 31, 36] were at high risk of attrition bias. Three studies reported differences in the frequency of missing data and reasons for dropouts between groups. The risk of attrition bias was low in eleven of the RCTs [19–22, 25–30, 32], where the extent of missing data was small and similar between groups. Two studies [33, 37, 38] did not provide sufficient information to determine the risk of attrition bias. Ten trials [19, 22–24, 27–32] did not publish or register their protocols and five [19, 32, 33, 37, 38] were considered to have an incomplete follow-up data on outcome measures described in trial registrations and study methods sections (Figs. 2 and 3).

##### Non-randomized studies

**Fig. 4:**
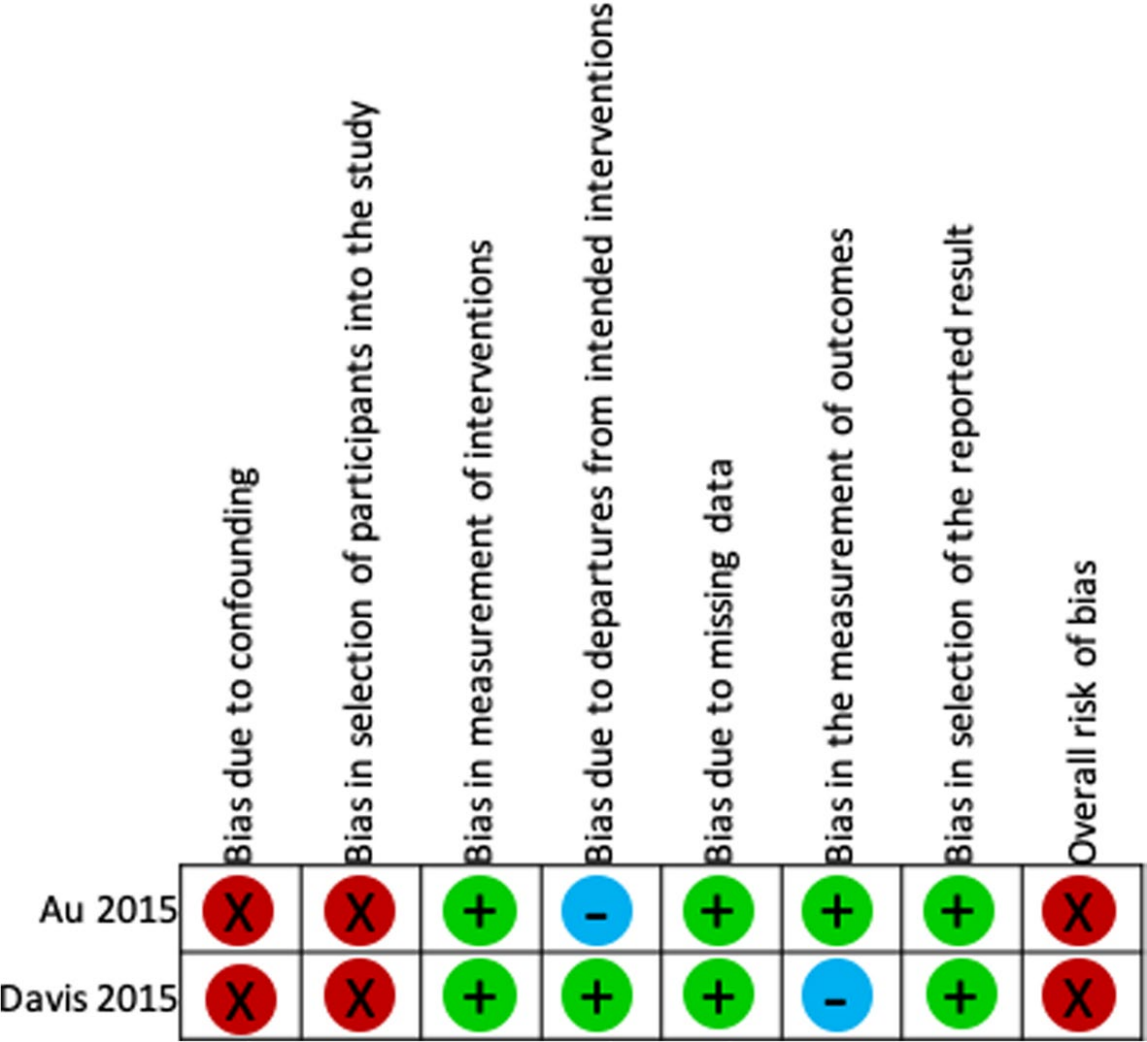
ACROBAT-NRSI summary

**Fig. 5:**
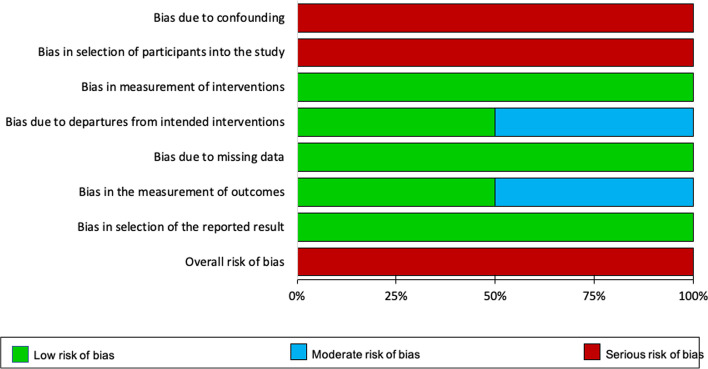
ACROBAT-NRSI graph with each risk of bias item presented as a percentage across all included non-randomized studies

Both non-randomized studies were at serious risk of bias due to confounding and patient selection. They used methods to adjust for socioeconomic variables, but did not measure and adjust for clinical confounders (e.g. FEV_1_, severity of COPD). Further, recruitment into these studies was based on availability of the technology and patients’ preferences [34, 35]. One non-randomized study [34] measured objective outcomes unlikely to be influenced by knowledge of the intervention received. The second non-randomized study [35] did not blind the outcome assessor to intervention type. However, in both of these studies, the intervention status remained the same throughout their duration, minimizing the risk of bias in the measurement of interventions. In the two non-randomized studies [34, 35], data were reasonably complete. None of the non-randomized studies [34, 35] discussed the care received by the comparator group (Figs. 4 and 5).

#### Results from GRADE assessment

GRADE assessment was conducted on the selected outcomes (Tables 4, 5 and 6). The GRADE level or certainty of the evidence for these outcomes was very low to low for all outcomes in studies comparing RHM (smartphones, apps, tablets) to no RHM, RHM (dedicated monitoring devices) to no RHM, and RHM (phone calls, text messages) to no RHM.

**Table 4:** Studies comparing remote home monitoring (smartphones, apps, and tablets) to no remote home monitoring

Studies comparing RHM (smartphones, apps, tablets) to no RHM					
**Outcomes**	**№ of participants** **(studies)**	**Certainty of the evidence** **(GRADE)**	Relative effect* (95% CI)	**Anticipated absolute effects**	
				**Risk with no RHM**	**Risk difference with RHM (smartphones, apps, tablets)**
COPD Assessment Test (CAT) scores at the end of monitoring period	312 (1 RCT)	⨁⨁◯◯ LOW ^a,b^	not pooled	not pooled	not pooled
Patient experience- Overall satisfaction at the end of monitoring period	73 (2 RCTs)	⨁◯◯◯ VERY LOW ^a,b,c^	not pooled	not pooled	not pooled
Average number of exacerbations	87 (1 RCT)	⨁⨁◯◯ LOW ^a,b^	not pooled	not pooled	not pooled
Average number of ER visits due to COPD	29 (1 RCT)	⨁⨁◯◯ LOW ^a,b^	not pooled	not pooled	not pooled
Average number of hospital admissions due to COPD	116 (2 RCTs)	⨁⨁◯◯ LOW ^a,b^	not pooled	not pooled	not pooled
Adherence as the proportion of participants who completed the study	506 (5 RCTs)	⨁⨁◯◯ LOW ^a,d^	not pooled	not pooled	not pooled

^*^The risk in the intervention group (and its 95% confidence interval) is based on the assumed risk in the comparison group and the relative effect of the intervention (and its 95% CI)

*CI* Confidence interval, *COPD* Chronic obstructive pulmonary disease, *ER* Emergency room, *RCT* Randomized clinical trial, *RHM* Remote home monitoring

^a^ Study(ies) at high risk of bias

^b^ Small sample size

^c^ Differences in point estimates

^d^ The outcome is an indirect measure of compliance with intervention

**Table 5:** Studies comparing remote home monitoring (dedicated monitoring devices) to no remote home monitoring

Studies comparing RHM (dedicated monitoring devices) to no RHM					
**Outcomes**	**№ of participants** **(studies)**	**Certainty of the evidence** **(GRADE)**	Relative effect* (95% CI)	**Anticipated absolute effects**	
				**Risk with no RHM**	**Risk difference with RHM (dedicated monitoring devices)**
COPD Assessment Test (CAT) scores at the end of monitoring period	62 (1 RCT)	⨁⨁◯◯ LOW ^a,b^	not pooled	not pooled	not pooled
Patient experience- Overall satisfaction at the end of monitoring period	111 (3 RCTs)	⨁⨁◯◯ LOW ^a,b^	not pooled	not pooled	not pooled
Patient experience- Overall satisfaction at the end of monitoring period	69 (1 observational study)	⨁◯◯◯ VERY LOW ^b,c^	not pooled	not pooled	not pooled
Average number of exacerbations	62 (1 RCT)	⨁⨁◯◯ LOW ^a,b^	not pooled	not pooled	not pooled
Average number of exacerbations	1238 (1 observational study)	⨁◯◯◯ VERY LOW ^c^	not pooled	not pooled	not pooled
Average number of ER visits due to COPD	302 (4 RCTs)	⨁⨁◯◯ LOW ^a,b^	not pooled	not pooled	not pooled
Average number of hospital admissions due to COPD	353 (5 RCTs)	⨁⨁◯◯ LOW ^a,b^	not pooled	not pooled	not pooled
Average number of hospital admissions due to COPD	1238 (1 observational study)	⨁◯◯◯ VERY LOW ^c^	not pooled	not pooled	not pooled
Adherence as the proportion of participants who completed the study	707 (7 RCTs)	⨁⨁◯◯ LOW ^a,d^	not pooled	not pooled	not pooled

^*^The risk in the intervention group (and its 95% confidence interval) is based on the assumed risk in the comparison group and the relative effect of the intervention (and its 95% CI)

*CI* Confidence interval, *COPD* Chronic obstructive pulmonary disease, *ER* Emergency room, *RCT* Randomized clinical trial, *RHM* Remote home monitoring

^a^ Study(ies) at high risk of bias

^b^ Small sample size

^c^ Study at high risk of selection bias and presence of confounding variables

^d^ The outcome is an indirect measure of compliance with intervention

**Table 6:** Studies comparing remote home monitoring (phone calls, text messages) to no remote home monitoring

Studies comparing RHM (phone calls, text messages) to no RHM					
**Outcomes**	**№ of participants** **(studies)**	**Certainty of the evidence** **(GRADE)**	Relative effect* (95% CI)	**Anticipated absolute effects**	
				**Risk with no RHM**	**Risk difference with RHM (phone calls, text messages)**
COPD Assessment Test (CAT) scores at the end of monitoring period	106 (1 RCT)	⨁⨁◯◯ LOW ^a,b^	not pooled	not pooled	not pooled
Patient experience- Overall satisfaction at the end of monitoring period—not measured	-	**-**	-	-	-
Average number of exacerbations—not measured	-	**-**	-	-	-
Average number of ER visits due to COPD—not measured	-	**-**	-	-	-
Average number of hospital admissions due to COPD	168 (1 RCT)	⨁⨁◯◯ LOW ^a,b^	not pooled	not pooled	not pooled
Adherence as the proportion of participants who completed the study	168 (1 RCT)	⨁◯◯◯ VERY LOW ^a,b,c^	not pooled	not pooled	not pooled

^*^The risk in the intervention group (and its 95% confidence interval) is based on the assumed risk in the comparison group and the relative effect of the intervention (and its 95% CI)

CI: confidence interval; COPD: chronic obstructive pulmonary disease; ER: emergency room; RCT: randomized clinical trial; RHM: remote home monitoring

^a^ Study at high risk of bias

^b^ Small sample size

^c^ Outcome is an indirect measure of compliance with intervention

### Summary results of effectiveness

#### Health-related quality of life (HRQoL)

##### *RHM (smartphones, apps, tablets) versus no RHM*

Two studies [20, 23] that measured changes in HRQoL from baseline using the CCQ reported no statistically significant differences between groups. Across studies [19–21, 23] that used other HRQoL measures (SF-36, NCSI, and EQ-5D), there were no statistically significant differences between changes in the two groups.

##### *RHM (dedicated monitoring devices) versus no RHM*

None of the studies comparing RHM with no RHM showed a statistically significant difference between groups in the change in HRQoL over time, regardless of the instrument used (CAT, CRQ, SGRQ or EQ5D) [25, 27, 29, 33, 38].

##### RHM with feedback vs RHM without feedback

After 6 months, CAT scores had statistically significantly improved within both groups in the cross-over RCT suggesting that the feedback component had minimal to no effect on HRQoL [37] (details are presented in online supplementary Tables S8, S9 and S10).

#### Patient experience

##### *RHM (smartphones, apps, tablets) versus no RHM*

Three studies [19, 20, 22] that examined patient experiences and satisfaction with RHM demonstrated comparably high satisfaction levels.

##### *RHM (dedicated monitoring devices) versus no RHM*

Seven studies [24, 27, 29–31, 33, 35] explored patients’ experiences with the dedicated monitoring devices. Few difficulties with the devices were reported. In general, patients felt the technology was easy to operate and were satisfied with the support received when troubleshooting clinical and technical problems. Six studies [24, 27, 29–31, 35] assessed perceived benefits related to RHM. They included: better control over/management of their disease, less anxiety, improved ability to cope with their disease, and reduced burden on family members. In all five studies [24, 30, 31, 33, 35] that measured overall satisfaction, the proportion of patients satisfied was high – at least 80% were reported (details presented in online supplementary Table S11).

#### Frequency of exacerbations

##### *RHM (smartphones, apps, tablets) versus no RHM*

No difference was reported [20, 21].

##### *RHM (dedicated monitoring devices) versus no RHM*

One study [28] reported a statistically significantly higher number in the no RHM group, but the larger study [34] found no difference between groups.

#### Healthcare resource utilization (hospital admissions, ER visits, and physician visits)

##### *RHM (smartphones, apps, tablets) versus no RHM*

The impact of RHM on healthcare resource utilization was assessed using numbers of hospital admissions due to COPD [19–21], ER visits [19, 22], and physician visits among patients who received or did not receive RHM [19, 20]. These were similar between groups (detailed description in online supplementary Table S12).

##### *RHM (dedicated monitoring devices) versus no RHM*

Nine studies [24–28, 30, 31, 33, 34, 38] assessed the extent to which RHM with dedicated monitoring devices affected COPD-related hospitalizations. In seven studies, values were similar between groups [24, 25, 27, 28, 30, 31, 33, 38]. However in two studies [26, 34], there were statistically significantly fewer admissions in the RHM group. Of the five studies [25, 27–29, 33, 38] measuring visits to specialists or primary care physicians, four [25, 27, 29, 33, 38] found no statistically significant differences between groups in specialist or primary care physician visits. In one study [28], however, the number of visits to a primary care physician was higher among patients who did not receive RHM.

##### *RHM with feedback vs RHM without feedback*

One study compared the total number of COPD-related hospital admissions over 8 months between treatment groups. The group who received continuous feedback on self-reported monitoring data from a healthcare provider had a statistically significantly lower number of admissions (over 8 months) than the group who did not [36].

#### Adherence to/compliance with treatment

##### *RHM (smartphones, apps, tablets) versus no RHM*

**Fig. 6:**
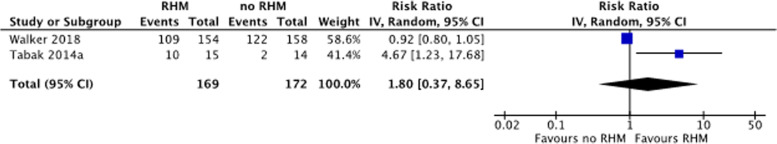
Forest plot of risk ratios for treatment adherence at 9 months of follow-up

In four studies [19–21, 23], adherence with treatment appeared to be similar between groups, but in the fifth study [22], it was almost 5 times higher in the RHM group than in the comparator group (no RHM). Risk ratios for the two studies [21, 23] demonstrated conflicting results (Fig. 6).

##### *RHM (dedicated monitoring devices) versus no RHM*

**Fig. 7:**
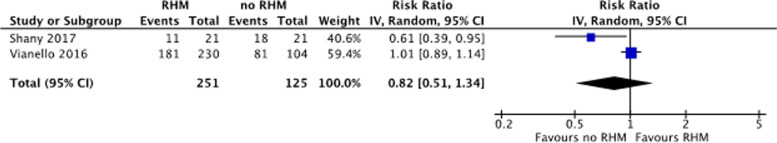
Forest plot of risk ratios for treatment adherence at 12 months of follow-up

In the studies, adherence with treatment appeared to be similar between groups. The exception was a small 12-month study [24] of 21 patients who received RHM and 21 patients who had usual care (no RHM) (Fig. 7) [24, 25].

##### *RHM with feedback vs RHM without feedback*

One study [36] reported a 76% compliance for RHM and 68% for no RHM, but there was no information on the statistical significance of the difference.

#### Safety

One study [19] (RHM using smartphones, apps or tablets vs. no RHM) reported data on adverse events, and no statistically significant differences between treatment groups were found. Eight studies [20, 21, 24–26, 29, 33, 35, 38] reported deaths from all causes and were similar between treatment groups.

#### Exercise capacity and activity levels

##### *RHM (smartphones, apps, tablets) versus no RHM*

Exercise capacity and activity levels improved statistically significantly in the RHM group, but the between groups difference was not statistically significant [19].

##### *RHM (dedicated monitoring devices) versus no RHM*

Patients who received RHM statistically significantly increased the 6-min walk distance, but those in the no RHM group did not [28].

##### *RHM with feedback vs RHM without feedback*

Total leisure activity at 6 months in patients who received RHM with feedback was statistically significantly higher than in the group without feedback (details presented in online supplementary Table S14) [37].

#### Mental health

##### *RHM (smartphones, apps, tablets) versus no RHM*

Neither study reported statistically significant changes in POMS or PHQ-9 (tension-anxiety and depression) scores within or between groups after 6 months [19, 21].

##### *RHM (dedicated monitoring devices) versus no RHM*

No statistically significant differences in HADS values were reported among patients who received RHM compared to those who did not [25, 33] (details presented in online supplementary Table S15).

#### Self-efficacy

##### *RHM (smartphones, apps, tablets) versus no RHM*

Neither of two studies reported statistically significant differences in self-efficacy measures between the RHM and usual care groups at baseline or at the end of the follow-up period [19, 20] (details are presented in online supplementary Table S16).

#### Cost per patient

##### *RHM (smartphones, apps, tablets) versus no RHM*

In the single study [21] reporting per patient costs with and without RHM, no statistically significant differences were seen between groups.

##### *RHM (dedicated monitoring devices) versus no RHM*

Two studies [24, 32] compared the cost of hospital admission and one [30] compared all costs (from 12 months prior to and 6 months after the start of RHM). All concluded that there were no statistically significant difference between groups (details presented in online supplementary Table S17).

#### Provider experience

None of the included studies reported on providers' experiences involved in RHM (smartphones, apps, tablets) versus no RHM and RHM with feedback vs RHM without feedback comparisons.

##### *RHM (dedicated monitoring devices) versus no RHM*

Two studies [24, 29] reported findings from surveys designed to obtain feedback from providers. Perceptions around the dedicated home monitoring device's usability and value improved as provider experience increased; however, only six providers participated in the two studies (details presented in online supplementary Table S18).

### Lung function and symptoms

#### *RHM (smartphones, apps, tablets) versus no RHM*

No statistically significant differences in baseline or follow-up scores in validated measures in 2 studies [19, 20].

#### *RHM (dedicated monitoring devices) versus no RHM*

In the two studies [28, 31], no statistically significant differences were found in predicted values for FEV_1_ at baseline and at the end of follow-up between groups (details presented in online supplementary Table S19).

## Discussion

Several aspects distinguish this work from previously published literature reviews [3, 41–46]. This review yielded more studies due to the broader inclusion criteria of home monitoring technology and its components, outcomes, and types of included studies. For example, previous systematic reviews included small numbers of studies (between 3 [41] and 10 [42]). Further, previous reviews measured a relatively small number of outcomes [45], had unclearly defined outcomes [3], had a substantive difference between defined and measured outcomes [41], or considered satisfaction from the patient perspective only [42, 46]. In the current review, in addition to defining and measuring outcomes such as adherence (exercise, self-management, diary, and medication use), exacerbation, hospitalizations, and patient satisfaction, the focus was also on including other outcomes relevant to health services and program planning such as safety, cost per patient and provider experience. Finally, this study extends previous reviews [42, 47, 48] by synthesizing findings according to type of technology and feedback provided.

This review concludes that HRQoL was not significantly improved with RHM as compared to usual care, regardless of monitoring technology; this finding is similar to previous reviews [42, 48, 49]. HRQoL is a complex construct, and while programs such as pulmonary rehabilitation consistently show improvement in HRQoL in COPD [50, 51], other disease management interventions (e.g., pharmaceutical care, patient education and action plan) do not consistently improve HRQoL in this disease [52, 53]. Unfortunately, no study was identified that used RHM during pulmonary rehabilitation. Instead, studies that included elements of pulmonary rehabilitation such as patient education, and/or exercise in both usual care and RHM groups, showed no greater benefit in HRQoL within the RHM group [19, 20, 22, 23]. These findings would suggest that regardless of the disease management program used, RHM did not improve HRQoL over and above usual care.

Remote home monitoring has the potential to improve disease self-management by making patients more aware of day-to-day changes in their symptoms and physical function [54, 55], thus improving disease management and reducing the risk of exacerbation. While previous studies have shown a significant reduction in health care utilization in COPD patients using self-management programs [56–58], this review found no consistent impact of RHM on patient self-efficacy, physician visits, ER visits or hospitalizations. Behaviour change is required for proper disease self-management, and time is required for patients to adopt and adhere to new behaviours. Most trials were under 12 months, and there may have been insufficient time to develop appropriate behaviour change that would lead to better disease management and reduced health care utilization.

Numerous studies have evaluated the impact of disease management programs in COPD, but due to heterogeneity in content, duration, and frequency of follow-ups, it is challenging to identify the key components of these programs. This review suggests that COPD related hospital admissions improved when RHM was coupled with feedback from healthcare professionals. No RCTs have investigated patient-provider communication in COPD specifically, but within other chronic disease states, more frequent and positive patient-provider communication was associated with improved health outcome and higher levels of self-efficacy [59, 60]. A recent qualitative study aimed to explore the views of patients and professionals on telemonitoring found that patients and health care professionals considered relationship-based care important in COPD telemonitoring services [61]. Therefore, RHM that facilitates regular communication with a healthcare professional appears to be important.

While considered usual care, patients with COPD are often not referred to pulmonary rehabilitation. Several barriers, including lack of available programs and travel/transportation needs, prevent patients from attending conventional centre-based rehabilitation programs [62–64]. Home-based alternatives are needed, but these are currently underdeveloped and the complexity of COPD patients raises concerns regarding patient safety. Future studies should aim to evaluate the additional benefits of RHM in patients undergoing (virtual) pulmonary rehabilitation. Further work should also evaluate patient behaviour to determine if RHM is effective at changing key behaviours that are foundational to improved disease management.

## Limitations

This systematic review has several limitations. One drawback of this review is the lack of its protocol registration in the PROSPERO database as recommended by guidelines [11, 12]. Any protocol changes were documented and discussed within the research group to minimize bias.Second, there is the possible risk of bias due to missing information in the included studies. Furthermore, included studies provided limited descriptions of the study randomization process, and the studies varied in components of the interventions. Third, the study was restricted to English language studies, which might have led to the exclusion of possibly relevant studies. In addition, it was not possible to perform a meta-analysis on outcomes due to a high level of heterogeneity and limited data. Finally, there is the possibility of an impact on the findings by unpublished negative studies.

## Conclusion

By applying objective, high-quality methods for gathering and synthesizing information from primary studies, this systematic review was conducted to review evidence from 19 studies, 17 of them RCTs, of remote home monitoring effectiveness in patients with COPD. Although a considerable amount of evidence to the effectiveness of RHM exists, due to heterogeneity of care settings, RHM components and the low-quality evidence, they should be interpreted with caution.

## Supplementary Information

**Additional file 1: Table S1**. Literaturesearch results. **Table S2**.Characteristics of included studies. **TableS3**. Characteristics of participants included in the studies. **Table S4**. Remote home monitoringcomponents. **Table S5**. Description ofremote home monitoring programs and technology. **Table S6**. Remote home monitoring components. **Table S7**. Risk of bias. **TableS8**. Health-related quality of life - CAT, CCQ and other instruments. **Table S9**. Health related quality oflife – CRQ. **Table S10**. Healthrelated quality of life – SGRQ. **TableS11**. Patient experience and satisfaction with RHM. **Table S12**. Frequency of exacerbations, Hospital admissions, ERvisits and physician visits. **Table S13**.Adverse events and deaths during the follow-up period. **Table S14**. Exercise capacity and activity levels. **Table S15**. Mental health. **Table S16**. Self-efficacy. **Table S17**. Cost per patient. **Table S18**. Provider experience. **Table S19**. Lung function.

## Data Availability

All data relevant to the study are included in the article or uploaded as supplementary information.
