# Supplementary material for: Effectiveness of remote home monitoring for patients with Chronic Obstructive Pulmonary Disease (COPD): systematic review
Source: BMC Health Serv Res. 2022 May 14;22:646. doi: 10.1186/s12913-022-07938-y (PMC9107164; doi:10.1186/s12913-022-07938-y)
Supplement: Supplementary file 1 — Additional file 1: Table S1. Literaturesearch results. Table S2.Characteristics of included studies. TableS3. Characteristics of participants included in the studies. Table S4. Remote home monitoringcomponents. Table S5. Description ofremote home monitoring programs and technology. Table S6. Remote home monitoring components. Table S7. Risk of bias. TableS8. Health-related quality of life - CAT, CCQ and other instruments. Table S9. Health related quality oflife – CRQ. Table S10. Healthrelated quality of life – SGRQ. TableS11. Patient experience and satisfaction with RHM. Table S12. Frequency of exacerbations, Hospital admissions, ERvisits and physician visits. Table S13.Adverse events and deaths during the follow-up period. Table S14. Exercise capacity and activity levels. Table S15. Mental health. Table S16. Self-efficacy. Table S17. Cost per patient. Table S18. Provider experience. Table S19. Lung function. [file 12913_2022_7938_MOESM1_ESM.docx]

# Supplemental materials

[Table S1: Literature search results 2](#_Toc98968353)

[Table S2. Characteristics of included studies 24](#_Toc98968354)

[Table S3. Characteristics of participants included in the studies 32](#_Toc98968355)

[Table S4: Remote home monitoring components 35](#_Toc98968356)

[Table S5: Description of remote home monitoring programs and technology 39](#_Toc98968357)

[Table S6: Remote home monitoring components 46](#_Toc98968358)

[Table S7: Risk of bias 51](#_Toc98968359)

[Table S8. Health-related quality of life - CAT, CCQ and other instruments 58](#_Toc98968360)

[Table S9. Health related quality of life - CRQ 63](#_Toc98968361)

[Table S10. Health related quality of life - SGRQ 65](#_Toc98968362)

[Table S11. Patient experience and satisfaction with RHM 67](#_Toc98968363)

[Table S12. Frequency of exacerbations, Hospital admissions, ER visits and physician visits 71](#_Toc98968364)

[Table S13. Adverse events and deaths during the follow-up period 78](#_Toc98968365)

[Table S14. Exercise capacity and activity levels 80](#_Toc98968366)

[Table S15. Mental health 82](#_Toc98968367)

[Table S16. Self-efficacy 84](#_Toc98968368)

[Table S17. Cost per patient 87](#_Toc98968369)

[Table S18. Provider experience 91](#_Toc98968370)

[Table S19. Lung function 93](#_Toc98968371)

Table S1: Literature search results

2020 Mar 11

**Ovid Multifile**

Database: Ovid MEDLINE(R) ALL <1946 to March 11, 2020>, Embase <1974 to 2020 March 11>

Search Strategy:

--------------------------------------------------------------------------------

1 Lung Diseases, Obstructive/ (100081)

2 exp Pulmonary Disease, Chronic Obstructive/ (184050)

3 exp Emphysema/ or exp Pulmonary Emphysema/ (68715)

4 ((chronic adj2 obstructi*) and (pulmonary or airway* or air way* or lung$1 or airflow* or air flow*)).tw,kf. (130862)

5 (COPD or COAD).tw,kf. (131271)

6 (chronic adj2 bronchitis).tw,kf. (23757)

7 emphysema*.tw,kf. (60195)

8 exp Respiratory Therapy/ (116699)

9 respirat* therap*.tw,kf. (6939)

10 ((pulmonary or respirat*) adj3 rehab*).tw,kf. (11693)

11 or/1-10 [COPD, Incl. RESPIRATORY THERAPY/REHAB] (437301)

12 exp Heart Failure/ (606936)

13 ((cardiac or cardial or heart or myocardia* or myo-cardia*) adj1 (backward failure? or decompensat* or failure? or incompetenc* or insufficienc* or standstill or stand still)).tw,kf. (473579)

14 ((HF or CHF) and heart).tw,kf. (110630)

15 ((cardio-renal or cardiorenal or reno-cardiac or renocardiac) adj1 syndrome?).tw,kf. (2894)

16 or/12-15 [HEART FAILURE] (764256)

17 11 or 16 [COPD, HEART FAILURE] (1174117)

18 Monitoring, Ambulatory/ (18868)

19 Electrocardiography, Ambulatory/ (11021)

20 Remote Consultation/ (13381)

21 Telemedicine/ (44245)

22 Telerehabilitation/ (1047)

23 Remote Sensing Technology/ (11232)

24 Telemetry/ (26888)

25 ((remote* or virtual) adj3 interrogat*).tw,kf. (188)

26 ((remote* or virtual*) adj3 (care or healthcare or health care)).tw,kf. (4465)

27 ((remote* or virtual*) adj3 consult*).tw,kf. (1647)

28 ((remote* or virtual*) adj3 (followup? or follow-up?)).tw,kf. (1122)

29 ((remote* or virtual*) adj3 interven*).tw,kf. (1902)

30 ((remote* or virtual*) adj3 manag*).tw,kf. (1939)

31 ((remote* or virtual*) adj3 monitor*).tw,kf. (9229)

32 ((remote* or virtual*) adj3 outpatient*).tw,kf. (222)

33 ((outpatient* or out-patient* or ambulator* or home? or homebased or home-based) adj3 (manag* or monitor*)).tw,kf. (62616)

34 ((remote* or virtual*) adj3 rehab*).tw,kf. (1089)

35 ((remote* or virtual*) adj3 surveillance*).tw,kf. (339)

36 ((remote* or virtual*) adj3 visit*).tw,kf. (911)

37 (teleconsult* or tele-consult* or econsult* or e-consult*).tw,kf. (3490)

38 (telefollowup? or telefollow-up? or tele-followup? or tele-follow-up? or efollowup? or efollow-up? or e-followup? or e-follow-up?).tw,kf. (71)

39 (telecare or tele-care or telehealth or tele-health or telehealthcare or tele-healthcare or telemedicine or tele-medicine).tw,kf. (34368)

40 (teleinterven* or tele-interven* or einterven* or e-interven*).tw,kf. (432)

41 (telemanag* or tele-manag* or emanag* or e-manag*).tw,kf. (275)

42 (telemonitor* or tele-monitor* or emonitor* or e-monitor*).tw,kf. (4230)

43 (telerehab* or tele-rehab* or erehab* or e-rehab*).tw,kf. (1890)

44 (telesurveillance* or tele-surveillance* or esurveillance* or e-surveillance*).tw,kf. (92)

45 (televisit* or tele-visit* or evisit* or e-visit*).tw,kf. (246)

46 ((dynamic* or Holter) adj3 (manag* or monitor*)).tw,kf. (29466)

47 (ecare or e-care or ehealth or e-health or ehealthcare or e-healthcare or emedicine or e-medicine).tw,kf. (12000)

48 (mobile care or mcare or m-care or mobile health or mhealth or m-health or mobile healthcare or mhealthcare or m-healthcare).tw,kf. (12272)

49 ((internet* or app or apps or smarthome* or smart home* or smartphone* or smart phone* or mobile-based or e-mail* or email* or electronic mail* or emedicine or e-medicine or technolog* or computer* or digital* or webbased or web-based or webdeliver* or web-deliver* or online) adj3 (manag* or monitor*)).tw,kf. (37753)

50 (remote sensing or remote sensor*).tw,kf. (14352)

51 (telemetr* or tele-metr*).tw,kf. (22217)

52 or/18-51 [REMOTE MONITORING] (290634)

53 Smart Breath Analy#er?.tw,kf. (2)

54 Monitoring, Physiologic/ and (exp Lung/ or (breath* or lung* or pulmonar* or respirat*).tw,kf.) (7896)

55 ((electronic inhal* adj2 monitoring device?) or EIMD or EIMDs).tw,kf. (352)

56 exp Cardiac Pacing, Artificial/ (66725)

57 (artificial* adj2 card* pac*).tw,kf. (322)

58 (cardiac adj (resynchroni* or re-synchroni*)).tw,kf. (22290)

59 exp Pacemaker, Artificial/ (59705)

60 (pacemak* or pace-mak*).tw,kf. (91067)

61 Defibrillators, Implantable/ (33737)

62 (implant* adj3 defibrillat*).tw,kf. (38737)

63 (implant* adj3 device*).tw,kf. (49236)

64 ((cardiac* or cardio*) adj3 implant*).tw,kf. (46404)

65 (ICD or ICDs).tw,kf. and (exp Heart/ or (cardio* or cardiac* or coronar* or heart? or defibrillat* or fibrillat* or implant*).tw,kf.) (42149)

66 (AICD or AICDs or S-ICD or S-ICDs or CIED or CIEDs).tw,kf. (7764)

67 Electrophysiology/ (159425)

68 Cardiac Electrophysiology/ (19209)

69 (electrophysiolog* or electro-physiolog*).tw,kf. (243143)

70 Monitoring, Physiologic/ and (exp Heart/ or (cardio* or cardiac* or coronar* or heart? or defibrillat* or fibrillat* or implant*).tw,kf.) (15297)

71 CardioMEMS.tw,kf. (238)

72 or/53-71 [ICDs/PHYSIOLOGIC MONITORING] (610964)

73 52 or 72 [REMOTE/HEART MONITORING] (884944)

74 17 and 73 [COPD, HEART FAILURE - REMOTE/HEART MONITORING] (88561)

75 Home Care Services/ (82043)

76 Home Care Services, Hospital-Based/ (61620)

77 Home Health Nursing/ (57844)

78 Home Nursing/ (66034)

79 ((domicil* or home?) adj3 (care or health care or healthcare)).tw,kf. (75327)

80 ((domicil* or home?) adj3 (assist* or manag* or monitor* or network* or program* or rehab* or resourc* or servic* or support*)).tw,kf. (67102)

81 ((domicil* or home?) adj3 model*).tw,kf. (4331)

82 homecare.tw,kf. (2808)

83 (home-based or homebased or domicile-based).tw,kf. (24081)

84 ((domicil* or home?) adj3 dwelling).tw,kf. (1609)

85 ((domicil* or home?) adj3 environment*).tw,kf. (17205)

86 ((domicil* or home?) adj3 session?).tw,kf. (1717)

87 ((domicil* or home?) adj3 setting?).tw,kf. (14618)

88 home nurs*.tw,kf. (3565)

89 "at home".tw,kf. (114745)

90 "in home".tw,kf. (24231)

91 (patient* home? or patient* domicil*).tw,kf. (7318)

92 (home* adj2 PR).tw,kf. (207)

93 HBPR.tw,kf. (57)

94 home?.ti. (164701)

95 Self Management/ (47951)

96 Self Care/ (89427)

97 limit 96 to yr="2015-2017" (17756)

98 ((care or manag*) adj2 self).tw,kf. (90157)

99 ((care or manag*) adj2 himself).tw,kf. (172)

100 ((care or manag*) adj2 herself).tw,kf. (170)

101 ((care or manag*) adj2 themsel*).tw,kf. (2874)

102 or/75-95,97-101 [HOME-BASED] (481729)

103 74 and 102 [COPD, HEART FAILURE - REMOTE/HEART MONITORING - HOME-BASED] (4498)

104 exp Animals/ not Humans/ (16989375)

105 103 not 104 [ANIMAL-ONLY REMOVED] (3018)

106 (comment or editorial or news or newspaper article).pt. (2038264)

107 (letter not (letter and randomized controlled trial)).pt. (2163952)

108 105 not (106 or 107) [OPINION PIECES REMOVED] (2951)

109 limit 108 to english (2750)

110 109 use medall [MEDLINE RECORDS] (1533)

111 obstructive airway disease/ (2054)

112 chronic obstructive lung disease/ (168119)

113 emphysema/ or exp lung emphysema/ (42744)

114 ((chronic adj2 obstructi*) and (pulmonary or airway* or air way* or lung$1 or airflow* or air flow*)).tw,kw. (132380)

115 (COPD or COAD).tw,kw. (133171)

116 (chronic adj2 bronchitis).tw,kw. (24048)

117 emphysema*.tw,kw. (60335)

118 exp respiratory care/ (4039)

119 respirat* therap*.tw,kw. (7343)

120 pulmonary rehabilitation/ (6513)

121 ((pulmonary or respirat*) adj3 rehab*).tw,kw. (11926)

122 or/111-121 [COPD, Incl. RESPIRATORY THERAPY/REHAB] (318369)

123 exp heart failure/ (606936)

124 ((cardiac or cardial or heart or myocardia* or myo-cardia*) adj1 (backward failure? or decompensat* or failure? or incompetenc* or insufficienc* or standstill or stand still)).tw,kw. (477069)

125 ((HF or CHF) and heart).tw,kw. (110854)

126 ((cardio-renal or cardiorenal or reno-cardiac or renocardiac) adj1 syndrome?).tw,kw. (3019)

127 or/123-126 [HEART FAILURE] (765449)

128 122 or 127 [COPD, HEART FAILURE] (1059601)

129 ambulatory monitoring/ (19379)

130 ambulatory electrocardiography/ (11021)

131 telemedicine/ (44245)

132 telecardiology/ (238)

133 teleconsultation/ (13871)

134 telemonitoring/ (2974)

135 telerehabilitation/ (1047)

136 telehealth/ (27433)

137 remote sensing/ (8481)

138 telemetry/ (26888)

139 ((remote* or virtual) adj3 interrogat*).tw,kw. (190)

140 ((remote* or virtual*) adj3 (care or healthcare or health care)).tw,kw. (4491)

141 ((remote* or virtual*) adj3 consult*).tw,kw. (1689)

142 ((remote* or virtual*) adj3 (followup? or follow-up?)).tw,kw. (1147)

143 ((remote* or virtual*) adj3 interven*).tw,kw. (1929)

144 ((remote* or virtual*) adj3 manag*).tw,kw. (1947)

145 ((remote* or virtual*) adj3 monitor*).tw,kw. (9350)

146 ((remote* or virtual*) adj3 outpatient*).tw,kw. (226)

147 ((outpatient* or out-patient* or ambulator* or home? or homebased or home-based) adj3 (manag* or monitor*)).tw,kw. (63862)

148 ((remote* or virtual*) adj3 surveillance*).tw,kw. (356)

149 ((remote* or virtual*) adj3 visit*).tw,kw. (914)

150 (teleconsult* or tele-consult* or econsult* or e-consult*).tw,kw. (3598)

151 (telefollowup? or telefollow-up? or tele-followup? or tele-follow-up? or efollowup? or efollow-up? or e-followup? or e-follow-up?).tw,kw. (72)

152 (telecare or tele-care or telehealth or tele-health or telehealthcare or tele-healthcare or telemedicine or tele-medicine).tw,kw. (37460)

153 (teleinterven* or tele-interven* or einterven* or e-interven*).tw,kw. (440)

154 (telemanag* or tele-manag* or emanag* or e-manag*).tw,kw. (275)

155 (telemonitor* or tele-monitor* or emonitor* or e-monitor*).tw,kw. (4392)

156 (telerehab* or tele-rehab* or erehab* or e-rehab*).tw,kw. (2022)

157 (telesurveillance* or tele-surveillance* or esurveillance* or e-surveillance*).tw,kw. (95)

158 (televisit* or tele-visit* or evisit* or e-visit*).tw,kw. (248)

159 ((dynamic* or Holter) adj3 (manag* or monitor*)).tw,kw. (29645)

160 (ecare or e-care or ehealth or e-health or ehealthcare or e-healthcare or emedicine or e-medicine).tw,kw. (13738)

161 (mobile care or mcare or m-care or mobile health or mhealth or m-health or mobile healthcare or mhealthcare or m-healthcare).tw,kw. (13755)

162 ((internet* or app or apps or smarthome* or smarthome* or smartphone* or smart phone* or mobile-based or e-mail* or email* or electronic mail* or emedicine or e-medicine or technolog* or computer* or digital* or webbased or web-based or webdeliver* or web-deliver* or online) adj3 (manag* or monitor*)).tw,kw. (37964)

163 (remote sensing or remote sensor*).tw,kw. (15357)

164 (telemetr* or tele-metr*).tw,kw. (22785)

165 or/129-164 [REMOTE MONITORING] (296131)

166 Smart Breath Analy#er?.tw,kw. (2)

167 physiologic monitoring/ and (exp lung/ or (breath* or lung* or pulmonar* or respirat*).tw,kw.) (8196)

168 ((electronic inhal* adj2 monitoring device?) or EIMD or EIMDs).tw,kw. (356)

169 exp heart pacing/ (42229)

170 (artificial* adj2 card* pac*).tw,kw. (429)

171 (cardiac adj (resynchroni* or re-synchroni*)).tw,kw. (22670)

172 exp artificial heart pacemaker/ (32765)

173 (pacemak* or pace-mak*).tw,kw. (92752)

174 exp implantable cardioverter defibrillator/ (54341)

175 (implant* adj3 defibrillat*).tw,kw. (39524)

176 (implant* adj3 device*).tw,kw. (49531)

177 ((cardiac* or cardio*) adj3 implant*).tw,kw. (47362)

178 (ICD or ICDs).tw,kw. and (exp Heart/ or (cardio* or cardiac* or coronar* or heart? or defibrillat* or fibrillat* or implant*).tw,kw.) (42616)

179 (AICD or AICDs or S-ICD or S-ICDs or CIED or CIEDs).tw,kw. (7871)

180 electrophysiology/ (159425)

181 heart electrophysiology/ (18760)

182 (electrophysiolog* or electro-physiolog*).tw,kw. (250462)

183 physiologic monitoring/ and (exp heart/ or (cardio* or cardiac* or coronar* or heart? or defibrillat* or fibrillat* or implant*).tw,kw.) (15816)

184 CardioMEMS.tw,kw. (251)

185 or/166-184 [ICDs/PHYSIOLOGIC MONITORING] (617845)

186 165 or 185 [REMOTE/HEART MONITORING] (897167)

187 128 and 186 [COPD, HEART FAILURE - REMOTE/HEART MONITORING] (86018)

188 home care/ (92797)

189 home monitoring/ (4492)

190 home oxygen therapy/ (1037)

191 home rehabilitation/ (670)

192 home respiratory care/ (93)

193 visiting nursing service/ (165)

194 ((domicil* or home?) adj3 (care or health care or healthcare)).tw,kw. (75850)

195 ((domicil* or home?) adj3 (assist* or manag* or monitor* or network* or program* or rehab* or resourc* or servic* or support*)).tw,kw. (67453)

196 ((domicil* or home?) adj3 model*).tw,kw. (4338)

197 homecare.tw,kw. (2871)

198 (home-based or homebased or domicile-based).tw,kw. (24177)

199 ((domicil* or home?) adj3 environment*).tw,kw. (17273)

200 ((domicil* or home?) adj3 session?).tw,kw. (1717)

201 ((domicil* or home?) adj3 setting?).tw,kw. (14622)

202 home nurs*.tw,kw. (3679)

203 "at home".tw,kw. (114799)

204 "in home".tw,kw. (24253)

205 (patient* home? or patient* domicil*).tw,kw. (7560)

206 (home* adj2 PR).tw,kw. (207)

207 HBPR.tw,kw. (57)

208 home?.ti. (164701)

209 self care/ (89427)

210 ((care or manag*) adj2 self).tw,kw. (90725)

211 ((care or manag*) adj2 himself).tw,kw. (172)

212 ((care or manag*) adj2 herself).tw,kw. (171)

213 ((care or manag*) adj2 themsel*).tw,kw. (2874)

214 or/188-213 [HOME-BASED] (496370)

215 187 and 214 [COPD, HEART FAILURE - REMOTE/HEART MONITORING - HOME-BASED] (4528)

216 exp animal/ or exp animal experimentation/ or exp animal model/ or exp animal experiment/ or nonhuman/ or exp vertebrate/ (50073713)

217 exp human/ or exp human experimentation/ or exp human experiment/ (38968094)

218 216 not 217 (11107187)

219 215 not 218 [ANIMAL-ONLY REMOVED] (4504)

220 editorial.pt. (1164890)

221 letter.pt. not (letter.pt. and randomized controlled trial/) (2158706)

222 219 not (220 or 221) [OPINION PIECES REMOVED] (4420)

223 limit 222 to english (4204)

224 223 use oemezd [EMBASE RECORDS] (2882)

225 110 or 224 [BOTH DATABASES] (4415)

226 remove duplicates from 225 (3239) [TOTAL UNIQUE RECORDS]

227 226 use medall [MEDLINE UNIQUE RECORDS] (1522)

228 226 use oemezd [EMBASE UNIQUE RECORDS] (1717)

***************************

**EBM Databases**

Database: EBM Reviews - Cochrane Central Register of Controlled Trials <February 2020>, EBM Reviews - Cochrane Database of Systematic Reviews <2005 to March 4, 2020>, EBM Reviews - Database of Abstracts of Reviews of Effects <1st Quarter 2016>, EBM Reviews - Health Technology Assessment <4th Quarter 2016>, EBM Reviews - NHS Economic Evaluation Database <1st Quarter 2016>

Search Strategy:

--------------------------------------------------------------------------------

1 Lung Diseases, Obstructive/ (3007)

2 exp Pulmonary Disease, Chronic Obstructive/ (5770)

3 exp Emphysema/ or exp Pulmonary Emphysema/ (482)

4 ((chronic adj2 obstructi*) and (pulmonary or airway* or air way* or lung$1 or airflow* or air flow*)).ti,ab,kw. (14645)

5 (COPD or COAD).ti,ab,kw. (16415)

6 (chronic adj2 bronchitis).ti,ab,kw. (1879)

7 emphysema*.ti,ab,kw. (1414)

8 exp Respiratory Therapy/ (7880)

9 respirat* therap*.ti,ab,kw. (495)

10 ((pulmonary or respirat*) adj3 rehab*).ti,ab,kw. (2228)

11 or/1-10 [COPD, Incl. RESPIRATORY THERAPY/REHAB] (31916)

12 exp Heart Failure/ (9146)

13 ((cardiac or cardial or heart or myocardia* or myo-cardia*) adj1 (backward failure? or decompensat* or failure? or incompetenc* or insufficienc* or standstill or stand still)).ti,ab,kw. (29695)

14 ((HF or CHF) and heart).ti,ab,kw. (9366)

15 ((cardio-renal or cardiorenal or reno-cardiac or renocardiac) adj1 syndrome?).ti,ab,kw. (107)

16 or/12-15 [HEART FAILURE] (31710)

17 11 or 16 [COPD, HEART FAILURE] (62360)

18 Monitoring, Ambulatory/ (583)

19 Electrocardiography, Ambulatory/ (1208)

20 Remote Consultation/ (436)

21 Telemedicine/ (2128)

22 Telerehabilitation/ (92)

23 Remote Sensing Technology/ (40)

24 Telemetry/ (277)

25 ((remote* or virtual) adj3 interrogat*).ti,ab,kw. (18)

26 ((remote* or virtual*) adj3 (care or healthcare or health care)).ti,ab,kw. (401)

27 ((remote* or virtual*) adj3 consult*).ti,ab,kw. (130)

28 ((remote* or virtual*) adj3 (followup? or follow-up?)).ti,ab,kw. (19)

29 ((remote* or virtual*) adj3 interven*).ti,ab,kw. (767)

30 ((remote* or virtual*) adj3 manag*).ti,ab,kw. (313)

31 ((remote* or virtual*) adj3 monitor*).ti,ab,kw. (967)

32 ((remote* or virtual*) adj3 outpatient*).ti,ab,kw. (32)

33 ((outpatient* or out-patient* or ambulator* or home? or homebased or home-based) adj3 (manag* or monitor*)).ti,ab,kw. (38169)

34 ((remote* or virtual*) adj3 rehab*).ti,ab,kw. (316)

35 ((remote* or virtual*) adj3 surveillance*).ti,ab,kw. (29)

36 ((remote* or virtual*) adj3 visit*).ti,ab,kw. (152)

37 (teleconsult* or tele-consult* or econsult* or e-consult*).ti,ab,kw. (546)

38 (telefollowup? or telefollow-up? or tele-followup? or tele-follow-up? or efollowup? or efollow-up? or e-followup? or e-follow-up?).ti,ab,kw. (0)

39 (telecare or tele-care or telehealth or tele-health or telehealthcare or tele-healthcare or telemedicine or tele-medicine).ti,ab,kw. (3379)

40 (teleinterven* or tele-interven* or einterven* or e-interven*).ti,ab,kw. (102)

41 (telemanag* or tele-manag* or emanag* or e-manag*).ti,ab,kw. (53)

42 (telemonitor* or tele-monitor* or emonitor* or e-monitor*).ti,ab,kw. (1042)

43 (telerehab* or tele-rehab* or erehab* or e-rehab*).ti,ab,kw. (514)

44 (telesurveillance* or tele-surveillance* or esurveillance* or e-surveillance*).ti,ab,kw. (8)

45 (televisit* or tele-visit* or evisit* or e-visit*).ti,ab,kw. (25)

46 ((dynamic* or Holter) adj3 (manag* or monitor*)).ti,ab,kw. (2054)

47 (ecare or e-care or ehealth or e-health or ehealthcare or e-healthcare or emedicine or e-medicine).ti,ab,kw. (924)

48 (mobile care or mcare or m-care or mobile health or mhealth or m-health or mobile healthcare or mhealthcare or m-healthcare).ti,ab,kw. (1544)

49 ((internet* or app or apps or smarthome* or smart home* or smartphone* or smart phone* or mobile-based or e-mail* or email* or electronic mail* or emedicine or e-medicine or technolog* or computer* or digital* or webbased or web-based or webdeliver* or web-deliver* or online) adj3 (manag* or monitor*)).ti,ab,kw. (3901)

50 (remote sensing or remote sensor*).ti,ab,kw. (224)

51 (telemetr* or tele-metr*).ti,ab,kw. (594)

52 or/18-51 [REMOTE MONITORING] (52272)

53 Smart Breath Analy#er?.ti,ab,kw. (0)

54 Monitoring, Physiologic/ and (exp Lung/ or (breath* or lung* or pulmonar* or respirat*).ti,ab,kw.) (401)

55 ((electronic inhal* adj2 monitoring device?) or EIMD or EIMDs).ti,ab,kw. (80)

56 exp Cardiac Pacing, Artificial/ (1496)

57 (artificial* adj2 card* pac*).ti,ab,kw. (41)

58 (cardiac adj (resynchroni* or re-synchroni*)).ti,ab,kw. (1930)

59 exp Pacemaker, Artificial/ (788)

60 (pacemak* or pace-mak*).ti,ab,kw. (2797)

61 Defibrillators, Implantable/ (1087)

62 (implant* adj3 defibrillat*).ti,ab,kw. (3171)

63 (implant* adj3 device*).ti,ab,kw. (2159)

64 ((cardiac* or cardio*) adj3 implant*).ti,ab,kw. (3690)

65 (ICD or ICDs).ti,ab,kw. and (exp Heart/ or (cardio* or cardiac* or coronar* or heart? or defibrillat* or fibrillat* or implant*).ti,ab,kw.) (2265)

66 (AICD or AICDs or S-ICD or S-ICDs or CIED or CIEDs).ti,ab,kw. (185)

67 Electrophysiology/ (498)

68 Cardiac Electrophysiology/ (5)

69 (electrophysiolog* or electro-physiolog*).ti,ab,kw. (3676)

70 Monitoring, Physiologic/ and (exp Heart/ or (cardio* or cardiac* or coronar* or heart? or defibrillat* or fibrillat* or implant*).ti,ab,kw.) (811)

71 CardioMEMS.ti,ab,kw. (24)

72 or/53-71 [ICDs/PHYSIOLOGIC MONITORING] (14882)

73 52 or 72 [REMOTE/HEART MONITORING] (65649)

74 17 and 73 [COPD, HEART FAILURE - REMOTE/HEART MONITORING] (7348)

75 Home Care Services/ (2000)

76 Home Care Services, Hospital-Based/ (289)

77 Home Health Nursing/ (8)

78 Home Nursing/ (296)

79 ((domicil* or home?) adj3 (care or health care or healthcare)).ti,ab,kw. (6030)

80 ((domicil* or home?) adj3 (assist* or manag* or monitor* or network* or program* or rehab* or resourc* or servic* or support*)).ti,ab,kw. (10477)

81 ((domicil* or home?) adj3 model*).ti,ab,kw. (422)

82 homecare.ti,ab,kw. (159)

83 (home-based or homebased or domicile-based).ti,ab,kw. (6571)

84 ((domicil* or home?) adj3 dwelling).ti,ab,kw. (229)

85 ((domicil* or home?) adj3 environment*).ti,ab,kw. (1403)

86 ((domicil* or home?) adj3 session?).ti,ab,kw. (1340)

87 ((domicil* or home?) adj3 setting?).ti,ab,kw. (1559)

88 home nurs*.ti,ab,kw. (325)

89 "at home".ti,ab,kw. (40266)

90 "in home".ti,ab,kw. (40165)

91 (patient* home? or patient* domicil*).ti,ab,kw. (1476)

92 (home* adj2 PR).ti,ab,kw. (44)

93 HBPR.ti,ab,kw. (5)

94 home?.ti. (11692)

95 Self Management/ (476)

96 Self Care/ (4187)

97 limit 96 to yr="2015-2017" [Limit not valid in DARE; records were retained] (968)

98 ((care or manag*) adj2 self).ti,ab,kw. (13341)

99 ((care or manag*) adj2 himself).ti,ab,kw. (7)

100 ((care or manag*) adj2 herself).ti,ab,kw. (8)

101 ((care or manag*) adj2 themsel*).ti,ab,kw. (152)

102 or/75-95,97-101 [HOME-BASED SERVICES] (54390)

103 74 and 102 [COPD, HEART FAILURE - REMOTE/HEART MONITORING - HOME BASED] (1453)

104 103 use coch [COCHRANE DSR RECORDS] (5)

105 103 use cctr [CENTRAL RECORDS] (1415)

106 103 use dare [DARE RECORDS] (14)

107 103 use clhta [HTA RECORDS] (3)

108 103 use cleed [NHS EED RECORDS] (16)

***************************

**CINAHL**

| # | Query | Limiters/Expanders | Results |
| --- | --- | --- | --- |
| S103 | S73 AND S100 | Limiters - Exclude MEDLINE records  Expanders - Apply equivalent subjects  Search modes - Find all my search terms | 456 |
| S102 | S73 AND S100 | Expanders - Apply equivalent subjects  Narrow by Language: - english  Search modes - Find all my search terms | 1,095 |
| S101 | S73 AND S100 | Expanders - Apply equivalent subjects  Search modes - Find all my search terms | 1,107 |
| S100 | S74 OR S75 OR S76 OR S77 OR S78 OR S79 OR S80 OR S81 OR S82 OR S83 OR S84 OR S85 OR S86 OR S87 OR S88 OR S89 OR S90 OR S91 OR S92 OR S93 OR S94 OR S95 OR S96 OR S97 OR S98 OR S99 | Expanders - Apply equivalent subjects  Search modes - Find all my search terms | 186,559 |
| S99 | TI ( (care or manag*) N2 themsel* ) OR AB ( (care or manag*) N2 themsel* ) | Expanders - Apply equivalent subjects  Search modes - Find all my search terms | 1,262 |
| S98 | TI ( (care or manag*) N2 herself ) OR AB ( (care or manag*) N2 herself ) | Expanders - Apply equivalent subjects  Search modes - Find all my search terms | 70 |
| S97 | TI ( (care or manag*) N2 himself ) OR AB ( (care or manag*) N2 himself ) | Expanders - Apply equivalent subjects  Search modes - Find all my search terms | 41 |
| S96 | TI ( (care or manag*) N2 self ) OR AB ( (care or manag*) N2 self ) | Expanders - Apply equivalent subjects  Search modes - Find all my search terms | 29,637 |
| S95 | MH "Self Care" | Expanders - Apply equivalent subjects  Search modes - Find all my search terms | 35,793 |
| S94 | MH "Self-Management" | Expanders - Apply equivalent subjects  Search modes - Find all my search terms | 625 |
| S93 | TI home or homes | Expanders - Apply equivalent subjects  Search modes - Find all my search terms | 60,354 |
| S92 | TI HBPR OR AB HBPR | Expanders - Apply equivalent subjects  Search modes - Find all my search terms | 1 |
| S91 | TI home# N2 PR OR AB home# N2 PR | Expanders - Apply equivalent subjects  Search modes - Find all my search terms | 24 |
| S90 | TI ( (patient* N0 home#) or (patient* N0 domicil*) ) OR AB ( (patient* N0 home#) or (patient* N0 domicil*) ) | Expanders - Apply equivalent subjects  Search modes - Find all my search terms | 2,325 |
| S89 | TI "in home" OR AB "in home" | Expanders - Apply equivalent subjects  Search modes - Find all my search terms | 118,250 |
| S88 | TI "at home" OR AB "at home" | Expanders - Apply equivalent subjects  Search modes - Find all my search terms | 118,250 |
| S87 | TI home N0 nurs* OR AB home N0 nurs* | Expanders - Apply equivalent subjects  Search modes - Find all my search terms | 19,804 |
| S86 | TI ( (domicil* or home#) N3 setting# ) OR AB ( (domicil* or home#) N3 setting# ) | Expanders - Apply equivalent subjects  Search modes - Find all my search terms | 5,386 |
| S85 | TI ( (domicil* or home#) N3 session# ) OR AB ( (domicil* or home#) N3 session# ) | Expanders - Apply equivalent subjects  Search modes - Find all my search terms | 489 |
| S84 | TI ( (domicil* or home#) N3 environment* ) OR AB ( (domicil* or home#) N3 environment* ) | Expanders - Apply equivalent subjects  Search modes - Find all my search terms | 4,356 |
| S83 | TI ( (domicil* or home#) N3 dwelling ) OR AB ( (domicil* or home#) N3 dwelling ) | Expanders - Apply equivalent subjects  Search modes - Find all my search terms | 555 |
| S82 | TI ( "home-based" or homebased or "domicile-based" ) OR AB ( "home-based" or homebased or "domicile-based" ) | Expanders - Apply equivalent subjects  Search modes - Find all my search terms | 6,256 |
| S81 | TI homecare OR AB homecare | Expanders - Apply equivalent subjects  Search modes - Find all my search terms | 921 |
| S80 | TI ( (domicil* or home#) N3 model* ) OR AB ( (domicil* or home#) N3 model* ) | Expanders - Apply equivalent subjects  Search modes - Find all my search terms | 1,688 |
| S79 | TI ( (domicil* or home#) N3 (assist* or manag* or monitor* or network* or program* or rehab* or resourc* or servic* or support*) ) OR AB ( (domicil* or home#) N3 (assist* or manag* or monitor* or network* or program* or rehab* or resourc* or servic* or support*) ) | Expanders - Apply equivalent subjects  Search modes - Find all my search terms | 21,687 |
| S78 | TI ( (domicil* or home#) N3 (care or "health care" or healthcare) ) OR AB ( (domicil* or home#) N3 (care or "health care" or healthcare) ) | Expanders - Apply equivalent subjects  Search modes - Find all my search terms | 30,998 |
| S77 | (MH "Home Respiratory Care+") | Expanders - Apply equivalent subjects  Search modes - Find all my search terms | 935 |
| S76 | (MH "Home Rehabilitation+") | Expanders - Apply equivalent subjects  Search modes - Find all my search terms | 1,922 |
| S75 | (MH "Home Nursing, Professional") | Expanders - Apply equivalent subjects  Search modes - Find all my search terms | 7,100 |
| S74 | (MH "Home Health Care") | Expanders - Apply equivalent subjects  Search modes - Find all my search terms | 22,126 |
| S73 | S19 AND S72 | Expanders - Apply equivalent subjects  Search modes - Find all my search terms | 10,003 |
| S72 | S51 OR S71 | Expanders - Apply equivalent subjects  Search modes - Find all my search terms | 93,379 |
| S71 | S52 OR S53 OR S54 OR S55 OR S56 OR S57 OR S58 OR S59 OR S60 OR S61 OR S62 OR S63 OR S64 OR S65 OR S66 OR S67 OR S68 OR S69 OR S70 | Expanders - Apply equivalent subjects  Search modes - Find all my search terms | 48,657 |
| S70 | TI CardioMEMS OR AB CardioMEMS | Expanders - Apply equivalent subjects  Search modes - Find all my search terms | 48 |
| S69 | ( (MH "Monitoring, Physiologic") AND (MH "Heart+") ) OR ( (MH "Monitoring, Physiologic") AND TI ((cardio* or cardiac* or coronar* or heart# or defibrillat* or fibrillat* or implant* ) ) OR ( (MH "Monitoring, Physiologic") AND AB ((cardio* or cardiac* or coronar* or heart# or defibrillat* or fibrillat* or implant* ) ) | Expanders - Apply equivalent subjects  Search modes - Find all my search terms | 4,115 |
| S68 | TI ( electrophysiolog* or (electro N0 physiolog*) ) OR AB ( electrophysiolog* or (electro N0 physiolog*) ) | Expanders - Apply equivalent subjects  Search modes - Find all my search terms | 11,213 |
| S67 | (MH "Electrophysiology") | Expanders - Apply equivalent subjects  Search modes - Find all my search terms | 7,859 |
| S66 | TI ( AICD or AICDs or "S-ICD" or "S-ICDs" or CIED or CIEDs ) OR AB ( AICD or AICDs or "S-ICD" or "S-ICDs" or CIED or CIEDs ) | Expanders - Apply equivalent subjects  Search modes - Find all my search terms | 769 |
| S65 | ( TI (ICD or ICDs) OR AB (ICD or ICDs) ) AND ( (MH "Lung+") OR AB ( cardio* or cardiac* or coronar* or heart# or defibrillat* or fibrillat* or implant* ) ) | Expanders - Apply equivalent subjects  Search modes - Find all my search terms | 3,655 |
| S64 | ( TI (ICD or ICDs) OR AB (ICD or ICDs) ) AND ( (MH "Lung+") OR TI ( cardio* or cardiac* or coronar* or heart# or defibrillat* or fibrillat* or implant* ) ) | Expanders - Apply equivalent subjects  Search modes - Find all my search terms | 3,255 |
| S63 | TI ( (cardiac* or cardio*) N3 implant* ) OR AB ( (cardiac* or cardio*) N3 implant* ) | Expanders - Apply equivalent subjects  Search modes - Find all my search terms | 7,729 |
| S62 | TI implant* N3 device* OR AB implant* N3 device* | Expanders - Apply equivalent subjects  Search modes - Find all my search terms | 5,564 |
| S61 | TI implant* N3 defibrillat* OR AB implant* N3 defibrillat* | Expanders - Apply equivalent subjects  Search modes - Find all my search terms | 6,869 |
| S60 | (MH "Defibrillators, Implantable") | Expanders - Apply equivalent subjects  Search modes - Find all my search terms | 9,351 |
| S59 | TI ( pacemak* or (pace N0 mak*) ) OR AB ( pacemak* or (pace N0 mak*) ) | Expanders - Apply equivalent subjects  Search modes - Find all my search terms | 8,097 |
| S58 | (MH "Pacemaker, Artificial") | Expanders - Apply equivalent subjects  Search modes - Find all my search terms | 7,296 |
| S57 | TI ( cardiac N0 (resynchroni* or (re N0 synchroni*)) ) OR AB ( cardiac N0 (resynchroni* or (re N0 synchroni*)) ) | Expanders - Apply equivalent subjects  Search modes - Find all my search terms | 3,851 |
| S56 | TI artificial* N2 card* N0 pac* OR AB artificial* N2 card* N0 pac* | Expanders - Apply equivalent subjects  Search modes - Find all my search terms | 15 |
| S55 | (MH "Cardiac Pacing, Artificial+") | Expanders - Apply equivalent subjects  Search modes - Find all my search terms | 8,238 |
| S54 | TI ( ((electronic N0 inhal*) N2 (monitoring N0 device#)) or EIMD or EIMDs ) OR AB ( ((electronic N0 inhal*) N2 (monitoring N0 device#)) or EIMD or EIMDs ) | Expanders - Apply equivalent subjects  Search modes - Find all my search terms | 77 |
| S53 | ( (MH "Monitoring, Physiologic") AND (MH "Lung+") ) OR ( (MH "Monitoring, Physiologic") AND TI ( breath* or lung* or pulmonar* or respirat* ) ) OR ( (MH "Monitoring, Physiologic") AND AB ( breath* or lung* or pulmonar* or respirat* ) ) | Expanders - Apply equivalent subjects  Search modes - Find all my search terms | 2,211 |
| S52 | TI "Smart Breath" N0 Analy?er# OR AB "Smart Breath" N0 Analy?er# | Expanders - Apply equivalent subjects  Search modes - Find all my search terms | 0 |
| S51 | S20 OR S21 OR S22 OR S23 OR S24 OR S25 OR S26 OR S27 OR S28 OR S29 OR S30 OR S31 OR S32 OR S33 OR S34 OR S35 OR S36 OR S37 OR S38 OR S39 OR S40 OR S41 OR S42 OR S43 OR S44 OR S45 OR S46 OR S47 OR S48 OR S49 OR S50 | Expanders - Apply equivalent subjects  Search modes - Find all my search terms | 47,158 |
| S50 | TI ( telemetr* or (tele N0 metr*) ) OR AB ( telemetr* or (tele N0 metr*) ) | Expanders - Apply equivalent subjects  Search modes - Find all my search terms | 1,560 |
| S49 | TI ( "remote sensing" or (remote N0 sensor*) ) OR AB ( "remote sensing" or (remote N0 sensor*) ) | Expanders - Apply equivalent subjects  Search modes - Find all my search terms | 143 |
| S48 | TI ( (internet* or app or apps or smarthome* or (smart N0 home*) or smartphone* or (smart N0 phone*) or "mobile-based" or (e N0 mail*) or email* or (electronic N0 mail*) or emedicine or (e N0 medicine) or technolog* or computer* or digital* or webbased or "web-based" or webdeliver* or (web N0 deliver*) or online) N3 (manag* or monitor*) ) OR AB ( (internet* or app or apps or smarthome* or (smart N0 home*) or smartphone* or (smart N0 phone*) or "mobile-based" or (e N0 mail*) or email* or (electronic N0 mail*) or emedicine or (e N0 medicine) or technolog* or computer* or digital* or webbased or "web-based" or webdeliver* or (web N0 deliver*) or online) N3 (manag* or monitor*) ) | Expanders - Apply equivalent subjects  Search modes - Find all my search terms | 7,934 |
| S47 | TI ( "mobile care" or mcare or "m-care" or "mobile health" or mhealth or "m-health" or "mobile healthcare" or mhealthcare or "m-healthcare" ) OR AB ( "mobile care" or mcare or "m-care" or "mobile health" or mhealth or "m-health" or "mobile healthcare" or mhealthcare or "m-healthcare" ) | Expanders - Apply equivalent subjects  Search modes - Find all my search terms | 2,596 |
| S46 | TI ( ecare or "e-care" or ehealth or "e-health" or ehealthcare or "e-healthcare" or emedicine or "e-medicine" ) OR AB ( ecare or "e-care" or ehealth or "e-health" or ehealthcare or "e-healthcare" or emedicine or "e-medicine" ) | Expanders - Apply equivalent subjects  Search modes - Find all my search terms | 3,271 |
| S45 | TI ( (dynamic* or Holter) N3 (manag* or monitor*) ) OR AB ( (dynamic* or Holter) N3 (manag* or monitor*) ) | Expanders - Apply equivalent subjects  Search modes - Find all my search terms | 1,850 |
| S44 | TI ( televisit* or (tele N0 visit*) or evisit* or (e N0 visit*) ) OR AB ( televisit* or (tele N0 visit*) or evisit* or (e N0 visit*) ) | Expanders - Apply equivalent subjects  Search modes - Find all my search terms | 136 |
| S43 | TI ( telesurveillance* or (tele N0 surveillance*) or esurveillance* or (e N0 surveillance*) ) OR AB ( telesurveillance* or (tele N0 surveillance*) or esurveillance* or (e N0 surveillance*) ) | Expanders - Apply equivalent subjects  Search modes - Find all my search terms | 17 |
| S42 | TI ( telerehab* or (tele N0 rehab*) or erehab* or (e N0 rehab*) ) OR AB ( telerehab* or (tele N0 rehab*) or erehab* or (e N0 rehab*) ) | Expanders - Apply equivalent subjects  Search modes - Find all my search terms | 477 |
| S41 | TI ( telemonitor* or (tele N0 monitor*) or emonitor* or (e N0 monitor*) ) OR AB ( telemonitor* or (tele N0 monitor*) or emonitor* or (e N0 monitor*) ) | Expanders - Apply equivalent subjects  Search modes - Find all my search terms | 941 |
| S40 | TI ( telemanag* or (tele N0 manag*) or emanag* or (e N0 manag*) ) OR AB ( telemanag* or (tele N0 manag*) or emanag* or (e N0 manag*) ) | Expanders - Apply equivalent subjects  Search modes - Find all my search terms | 236 |
| S39 | TI ( teleinterven* or (tele N0 interven*) or einterven* or (e N0 interven*) ) OR AB ( teleinterven* or (tele N0 interven*) or einterven* or (e N0 interven*) ) | Expanders - Apply equivalent subjects  Search modes - Find all my search terms | 647 |
| S38 | TI ( telecare or "tele-care" or telehealth or "tele-health" or telehealthcare or "tele-healthcare" or telemedicine or "tele-medicine" ) OR AB ( telecare or "tele-care" or telehealth or "tele-health" or telehealthcare or "tele-healthcare" or telemedicine or "tele-medicine" ) | Expanders - Apply equivalent subjects  Search modes - Find all my search terms | 7,963 |
| S37 | TI ( telefollowup# or (telefollow N0 up#) or (tele N0 followup#) or (tele N0 follow N0 up#) or efollowup# or (efollow N0 up#) or (e N0 followup#) or (e N0 follow N0 up#) ) OR AB ( telefollowup# or (telefollow N0 up#) or (tele N0 followup#) or (tele N0 follow N0 up#) or efollowup# or (efollow N0 up#) or (e N0 followup#) or (e N0 follow N0 up#) ) | Expanders - Apply equivalent subjects  Search modes - Find all my search terms | 12 |
| S36 | TI ( teleconsult* or (tele N0 consult*) or econsult* or (e N0 consult*) ) OR AB ( teleconsult* or (tele N0 consult*) or econsult* or (e N0 consult*) ) | Expanders - Apply equivalent subjects  Search modes - Find all my search terms | 681 |
| S35 | TI ( (remote* or virtual*) N3 visit* ) OR AB ( (remote* or virtual*) N3 visit* ) | Expanders - Apply equivalent subjects  Search modes - Find all my search terms | 229 |
| S34 | TI ( (remote* or virtual*) N3 surveillance* ) OR AB ( (remote* or virtual*) N3 surveillance* ) | Expanders - Apply equivalent subjects  Search modes - Find all my search terms | 48 |
| S33 | TI ( (remote* or virtual*) N3 rehab* ) OR AB ( (remote* or virtual*) N3 rehab* ) | Expanders - Apply equivalent subjects  Search modes - Find all my search terms | 380 |
| S32 | TI ( (outpatient* or "out-patient" or "out-patients" or ambulator* or home# or homebased or "home-based") N3 (manag* or monitor*) ) OR AB ( (outpatient* or "out-patient" or "out-patients" or ambulator* or home# or homebased or "home-based") N3 (manag* or monitor*) ) | Expanders - Apply equivalent subjects  Search modes - Find all my search terms | 10,291 |
| S31 | TI ( (remote* or virtual) N3 (monitor* or outpatient*) ) OR AB ( (remote* or virtual) N3 (monitor* or outpatient*) ) | Expanders - Apply equivalent subjects  Search modes - Find all my search terms | 1,321 |
| S30 | TI ( (remote* or virtual) N3 manag* ) OR AB ( (remote* or virtual) N3 manag* ) | Expanders - Apply equivalent subjects  Search modes - Find all my search terms | 460 |
| S29 | TI ( (remote* or virtual) N3 interven* ) OR AB ( (remote* or virtual) N3 interven* ) | Expanders - Apply equivalent subjects  Search modes - Find all my search terms | 477 |
| S28 | TI ( (remote* or virtual) N3 (followup# or follow-up#) ) OR AB ( (remote* or virtual) N3 (followup# or follow-up#) ) | Expanders - Apply equivalent subjects  Search modes - Find all my search terms | 125 |
| S27 | TI ( (remote* or virtual) N3 consult* ) OR AB ( (remote* or virtual) N3 consult* ) | Expanders - Apply equivalent subjects  Search modes - Find all my search terms | 276 |
| S26 | TI ( (remote* or virtual) N3 (care or healthcare or "health care") ) OR AB ( (remote* or virtual) N3 (care or healthcare or "health care") ) | Expanders - Apply equivalent subjects  Search modes - Find all my search terms | 1,277 |
| S25 | TI ( (remote* or virtual) N3 interrogat* ) OR AB ( (remote* or virtual) N3 interrogat* ) | Expanders - Apply equivalent subjects  Search modes - Find all my search terms | 20 |
| S24 | (MH "Telemetry") | Expanders - Apply equivalent subjects  Search modes - Find all my search terms | 1,874 |
| S23 | (MH "Telerehabilitation") | Expanders - Apply equivalent subjects  Search modes - Find all my search terms | 152 |
| S22 | (MH "Telemedicine") | Expanders - Apply equivalent subjects  Search modes - Find all my search terms | 10,163 |
| S21 | (MH "Remote Consultation") | Expanders - Apply equivalent subjects  Search modes - Find all my search terms | 1,800 |
| S20 | (MH "Electrocardiography, Ambulatory") | Expanders - Apply equivalent subjects  Search modes - Find all my search terms | 2,697 |
| S19 | S13 OR S18 | Expanders - Apply equivalent subjects  Search modes - Find all my search terms | 133,188 |
| S18 | S14 OR S15 OR S16 OR S17 | Expanders - Apply equivalent subjects  Search modes - Find all my search terms | 60,268 |
| S17 | TI ( ("cardio-renal" or cardiorenal or "reno-cardiac" or renocardiac) N1 syndrome# ) OR AB ( ("cardio-renal" or cardiorenal or "reno-cardiac" or renocardiac) N1 syndrome# ) | Expanders - Apply equivalent subjects  Search modes - Find all my search terms | 281 |
| S16 | TI ( (HF or CHF) and heart ) OR AB ( (HF or CHF) and heart ) | Expanders - Apply equivalent subjects  Search modes - Find all my search terms | 10,417 |
| S15 | TI ( (cardiac or cardial or heart or myocardia* or (myo N0 cardia*)) N1 ((backward N0 failure#) or decompensat* or failure# or incompetenc* or insufficienc* or standstill or "stand still") ) OR AB ( (cardiac or cardial or heart or myocardia* or (myo N0 cardia*)) N1 ((backward N0 failure#) or decompensat* or failure# or incompetenc* or insufficienc* or standstill or "stand still") ) | Expanders - Apply equivalent subjects  Search modes - Find all my search terms | 48,016 |
| S14 | (MH "Heart Failure+") | Expanders - Apply equivalent subjects  Search modes - Find all my search terms | 38,082 |
| S13 | S1 OR S2 OR S3 OR S4 OR S5 OR S6 OR S7 OR S8 OR S9 OR S10 OR S11 OR S12 | Expanders - Apply equivalent subjects  Search modes - Find all my search terms | 76,100 |
| S12 | TI ( (pulmonary or respirat*) N3 rehab* ) OR AB ( (pulmonary or respirat*) N3 rehab* ) | Expanders - Apply equivalent subjects  Search modes - Find all my search terms | 2,124 |
| S11 | MH "Rehabilitation, Pulmonary+" | Expanders - Apply equivalent subjects  Search modes - Find all my search terms | 2,673 |
| S10 | TI respirat* N0 therap* OR AB respirat* N0 therap* | Expanders - Apply equivalent subjects  Search modes - Find all my search terms | 2,474 |
| S9 | MH "Respiratory Therapy+" | Expanders - Apply equivalent subjects  Search modes - Find all my search terms | 44,105 |
| S8 | TI emphysema* OR AB emphysema* | Expanders - Apply equivalent subjects  Search modes - Find all my search terms | 3,849 |
| S7 | TI chronic N2 bronchitis OR AB chronic N2 bronchitis | Expanders - Apply equivalent subjects  Search modes - Find all my search terms | 1,023 |
| S6 | TI ( COPD or COAD ) OR AB ( COPD or COAD ) | Expanders - Apply equivalent subjects  Search modes - Find all my search terms | 14,370 |
| S5 | AB chronic N2 obstructi* AND AB ( pulmonary or airway* or (air N0 way*) or lung or lungs or airflow* or (air N0 flow*) ) | Expanders - Apply equivalent subjects  Search modes - Find all my search terms | 11,491 |
| S4 | TI chronic N2 obstructi* AND TI ( pulmonary or airway* or (air N0 way*) or lung or lungs or airflow* or (air N0 flow*) ) | Expanders - Apply equivalent subjects  Search modes - Find all my search terms | 7,066 |
| S3 | (MH "Emphysema") | Expanders - Apply equivalent subjects  Search modes - Find all my search terms | 2,580 |
| S2 | (MH "Pulmonary Disease, Chronic Obstructive+") | Expanders - Apply equivalent subjects  Search modes - Find all my search terms | 18,053 |
| S1 | (MH "Lung Diseases, Obstructive") | Expanders - Apply equivalent subjects  Search modes - Find all my search terms | 3,763 |

**EconLit**

| # | Query | Limiters/Expanders | Results |
| --- | --- | --- | --- |
| S32 | S10 AND S31 | Expanders - Apply equivalent subjects  Search modes - Find all my search terms | 6 |
| S31 | S11 OR S12 OR S13 OR S14 OR S15 OR S16 OR S17 OR S18 OR S19 OR S20 OR S21 OR S22 OR S23 OR S24 OR S25 OR S26 OR S27 OR S28 OR S29 OR S30 | Expanders - Apply equivalent subjects  Search modes - Find all my search terms | 20,851 |
| S30 | TI ( (care or manag*) N2 themsel* ) OR AB ( (care or manag*) N2 themsel* ) | Expanders - Apply equivalent subjects  Search modes - Find all my search terms | 147 |
| S29 | TI ( (care or manag*) N2 herself ) OR AB ( (care or manag*) N2 herself ) | Expanders - Apply equivalent subjects  Search modes - Find all my search terms | 2 |
| S28 | TI ( (care or manag*) N2 himself ) OR AB ( (care or manag*) N2 himself ) | Expanders - Apply equivalent subjects  Search modes - Find all my search terms | 7 |
| S27 | TI ( (care or manag*) N2 self ) OR AB ( (care or manag*) N2 self ) | Expanders - Apply equivalent subjects  Search modes - Find all my search terms | 890 |
| S26 | TI home or homes | Expanders - Apply equivalent subjects  Search modes - Find all my search terms | 19,780 |
| S25 | TI HBPR OR AB HBPR | Expanders - Apply equivalent subjects  Search modes - Find all my search terms | 0 |
| S24 | TI home# N2 PR OR AB home# N2 PR | Expanders - Apply equivalent subjects  Search modes - Find all my search terms | 0 |
| S23 | TI ( (patient* N0 home#) or (patient* N0 domicil*) ) OR AB ( (patient* N0 home#) or (patient* N0 domicil*) ) | Expanders - Apply equivalent subjects  Search modes - Find all my search terms | 8 |
| S22 | TI "in home" OR AB "in home" | Expanders - Apply equivalent subjects  Search modes - Find all my search terms | 17,393 |
| S21 | TI "at home" OR AB "at home" | Expanders - Apply equivalent subjects  Search modes - Find all my search terms | 17,393 |
| S20 | TI home N0 nurs* OR AB home N0 nurs* | Expanders - Apply equivalent subjects  Search modes - Find all my search terms | 590 |
| S19 | TI ( (domicil* or home#) N3 setting# ) OR AB ( (domicil* or home#) N3 setting# ) | Expanders - Apply equivalent subjects  Search modes - Find all my search terms | 65 |
| S18 | TI ( (domicil* or home#) N3 session# ) OR AB ( (domicil* or home#) N3 session# ) | Expanders - Apply equivalent subjects  Search modes - Find all my search terms | 1 |
| S17 | TI ( (domicil* or home#) N3 environment* ) OR AB ( (domicil* or home#) N3 environment* ) | Expanders - Apply equivalent subjects  Search modes - Find all my search terms | 263 |
| S16 | TI ( (domicil* or home#) N3 dwelling ) OR AB ( (domicil* or home#) N3 dwelling ) | Expanders - Apply equivalent subjects  Search modes - Find all my search terms | 21 |
| S15 | TI ( "home-based" or homebased or "domicile-based" ) OR AB ( "home-based" or homebased or "domicile-based" ) | Expanders - Apply equivalent subjects  Search modes - Find all my search terms | 325 |
| S14 | TI homecare OR AB homecare | Expanders - Apply equivalent subjects  Search modes - Find all my search terms | 23 |
| S13 | TI ( (domicil* or home#) N3 model* ) OR AB ( (domicil* or home#) N3 model* ) | Expanders - Apply equivalent subjects  Search modes - Find all my search terms | 434 |
| S12 | TI ( (domicil* or home#) N3 (assist* or manag* or monitor* or network* or program* or rehab* or resourc* or servic* or support*) ) OR AB ( (domicil* or home#) N3 (assist* or manag* or monitor* or network* or program* or rehab* or resourc* or servic* or support*) ) | Expanders - Apply equivalent subjects  Search modes - Find all my search terms | 984 |
| S11 | TI ( (domicil* or home#) N3 (care or "health care" or healthcare) ) OR AB ( (domicil* or home#) N3 (care or "health care" or healthcare) ) | Expanders - Apply equivalent subjects  Search modes - Find all my search terms | 745 |
| S10 | S1 OR S2 OR S3 OR S4 OR S5 OR S6 OR S7 OR S8 OR S9 | Expanders - Apply equivalent subjects  Search modes - Find all my search terms | 198 |
| S9 | TI ( ("cardio-renal" or cardiorenal or "reno-cardiac" or renocardiac) N1 syndrome# ) OR AB ( ("cardio-renal" or cardiorenal or "reno-cardiac" or renocardiac) N1 syndrome# ) | Expanders - Apply equivalent subjects  Search modes - Find all my search terms | 0 |
| S8 | TI ( (HF or CHF) and heart ) OR AB ( (HF or CHF) and heart ) | Expanders - Apply equivalent subjects  Search modes - Find all my search terms | 22 |
| S7 | TI ( (cardiac or cardial or heart or myocardia* or (myo N0 cardia*)) N1 ((backward N0 failure#) or decompensat* or failure# or incompetenc* or insufficienc* or standstill or "stand still") ) OR AB ( (cardiac or cardial or heart or myocardia* or (myo N0 cardia*)) N1 ((backward N0 failure#) or decompensat* or failure# or incompetenc* or insufficienc* or standstill or "stand still") ) | Expanders - Apply equivalent subjects  Search modes - Find all my search terms | 123 |
| S6 | TI ( (pulmonary or respirat*) N3 rehab* ) OR AB ( (pulmonary or respirat*) N3 rehab* ) | Expanders - Apply equivalent subjects  Search modes - Find all my search terms | 3 |
| S5 | TI respirat* N0 therap* OR AB respirat* N0 therap* | Expanders - Apply equivalent subjects  Search modes - Find all my search terms | 1 |
| S4 | TI chronic N2 bronchitis OR AB chronic N2 bronchitis | Expanders - Apply equivalent subjects  Search modes - Find all my search terms | 15 |
| S3 | TI ( COPD or COAD ) OR AB ( COPD or COAD ) | Expanders - Apply equivalent subjects  Search modes - Find all my search terms | 40 |
| S2 | AB chronic N2 obstructi* AND AB ( pulmonary or airway* or (air N0 way*) or lung or lungs or airflow* or (air N0 flow*) ) | Expanders - Apply equivalent subjects  Search modes - Find all my search terms | 42 |
| S1 | TI chronic N2 obstructi* AND TI ( pulmonary or airway* or (air N0 way*) or lung or lungs or airflow* or (air N0 flow*) ) | Expanders - Apply equivalent subjects  Search modes - Find all my search terms | 18 |

**Web of Science**

|  |  |  |
| --- | --- | --- |
| # 21 | [1,732](http://apps.webofknowledge.com.login.ezproxy.library.ualberta.ca/summary.do?product=WOS&doc=1&qid=30&SID=5Fd3oPAXCeTmM1N8ZYv&search_mode=CombineSearches&update_back2search_link_param=yes) | #19 AND #11  **Refined by:** **LANGUAGES:** ( ENGLISH )  Indexes=SCI-EXPANDED, SSCI, A&HCI, CPCI-S, CPCI-SSH, BKCI-S, BKCI-SSH, ESCI, CCR-EXPANDED, IC Timespan=All years |
| # 20 | [1,785](http://apps.webofknowledge.com.login.ezproxy.library.ualberta.ca/summary.do?product=WOS&doc=1&qid=29&SID=5Fd3oPAXCeTmM1N8ZYv&search_mode=CombineSearches&update_back2search_link_param=yes) | #19 AND #11  Indexes=SCI-EXPANDED, SSCI, A&HCI, CPCI-S, CPCI-SSH, BKCI-S, BKCI-SSH, ESCI, CCR-EXPANDED, IC Timespan=All years |
| # 19 | [212,327](http://apps.webofknowledge.com.login.ezproxy.library.ualberta.ca/summary.do?product=WOS&doc=1&qid=28&SID=5Fd3oPAXCeTmM1N8ZYv&search_mode=CombineSearches&update_back2search_link_param=yes) | #18 OR #17 OR #16 OR #15 OR #14 OR #13 OR #12  Indexes=SCI-EXPANDED, SSCI, A&HCI, CPCI-S, CPCI-SSH, BKCI-S, BKCI-SSH, ESCI, CCR-EXPANDED, IC Timespan=All years |
| # 18 | [56,536](http://apps.webofknowledge.com.login.ezproxy.library.ualberta.ca/summary.do?product=WOS&doc=1&qid=27&SID=5Fd3oPAXCeTmM1N8ZYv&search_mode=GeneralSearch&update_back2search_link_param=yes) | **TOPIC:** ((care or manag*) NEAR/2 self) *OR* **TOPIC:** ((care or manag*) NEAR/2 himself) *OR* **TOPIC:** ((care or manag*) NEAR/2 herself) *OR* **TOPIC:** ((care or manag*) NEAR/2 themsel*)  Indexes=SCI-EXPANDED, SSCI, A&HCI, CPCI-S, CPCI-SSH, BKCI-S, BKCI-SSH, ESCI, CCR-EXPANDED, IC Timespan=All years |
| # 17 | [17,507](http://apps.webofknowledge.com.login.ezproxy.library.ualberta.ca/summary.do?product=WOS&doc=1&qid=26&SID=5Fd3oPAXCeTmM1N8ZYv&search_mode=GeneralSearch&update_back2search_link_param=yes) | **TOPIC:** (patient* NEAR/2 (home or homes or domicil*)) *OR* **TOPIC:** (home* NEAR/2 PR) *OR* **TOPIC:** (HBPR)  Indexes=SCI-EXPANDED, SSCI, A&HCI, CPCI-S, CPCI-SSH, BKCI-S, BKCI-SSH, ESCI, CCR-EXPANDED, IC Timespan=All years |
| # 16 | [72,687](http://apps.webofknowledge.com.login.ezproxy.library.ualberta.ca/summary.do?product=WOS&doc=1&qid=25&SID=5Fd3oPAXCeTmM1N8ZYv&search_mode=GeneralSearch&update_back2search_link_param=yes) | **TOPIC:** ("at home") *OR* **TOPIC:** ("in home")  Indexes=SCI-EXPANDED, SSCI, A&HCI, CPCI-S, CPCI-SSH, BKCI-S, BKCI-SSH, ESCI, CCR-EXPANDED, IC Timespan=All years |
| # 15 | [946](http://apps.webofknowledge.com.login.ezproxy.library.ualberta.ca/summary.do?product=WOS&doc=1&qid=24&SID=5Fd3oPAXCeTmM1N8ZYv&search_mode=GeneralSearch&update_back2search_link_param=yes) | **TOPIC:** ("home nursing" OR "home nurse" OR "home nursed" OR "home nurses")  Indexes=SCI-EXPANDED, SSCI, A&HCI, CPCI-S, CPCI-SSH, BKCI-S, BKCI-SSH, ESCI, CCR-EXPANDED, IC Timespan=All years |
| # 14 | [22,772](http://apps.webofknowledge.com.login.ezproxy.library.ualberta.ca/summary.do?product=WOS&doc=1&qid=23&SID=5Fd3oPAXCeTmM1N8ZYv&search_mode=GeneralSearch&update_back2search_link_param=yes) | **TOPIC:** ((domicil* or home or homes) NEAR/3 dwelling) *OR* **TOPIC:** ((domicil* or home or homes) NEAR/3 environment*) *OR* **TOPIC:** ((domicil* or home or homes) NEAR/3 session*) *OR* **TOPIC:** ((domicil* or home or homes) NEAR/3 setting*)  Indexes=SCI-EXPANDED, SSCI, A&HCI, CPCI-S, CPCI-SSH, BKCI-S, BKCI-SSH, ESCI, CCR-EXPANDED, IC Timespan=All years |
| # 13 | [14,515](http://apps.webofknowledge.com.login.ezproxy.library.ualberta.ca/summary.do?product=WOS&doc=1&qid=22&SID=5Fd3oPAXCeTmM1N8ZYv&search_mode=GeneralSearch&update_back2search_link_param=yes) | **TOPIC:** (homecare) *OR* **TOPIC:** ("home-based" or homebased or "domicile-based")  Indexes=SCI-EXPANDED, SSCI, A&HCI, CPCI-S, CPCI-SSH, BKCI-S, BKCI-SSH, ESCI, CCR-EXPANDED, IC Timespan=All years |
| # 12 | [73,392](http://apps.webofknowledge.com.login.ezproxy.library.ualberta.ca/summary.do?product=WOS&doc=1&qid=21&SID=5Fd3oPAXCeTmM1N8ZYv&search_mode=GeneralSearch&update_back2search_link_param=yes) | **TOPIC:** ((domicil* or home or homes) NEAR/3 (care or "health care" or healthcare)) *OR* **TOPIC:** ((domicil* or home or homes) NEAR/3 (assist* or manag* or monitor* or network* or program* or rehab* or resourc* or servic* or support*)) *OR* **TOPIC:** ((domicil* or home or homes) NEAR/3 model*)  Indexes=SCI-EXPANDED, SSCI, A&HCI, CPCI-S, CPCI-SSH, BKCI-S, BKCI-SSH, ESCI, CCR-EXPANDED, IC Timespan=All years |
| # 11 | [25,287](http://apps.webofknowledge.com.login.ezproxy.library.ualberta.ca/summary.do?product=WOS&doc=1&qid=20&SID=5Fd3oPAXCeTmM1N8ZYv&search_mode=CombineSearches&update_back2search_link_param=yes) | #10 AND #4  Indexes=SCI-EXPANDED, SSCI, A&HCI, CPCI-S, CPCI-SSH, BKCI-S, BKCI-SSH, ESCI, CCR-EXPANDED, IC Timespan=All years |
| # 10 | [555,929](http://apps.webofknowledge.com.login.ezproxy.library.ualberta.ca/summary.do?product=WOS&doc=1&qid=19&SID=5Fd3oPAXCeTmM1N8ZYv&search_mode=CombineSearches&update_back2search_link_param=yes) | #9 OR #8 OR #7 OR #6 OR #5  Indexes=SCI-EXPANDED, SSCI, A&HCI, CPCI-S, CPCI-SSH, BKCI-S, BKCI-SSH, ESCI, CCR-EXPANDED, IC Timespan=All years |
| # 9 | [206,122](http://apps.webofknowledge.com.login.ezproxy.library.ualberta.ca/summary.do?product=WOS&doc=1&qid=18&SID=5Fd3oPAXCeTmM1N8ZYv&search_mode=GeneralSearch&update_back2search_link_param=yes) | **TOPIC:** ("Smart Breath Analyser" or "Smart Breath Analysers" or "Smart Breath Analyzer" or "Smart Breath Analyzers") *OR* **TOPIC:** (((electronic NEAR/0 inhal*) NEAR/2 (monitoring NEAR/0 device*)) or EIMD or EIMDs) *OR* **TOPIC:** (artificial* NEAR/2 (card* NEAR/0 pac*)) *OR* **TOPIC:** (cardiac NEAR/0 (resynchroni* or re-synchroni*)) *OR* **TOPIC:** (pacemak* or (pace NEAR/0 mak*)) *OR* **TOPIC:** (implant* NEAR/3 (defibrillat* or device*)) *OR* **TOPIC:** ((cardiac* or cardio*) NEAR/3 implant*) *OR* **TOPIC:** ((ICD or ICDs) AND (cardio* or cardiac* or coronar* or heart or hearts or defibrillat* or fibrillat* or implant*)) *OR* **TOPIC:** (AICD or AICDs or S-ICD or S-ICDs or CIED or CIEDs) *OR* **TOPIC:** (electrophysiolog* or (electro NEAR/0 physiolog*)) *OR* **TOPIC:** (CardioMEMS)  Indexes=SCI-EXPANDED, SSCI, A&HCI, CPCI-S, CPCI-SSH, BKCI-S, BKCI-SSH, ESCI, CCR-EXPANDED, IC Timespan=All years |
| # 8 | [83,069](http://apps.webofknowledge.com.login.ezproxy.library.ualberta.ca/summary.do?product=WOS&doc=1&qid=15&SID=5Fd3oPAXCeTmM1N8ZYv&search_mode=GeneralSearch&update_back2search_link_param=yes) | **TOPIC:** ((internet* or app or apps or smarthome* or (smart NEAR/0 home*) or smartphone* or (smart NEAR/0 phone*) or "mobile-based" or (e NEAR/0 mail*) or email* or (electronic NEAR/0 mail*) or emedicine or "e-medicine" or technolog* or computer* or digital* or webbased or "web-based" or webdeliver* or (web NEAR/0 deliver*) or online) NEAR/3 manag*) *OR* **TOPIC:** ((internet* or app or apps or smarthome* or (smart NEAR/0 home*) or smartphone* or (smart NEAR/0 phone*) or "mobile-based" or (e NEAR/0 mail*) or email* or (electronic NEAR/0 mail*) or emedicine or "e-medicine" or technolog* or computer* or digital* or webbased or "web-based" or webdeliver* or (web NEAR/0 deliver*) or online) NEAR/3 monitor*)  Indexes=SCI-EXPANDED, SSCI, A&HCI, CPCI-S, CPCI-SSH, BKCI-S, BKCI-SSH, ESCI, CCR-EXPANDED, IC Timespan=All years |
| # 7 | [195,990](http://apps.webofknowledge.com.login.ezproxy.library.ualberta.ca/summary.do?product=WOS&doc=1&qid=12&SID=5Fd3oPAXCeTmM1N8ZYv&search_mode=GeneralSearch&update_back2search_link_param=yes) | **TOPIC:** ((dynamic* or Holter) NEAR/3 (manag* or monitor*)) *OR* **TOPIC:** (ecare or "e-care" or ehealth or "e-health" or ehealthcare or "e-healthcare" or emedicine or "e-medicine") *OR* **TOPIC:** ("mobile care" or mcare or "m-care" or "mobile health" or mhealth or "m-health" or "mobile healthcare" or mhealthcare or "m-healthcare") *OR* **TOPIC:** ("remote sensing" or (remote NEAR/0 sensor*)) *OR* **TOPIC:** (telemetr* or (tele NEAR/0 metr*))  Indexes=SCI-EXPANDED, SSCI, A&HCI, CPCI-S, CPCI-SSH, BKCI-S, BKCI-SSH, ESCI, CCR-EXPANDED, IC Timespan=All years |
| # 6 | [32,999](http://apps.webofknowledge.com.login.ezproxy.library.ualberta.ca/summary.do?product=WOS&doc=1&qid=11&SID=5Fd3oPAXCeTmM1N8ZYv&search_mode=GeneralSearch&update_back2search_link_param=yes) | **TOPIC:** (teleconsult* or (tele NEAR/0 consult*) or econsult* or (e NEAR/0 consult*)) *OR* **TOPIC:** (telefollowup* or "telefollow-up" or "tele-followup" or "tele-follow-up" or efollowup* or "efollow-up" or "e-followup" or "e-follow-up") *OR* **TOPIC:** (telecare or "tele-care" or telehealth or "tele-health" or "telehealthcare" or "tele-healthcare" or telemedicine or "tele-medicine") *OR* **TOPIC:** (teleinterven* or (tele NEAR/0 interven*) or einterven* or (e NEAR/0 interven*)) *OR* **TOPIC:** (telemanag* or (tele NEAR/0 manag*) or emanag* or (e NEAR/0 manag*)) *OR* **TOPIC:** (telemonitor* or (tele NEAR/0 monitor*) or emonitor* or (e NEAR/0 monitor*)) *OR* **TOPIC:** (telerehab* or (tele NEAR/0 rehab*) or erehab* or (e NEAR/0 rehab*)) *OR* **TOPIC:** (telesurveillance* or (tele NEAR/0 surveillance*) or esurveillance* or (e NEAR/0 surveillance*)) *OR* **TOPIC:** (televisit* or (tele NEAR/0 visit*) or evisit* or (e NEAR/0 visit*))  Indexes=SCI-EXPANDED, SSCI, A&HCI, CPCI-S, CPCI-SSH, BKCI-S, BKCI-SSH, ESCI, CCR-EXPANDED, IC Timespan=All years |
| # 5 | [63,228](http://apps.webofknowledge.com.login.ezproxy.library.ualberta.ca/summary.do?product=WOS&doc=1&qid=10&SID=5Fd3oPAXCeTmM1N8ZYv&search_mode=GeneralSearch&update_back2search_link_param=yes) | **TOPIC:** ((remote* or virtual) NEAR/3 (interrogat* or care or healthcare or "health care" or consult* or followup* or (follow NEAR/0 up) or (follow NEAR/0 ups) or interven* or manag* or monitor* or outpatient* or rehab* or surveillance* or visit*)) *OR* **TOPIC:** ((outpatient* or (out NEAR/0 patient*) or ambulator* or home or homes or homebased or "home-based") NEAR/3 (manag* or monitor*))  Indexes=SCI-EXPANDED, SSCI, A&HCI, CPCI-S, CPCI-SSH, BKCI-S, BKCI-SSH, ESCI, CCR-EXPANDED, IC Timespan=All years |
| # 4 | [366,514](http://apps.webofknowledge.com.login.ezproxy.library.ualberta.ca/summary.do?product=WOS&doc=1&qid=9&SID=5Fd3oPAXCeTmM1N8ZYv&search_mode=CombineSearches&update_back2search_link_param=yes) | #3 OR #2 OR #1  Indexes=SCI-EXPANDED, SSCI, A&HCI, CPCI-S, CPCI-SSH, BKCI-S, BKCI-SSH, ESCI, CCR-EXPANDED, IC Timespan=All years |
| # 3 | [258,642](http://apps.webofknowledge.com.login.ezproxy.library.ualberta.ca/summary.do?product=WOS&doc=1&qid=6&SID=5Fd3oPAXCeTmM1N8ZYv&search_mode=GeneralSearch&update_back2search_link_param=yes) | **TOPIC:** ((cardiac or cardial or heart or myocardia* or (myo NEAR/0 cardia*)) NEAR/1 ("backward failure" or "backward failures" or decompensat* or failure or failures or incompetenc* or insufficienc* or standstill or "stand still")) *OR* **TOPIC:** ((HF or CHF) and heart) *OR* **TOPIC:** (("cardio-renal" or cardiorenal or "reno-cardiac" or renocardiac) NEAR/1 syndrome*)  Indexes=SCI-EXPANDED, SSCI, A&HCI, CPCI-S, CPCI-SSH, BKCI-S, BKCI-SSH, ESCI, CCR-EXPANDED, IC Timespan=All years |
| # 2 | [93,143](http://apps.webofknowledge.com.login.ezproxy.library.ualberta.ca/summary.do?product=WOS&doc=1&qid=4&SID=5Fd3oPAXCeTmM1N8ZYv&search_mode=GeneralSearch&update_back2search_link_param=yes) | **TOPIC:** (COPD or COAD) *OR* **TOPIC:** (chronic NEAR/2 bronchitis) *OR* **TOPIC:** (emphysema*) *OR* **TOPIC:** (respirat* NEAR/0 therap*) *OR* **TOPIC:** ((pulmonary or respirat*) NEAR/3 rehab*)  Indexes=SCI-EXPANDED, SSCI, A&HCI, CPCI-S, CPCI-SSH, BKCI-S, BKCI-SSH, ESCI, CCR-EXPANDED, IC Timespan=All years |
| # 1 | [53,248](http://apps.webofknowledge.com.login.ezproxy.library.ualberta.ca/summary.do?product=WOS&doc=1&qid=3&SID=5Fd3oPAXCeTmM1N8ZYv&search_mode=GeneralSearch&update_back2search_link_param=yes) | **TOPIC:** ((chronic NEAR/2 obstructi*)) *AND* **TOPIC:** (pulmonary or airway* or (air NEAR/0 way*) or lung or lungs or airflow* or (air NEAR/0 flow*))  Indexes=SCI-EXPANDED, SSCI, A&HCI, CPCI-S, CPCI-SSH, BKCI-S, BKCI-SSH, ESCI, CCR-EXPANDED, IC Timespan=All years |

| Table S2. Characteristics of included studies | | | | | | | |
| --- | --- | --- | --- | --- | --- | --- | --- |
| **Study**  **(country)** | **Study period**  **(Design)** | **Study objective** | **Eligibility criteria** | **Number of centres** | **Number of participants** | **Follow-up** | **Outcomes** |
| ***RHM (smartphones, apps, tablets) vs no RHM*** | | | | | | | |
| Park 2020(25)  (South Korea) | Mar 2016- Jun 2018  (RCT) | To examine the effect of a smartphone app-based, self-management program on self-care behavior | *Inclusion criteria:*   - Age≥ 45 years old - Mild, moderate or severe COPD - Had a smartphone and could text messages - Able to communicate   *Exclusion criteria:*   - Psychiatric disorder - COPD-related hospitalization in the last 2 months - Exacerbation - Oxygen saturation <93% in a stable state or <85% after a six minute walk test - Severe respiratory symptoms in a stable state - Attended PR in the previous year - Other diseases that made physical activity and/or exercise difficult - Use of assistive devices to walk or problems with balance | Multiple centres | RHM: 23  no RHM: 21 | 6 months | - Adherence - ER visits - Exacerbations - Exercise capacity and activity levels - Health-related quality of life - Hospital admissions - Lung function and other symptoms - Mental health - Patient experience - Safety - Self-efficacy - Visits to physician |
| Boer 2019(26)  (Netherlands) | Jun 2015- Jul 2016  (RCT) | To examine the effects of a smart mobile health (mHealth) tool that supports COPD patients in the self-management of exacerbations | *Inclusion criteria:*   - Age ≥40 years old - Spirometry-confirmed diagnosis of COPD (FEV_1_/FEVC<70%) - 2 or more exacerbations in the last year - Had experienced 2 or more symptom-based exacerbations   *Exclusion criteria:*   - Severe comorbid conditions that prohibited safe participation - Insufficient knowledge of the Dutch language - Persisting difficulties in using the mHealth system after a 2-week practice period and additional assistance | Multiple centres | RHM: 43  no RHM: 44 | 12 months | - Adherence - Exacerbations - Health-related quality of life - Hospital admissions - Lung function and other symptoms - Mortality - Patient experience - Self-efficacy - Visits to physician |
| Walker 2018 (27)  (Spain, UK, Slovenia, Estonia, Sweden) | Oct 2013- Apr 2016  (RCT) | To evaluate the effectiveness of remote monitoring in reducing healthcare utilization | *Inclusion criteria:*   - Age ≥60 years old - Moderate to very severe diagnosis of COPD - Acute exacerbation with or without hospitalization in the previous year - Smoking history of ≥10 pack-years - One or more chronic conditions (congestive heart failure, ischemic heart disease, hypertension, hyperlipidemia and clinically signiﬁcant sleep-disordered breathing) - Clinically stable, with at least 4 weeks since the last exacerbation   *Exclusion criteria:*   - Signiﬁcant visual disturbance or mental health disorders - Planned prolonged absence from home - Living in areas not covered by a mobile data network - Unable to use the study equipment | Multiple centres | RHM: 154  no RHM: 158 | 9 months | - Adherence - Cost - Exacerbations - Health-related quality of life - Hospital admissions - Length of hospitalization - Mental health - Mortality |
| Tabak 2014a(28)  (Netherlands) | Dec 2011- Jul 2013  (RCT) | To investigate the satisfaction and use of telehealth in patients with COPD | *Inclusion criteria:*   - Age> 40 years old - Diagnosis of COPD based on the GOLD criteria - Internet access at home. - Able to understand Dutch   *Exclusion criteria:*   - Age <75 years old - Exacerbation in the previous month - Three or more exacerbations in the previous two years - One hospitalization for respiratory problems in the previous two years - Serious disease with low survival rates - Other diseases influencing bronchial symptoms and/or lung function (e.g., cardiac insufficiency, sarcoidosis) - Severe psychiatric illness - Uncontrolled diabetes mellitus | Multiple centres | RHM: 15  no RHM: 14 | 9 months | - Adherence - ER visits - Exacerbations - Exercise capacity and activity levels - Health-related quality of life - Hospital admissions - Length of hospitalization - Lung function and other symptoms - Patient experience |
| Tabak 2014b (29)  (Netherlands) | Oct 2010- Apr 2011  (RCT) | To assess the effectiveness of telerehabilitation in patients with COPD | *Inclusion criteria:*   - Current or former smoker - Able to read and speak Dutch - Internet access at home   *Exclusion criteria:*   - Infection or exacerbation in the previous month - Impaired hand function causing inability to use the intervention - Disorders or progressive disease seriously influencing daily activities (e.g. amputation) - Other diseases influencing bronchial symptoms and/or lung function (e.g. sarcoidosis) - Regular oxygen therapy (>16 hours per day or pO2 < 7.2 kPa) - Asthma - Attended physiotherapy in the last six weeks | NR | RHM: 18  no RHM: 16 | 1 month | - Adherence - Exercise capacity and activity levels - Health-related quality of life - Lung function and other symptoms |
| ***RHM (dedicated monitoring devices) vs no RHM*** | | | | | | | |
| Shany 2017(30)  (Australia) | Mar 2009- Oct 2010  (RCT) | To investigate the effects of home tele monitoring in patients with severe COPD | *Inclusion criteria:*   - At least one hospital admission for an exacerbation in the last year   *Exclusion criteria:*   - Insufficient English fluency - Motor deficits that might prevent the use of the telehealth - Cognitive impairment - Participation in another trial - No landline phone connection at home | Single centre | RHM: 21  no RHM: 21 | 12 months | - Adherence - Cost - ER visits - Hospital admissions - Length of hospitalization - Mental health - Mortality - Patient experience - Provider experience |
| Vianello 2016 (31)  (Italy) | Nov 2011- May 2014  (RCT) | To investigate the benefits of a telemonitoring system in managing acute exacerbation advanced-stage COPD patients | *Inclusion criteria:*   - Age ≥18 years old - Severe to very severe diagnosis of COPD - Life expectancy >12 months - Capability of using, alone or assisted, the intervention   *Exclusion criteria:*   - Concomitant significant lung disease - Negative advice of the GP - Serious social problems, including lack of adequate family support and/or other social support networks | Multiple centres | RHM: 230  no RHM: 104 | 12 months | - Adherence - ER visits - Health-related quality of life - Hospital admissions - Length of hospitalization - Mental health - Mortality - Visits to physician |
| Segrelles 2014(32)  (Spain) | Jan 2010- Jul 2011  (RCT) | To assess the efficacy and effectiveness of a home telehealth program for COPD patients with severe airflow obstruction | *Inclusion criteria:*   - Age ≥ 50 years old - Severe to very severe diagnosis of COPD (FEV_1_< 50% predicted, FEV_1_/FVC ratio< 70%) - Long-term home oxygen therapy   *Exclusion criteria:*   - Current smoker - Enrolled in a palliative care program - Institutionalized or at risk of social exclusion - Unable to understand all procedures | Multiple centres | RHM: 30  no RHM: 30 | 7 months | - Adherence - ER visits - Hospital admissions - Length of hospitalization - Mortality |
| De San Miguel 2013(33)  (Australia) | NR  (RCT) | To understand the impact of telehealth monitoring for COPD patients on health service utilization and cost-effectiveness | *Inclusion criteria:*   - Diagnosis of COPD - Use of home oxygen therapy - Able to speak English   *Exclusion criteria:*   - Dementia - Palliative care - No telephone landline - Unable to use telehealth equipment due to cognitive impairment or physical disability | Single centre | RHM: 40  no RHM: 40 | 6 months | - Cost - ER visits - Health-related quality of life - Hospital admission - Length of hospitalization - Patient experience - Visits to physician |
| Jehn 2013(34)  (Germany) | Jan 2012- Jan 2013  (RCT) | To determine if the use of home monitoring reduces risk of exacerbations due to changes in the weather | *Inclusion criteria:*   - Age≥ 40 years old - Moderate to very severe diagnosis of COPD (FEV_1_ < 80% predicted and FEV_1_/FVC ratio <70%) - At least one exacerbation in the previous year - Clinically stable for the last month   *Exclusion criteria:*   - Asthma - Long-term oxygen therapy - Severe heart, liver or kidney disease - Any end stage malignant disease with life expectancy of less than six months - Listed for a lung transplant - Severe depression - Residents in nursing home - Physical disabilities limiting them from performing six minute walk tests - Mentally disabled | Single centre | RHM: 32  no RHM: 30 | 9 months | - Adherence - Exacerbations - Exercise capacity and activity levels - Health-related quality of life - Hospital admissions - Length of hospitalization - Lung function and other symptoms - Visits to physician |
| Jodar-Sanchez 2013(35)  (Spain) | Sep 2010- May 2011  (RCT) | To analyze the effectiveness of a telehealth programme in patients with advanced COPD | Inclusion criteria:   - Adult - Diagnosis of COPD - Long-term oxygen therapy - At least one hospitalisation for respiratory illness in the previous year - Clinically stable during the previous three months   *Exclusion criteria:*   - No home telephone line | Single centre | RHM: 24  no RHM: 21 | 4 months | - Adherence - ER visits - Health-related quality of life - Hospital admissions - Length of hospitalization - Mortality - Patient experience - Provider experience - Visits to physician |
| Pare 2013(36)  (Canada) | Sep 2010- Oct 2011  (RCT) | To assess the effectiveness of home monitoring in reducing costs associated with managing COPD | *Inclusion criteria:*   - Very serious COPD requiring frequent home visits (FEV_1_ <45% predicted) - At least one hospitalization in the previous year - Willingness to manage their health status (with or without an informal caregiver) - Able to communicate in English or French - An operational telephone line at the home   *Exclusion criteria:*   - Suffered from psychological or psychiatric problems - Cognitive deficit - Visual or motor deficit that would unable the use of the intervention unless an informal caregiver agreed to assist | Multiple centres | RHM: 60  no RHM: 60 | Pre-phase: 12 months  Post phase: 12 months | - Cost - ER visits - Hospital admissions - Length of hospitalization - Patient experience |
| Chau 2012(37)  (Hong Kong) | 2010- NR  (RCT) | To examine user satisfaction and effectiveness of telecare services in patients with COPD | *Inclusion criteria:*   - Age ≥ 60 years old - Moderate or severe COPD - At least one hospital admission due to exacerbation in the previous year   *Exclusion criteria:*   - Impaired cognitive function - Illiterate - Hearing problems - Unable to operate the telecare device | Single centre | RHM: 30  no RHM: 23 | Mean  RHM: 65.18 days  no RHM: 68.44 days | - Adherence - ER visits - Health-related quality of life - Hospital admissions - Length of hospitalization - Lung function and other symptoms - Patient experience |
| Dinesen 2012(38)  (Denmark) | NR  (RCT) | To test whether preventive home monitoring in COPD reduced the admission rate to hospital and the cost of hospitalization | *Inclusion criteria:*   - Age > 18 years old - Diagnosis of severe or very severe COPD - Able to understand oral and written information   *Exclusion criteria:*   - Heart disease that could limit physical activity - Mental illness - Terminal malignancy disease - Severe rheumatoid arthritis - Pregnancy | Multiple centres | RHM: 60  no RHM: 51 | 10 months | - Adherence - Cost - Hospital admissions |
| Lewis 2010(39;44)  (UK) | Nov 2007 – Mar 2009  (RCT) | To determine if telemonitoring in stable, and optimized COPD patients affects their health care utilization | *Inclusion criteria:*   - Diagnosis of moderate to severe COPD - Completed at least 12 out of 18 sessions of outpatient PR - Have a GP - Have a standard telephone line   *Exclusion criteria:*   - Chronic asthma and interstitial lung disease - Unstable cardiac disease - Cognitive impairments - Other medical conditions that would unable the use of the intervention - Living in nursing or residential institution - Participation in any investigational drug trial in the last month - Mental condition rendering the patient unable to understand the nature, scope and possible consequences of the study | Single centre | RHM: 20  no RHM: 20 | 12 months | - Adherence - ER visits - Health-related quality of life - Hospital admissions - Length of hospitalization - Mental health - Mortality - Patient experience - Visit to physician |
| Au 2015(40)  (USA) | 2006- 2007  (Observational) | To examine the effects of telemonitoring on resource use among Medicare patients with COPD | *Inclusion criteria:*   - At least a diagnosis of COPD, congestive heart failure, or diabetes mellitus   Exclusion criteria:   - Comorbidities such as dementia or blindness that would limit interaction with the program | Multiple centre | RHM: 619  no RHM: 619 | 3 years | - Adherence - ER visits - Exacerbations - Hospital admissions - Length of hospitalization |
| Davis 2015(41)  (USA) | Oct 2010- Aug 2012  (Retrospective study) | To determine feasibility of a transitional care program that integrated mobile health technology and home visits for underserved COPD and HF patients | *Inclusion criteria:*   - Diagnosis of COPD or HF - Underserved - Able to speak English or Spanish - US residence - Independent in their own care or with reliable caregiver   *Exclusion criteria:*   - End-stage COPD or HF - Hospice candidate - Cancer - Pulmonary fibrosis - On dialysis - Discharged to a setting other than home | Multiple centres | RHM: 58  no RHM: 174 | 3 months | - Adherence - ER visits - Health-related quality of life - Mortality - Patient experience |
| ***RHM with feedback (phone calls, text messages) vs RHM with no feedback*** | | | | | | | |
| Sink 2018(42)  (USA) | Jan 2016- Dec 2016  (RCT) | To study the effect of an automated telemedicine intervention on patients’ time-to-hospitalization. | *Inclusion criteria:*   - Diagnosis of COPD - Age > 18 years old - Willingness to provide a telephone number at which they can receive text messages or voice phone messages   *Exclusion criteria:*   - Intention to transfer care away from the clinic | Single centre | RHM: 83  no RHM: 85 | 8 months | - Adherence - Hospital admissions |
| Franke 2016(43)  (Germany) | Sep 2012- Mar 2015  (RCT) | The primary aim was to compare daily exercise times in patients with stable COPD, either with or without supporting phone calls. | *Inclusion criteria:*   - Moderate to very severe diagnosis of COPD   *Exclusion criteria:*   - Malignancy - Symptomatic cardiac disease. | Single centre | Total: 53* | 6 months | - Adherence - Exercise capacity and activity levels - Health-related quality of life |
| Notes: Tabak 2014a(28) and Tabak 2014b(29) used the same exercise monitoring device and smartphone technology. De San Miguel 2013 (33) and Lewis 2010 (39;44) used the same telemonitoring device. Segrelles 2014 (32) and Jodar-Sanchez 2013 (35) used the same devices to collect vital signs measures and modem technology to transmit collected measurements.  *Cross-over randomized trial.  COPD: chronic obstructive pulmonary disease; FEV_1_: forced expiratory volume in one second; FVC: forced vital capacity; GP: general practitioner; HF: heart failure; PR: pulmonary rehabilitation; RCT: randomized controlled trial; RHM: remote home monitoring | | | | | | | |

| **Table S3. Characteristics of participants included in the studies** | | | | | | | | | | | | |
| --- | --- | --- | --- | --- | --- | --- | --- | --- | --- | --- | --- | --- |
| **Study** | **Sample size** | **Age in years**  **Mean±SD**  **Median (IQR)** | **Gender n(% Male)** | **BMI in kg/m^2^**  **Mean±SD**  **Median (IQR)** | **COPD GOLD stages**  **n (%)** | **FEV_1_ in % predicted**  **Mean±SD**  **Median (IQR)** | **FEV_1_ in l**  **Mean±SD**  **Median (IQR)** | **FVC (l)**  **Mean±SD**  **Median (IQR)** | **FEV_1_/FVC ratio in % Mean±SD**  **Median (IQR)** | **Comorbidities**  **Mean±SD**  **Median (IQR)** | **Smoking status**  **N (%)** | **LTOT**  **N(%)** |
| ***RHM (smartphones, apps, tablets) vs no RHM*** | | | | | | | | | | | | |
| Park 2020  South Korea | RHM: 23  no RHM: 21 | RHM(n=22): 70.4±9.4  no RHM(n=20): 65.1±11.1  p=0.10 | RHM: 20 (87)  no RHM: 15 (71)  p=0.27 | NR | RHM(n=22):  Mild, moderate: 17 (77)  Severe: 5 (23)  Very severe: 0(0)  no RHM(n=20):  Mild, moderate: 16 (80)  Severe: 4 (20)  Very severe: 0(0)  p=1.00 | RHM(n=22):  61.0±18.7  no RHM(n=20):  69.4±24.0  p=0.21 | NR | NR | RHM(n=22):  62.8±20.8  no RHM(n=20):  65.6±17.9  p=0.64 | RHM(n=22)*:  ≥2: 18 (82)  no RHM(n=20)*:  ≥2: 12 (60)  p=0.18 | NR | NR |
| Boer 2019  Netherlands | RHM: 43  no RHM: 44 | RHM: 69.3±8.8  no RHM: 65.9±8.9  p=NR | RHM: 25 (58)  no RHM: 29 (66)  p=NR | NR | NR | RHM: 53.0±21.5  no RHM: 52.1±19.8  p=NR | NR | NR | NR | RHM*:  Joint disorders: 13(30)  Cardiac disorders: 12 (28)  Back pain: 8(19)  Diabetes:3 (7)  Depression and/or anxiety: 3(7)  no RHM*:  Joint disorders: 13(30)  Cardiac disorders: 12(27)  Back pain: 14(32)  Diabetes: 3(7)  Depression and/or anxiety: 2(5)  p=NR | RHM:  Current: 13(30)  no RHM:  Current: 11(25)  p=NR | NR |
| Walker 2018  Spain, UK, Slovenia, Estonia, Sweden | RHM: 154  no RHM:158 | RHM: 71.0 (66.0–75.8)  no RHM: 71.0 (65.3–76.0)  p=NR | RHM: 101 (66)  no RHM: 105 (66)  p=NR | RHM: 27.7 (24.5–30.8)  no RHM: 26.9 (23.8–31.6)  p=NR | RHM:  Mild: 4 (3)  Moderate: 72(47)  Severe: 55 (36)  Very severe: 23 (15)  no RHM:  Mild: 3(2)  Moderate: 76(48)  Severe: 61(39)  Very severe: 18 (11)  p=NR | RHM: 49.4 (37.1–59.2)  no RHM: 50.4 (38.0–63.9)  p=NR | RHM: 1.3 (1.0–1.6)  no RHM: 1.3 (0.9–1.8)  p=NR | RHM: 2.5 (2.0–3.1)  no RHM: 2.5 (2.1–3.1)  p=NR | RHM: 50 (40–60)  no RHM: 50 (40–60)  p=NR | RHM: 2.0 (1.0–3.0)  no RHM: 2.0 (1.0–3.0)  p=NR | NR | NR |
| Tabak 2014a  Netherlands | RHM: 15  no RHM: 14 | RHM(n=12): 64.1±9.0  no RHM(n=12): 62.8±7.4  p=0.71 | RHM(n=12): 6 (50)  no RHM(n=12): 6 (50)  p=1.00 | RHM(n=12): 25.3±4.2  no RHM(n=12): 28.2±4.6  p=0.13 | NR | RHM(n=12): 50.0 (33.3-61.5)  no RHM(n=12): 36.0(26.0, 53.5)  p=0.25 | NR | NR | NR | NR | RHM(n=11):  Smokers:4 (36)  Non-smokers:7 (64)  no RHM(n=12):  Smokers: 4 (33)  Non-smokers: 8 (67)  p=1.00 | NR |
| Tabak 2014b  Netherlands | RHM: 18  no RHM: 16 | RHM(n=14): 65.2±9.0  no RHM: 67.9±5.7  p>0.05 | RHM(n=14): 8 (57)  no RHM: 11 (69)  p>0.05 | RHM(n=14): 28.4±7.8  no RHM: 29.2±4.7  p>0.05 | NR | RHM(n=14): 48.7±16.7  no RHM: 56.4±10.6  p>0.05 | NR | NR | NR | NR | RHM(n=14):  Current smoker: 1 (7)  Former: 13 (93)  no RHM:  Current smoker: 3 (19)  Former: 13 (81)  p>0.05 | NR |
| ***RHM (dedicated monitoring devices) vs no RHM*** | | | | | | | | | | | | |
| Shany 2017  Australia | RHM: 21  no RHM: 21 | RHM: 72.1+7.5  no RHM: 74.2+9.0  p>0.05 | RHM(n=20): 9 (43)  no RHM(n=22): 10 (48)  p>0.05 | RHM: 25.3+7.2  no RHM: 27.4+7.4  p>0.05 | NR | RHM: 32.1+16.0  no RHM: 39.7+13.2  p>0.05 | NR | NR | NR | NR | NR | NR |
| Vianello 2016  Italy | RHM: 230  no RHM: 104 | RHM: 76.0±6.5  no RHM: 76.5±6.2  p=0.95 | RHM: 164(71)  no RHM: 76 (73)  p=0.88 | RHM: 26.5±5.0  no RHM: 26.2±4.9  p=0.59 | RHM:  Mild: 0(0)  Moderate: 0(0)  Severe, very severe: 230 (100)  no RHM:  Mild: 0(0)  Moderate: 0(0)  Severe, very severe: 104 (100)  p=NA | RHM: 41.9±8.6  no RHM: 41.9±8.3  p=0.56 | RHM: 1.1±0.6  no RHM: 1.1±0.5  p=0.99 | NR | NR | RHM*:  Hypertension: 94 (41)  Ischemic heart disease: 60(26)  no RHM:  Hypertension: 51(49)  Ischemic heart disease: 28 (27)  no RHM: NR  p=NR | NR | RHM: 95(41)  no RHM: 41(39)  p=0.76 |
| Segrelles 2014  Spain | RHM: 30  no RHM: 30 | RHM(n=29): 75.0±9.7  no RHM: 72.7±9.3  p=0.36 | RHM(n=29): 22(76)  no RHM: 22 (73)  p=1.00 | NR | RHM:  Mild: 0(0)  Moderate: 0(0)  Severe, very severe: 30 (100)  no RHM:  Mild: 0(0)  Moderate: 0(0)  Severe, very severe: 30 (100)  p=NA | RHM(n=29): 38.3±11.9  no RHM: 37.1±10.8  p=0.52 | NR | NR | NR | RHM(n=29): 3.7±1.4  no RHM: 3.4±2.1  p=0.55 | NR | RHM: 30(100)  no RHM: 30 (100)  p=NA |
| De San Miguel 2013  Australia | RHM: 40  no RHM: 40 | RHM(36): 71 (54-88)**  no RHM(n=35): 74 (57-87)**  p=0.20 | RHM(36): 14 (39)  no RHM(n=35): 20 (57)  p=0.12 | NR | NR | NR | NR | NR | NR | NR | NR | RHM: 40(100)  no RHM: 40 (100)  p=NA |
| Jehn 2013  Germany | RHM: 32  no RHM: 30 | RHM: 64.1±10.9  no RHM: 69.1±9.2  p=0.27 | RHM: 26 (81)  no RHM: 22 (73)  p=0.79 | RHM: 27.6±6.7  no RHM: 27.0±4.9  p=0.87 | RHM:  Mild: 0 (0)  no RHM:  Mild: 0(0)  p=NR | RHM: 50.2±15.0  no RHM: 52.6±17.5  p=0.73 | NR | NR | NR | NR | NR | NR |
| Jodar-Sanchez 2013  Spain | RHM: 24  no RHM: 21 | RHM: 74±8  no RHM: 71±10  p=0.18 | RHM: 23 (96)  no RHM: 20 (95)  p=0.92 | NR | NR | RHM: 38±10  no RHM: 37±13  p=0.33 | NR | RHM: 59±16  no RHM: 63±17  p=0.69 | NR | RHM: 3.7±2.7  no RHM: 2.5±1.5  p=0.18 | NR | RHM: 24(100)  no RHM: 21(100)  p=NA |
| Pare 2013  Canada | RHM: 60  no RHM: 60 | RHM: 67.8+6.4  no RHM: 68.6+6.8  p=0.50 | RHM: 19(32)  no RHM: 19(32)  p=0.99 | NR | NR | RHM:28.6±NR  no RHM: 28.5±NR  p=0.97 | NR | NR | NR | RHM*: ≥1: 15(25)  no RHM*: ≥1: 16(27)  p=0.96 | NR | RHM: 37(62)  no RHM: 36(60)  p=0.85 |
| Chau 2012  Hong Kong | RHM: 30  no RHM: 23 | RHM(n=22): 73.5±6.0  no RHM(n=18): 72.22±6.13  p>0.05 | RHM(n=22): 21(95)  no RHM(n=18): 18(100)  p>0.05 | NR | RHM(n=22):  Mild: 0(0)  Moderate: 4(18)  Severe: 9(41)  Very severe: 9(41)  no RHM(n=18):  Mild: 0(0)  Moderate: 6(33)  Severe: 4(22)  Very severe: 8(44)  p>0.05 | RHM(n=22): 38.8±12.9  no RHM(n=18): 37.7±16.5  p>0.05 | NR | NR | RHM(n=22): 42.1±10.8  no RHM(n=18): 41.0±12.1  p>0.05 | NR | NR | NR |
| Dinesen 2012  Denmark | RHM: 60  no RHM: 51 | RHM(n=57): 68(45-82)  no RHM(n=48): 68(46-89)  p=0.67 | RHM(n=57): 23 (40)  no RHM: NR  p=NR | RHM(n=57): 25.5(16.0-41.0)  no RHM(n=48): 24.6(13.5-38.5)  p=0.14 | RHM:  Mild: 0(0)  Moderate: 0(0)  Severe, very severe: 60(100)  no RHM:  Mild: 0(0)  Moderate: 0(0)  Severe, very severe: 51(100)  p=NA | NR | NR | RHM(n=57): 0.9(0.3-2.1)  no RHM(n=48): 0.9(0.3-2.1)  p=0.36 | NR | NR | NR | NR |
| Lewis 2010  UK | RHM: 20  no RHM: 20 | RHM: 67±9  no RHM: 70±10  p=0.34 | RHM: 10(50)  no RHM: 10(50)  p>0.05 | RHM: 25*.*8±4*.*1  no RHM: 29*.*0±5*.*7  p=0.05 | RHM:  Mild: 0(0)  Moderate, severe: 20(100)  Very severe: 0 (0)  no RHM:  Mild: 0(0)  Moderate, severe: 20(100)  Very severe: 0 (0)  p=NA | RHM: 38±16  no RHM: 40±15  p=0.73 | NR | NR | NR | RHM*:  ≥1: 18(92)  no RHM*:  ≥1: 18(88)  p=0.73 | RHM:  Current: 5 (25)  no RHM:  Current: 5 (25)  p>0.05 | NR |
| Au 2015  USA | RHM: 619  no RHM: 619 | RHM: 75.9±8.7  no RHM: 74.1±10.1  p=0.72 | RHM: 338(55)  no RHM: 338(55)  p=1.00 | NR | NR | NR | NR | NR | NR | RHM: 1.8±1.3  no RHM: 1.3±1.3  p=0.92 | NR | NR |
| Davis 2015  USA | RHM: 58  no RHM: 174 | RHM: 61.0±11.0  no RHM: 63.0±15.8  p>0.05 | RHM: 28 (38)  no RHM: 69 (40)  p>0.05 | NR | NR | NR | NR | NR | NR | NR | NR | NR |
| ***RHM with feedback (phone calls, text messages) vs RHM with no feedback*** | | | | | | | | | | | | |
| Sink 2018  USA | RHM: 83  no RHM: 85 | RHM: 59.9±1.1  no RHM: 61.9±1.1  p=0.18 | RHM: 29 (35)  no RHM: 32 (38)  p=0.72 | NR | RHM(n=63):  Mild: 14 (22)  Moderate: 38 (60)  Severe: 7 (11)  Very severe: 4 (6)  no RHM(n=72):  Mild: 16 (22)  Moderate: 35 (49)  Severe: 16 (22)  Very severe: 5 (7)  p=NR | RHM(n=63):  65±3  no RHM(n=72):  63±2  p=0.69 | NR | NR | RHM(n=63):  64±2  no RHM(n=72):  61±2  p=0.17 | NR | RHM:  Current: 41 (49)  Former: 34 (41)  Never: 3 (4)  Unknown: 5 (6)  no RHM:  Current: 32 (38)  Former: 43 (51)  Never: 4 (5)  Unknown: 6 (7)  p=0.50 | NR |
| Franke 2016  Germany | Total: 53† | Total(n=44)†: 63.3±7.8  p=NA | Total(n=44)†: 20(45)  p=NA | Total(n=44)†: 24.3±5.2  p=NA | Total(n=44)†:  Mild: 0 (0)  Moderate: 18 (14)  Severe: 20 (45)  Very severe: 6 (41)  p=NA | Total(n=44)†: 47.5±15.8  p=NA | NR | NR | NR | NR | NR | NR |
| Notes: Tabak 2014a and Tabak 2014b used the same exercise monitoring device and smartphone technology. De San Miguel 2013 and Lewis 2010 used the same telemonitoring device. Segrelles 2014 and Jodar-Sanchez 2013 used the same devices to collect vital signs measures and modem technology to transmit collected measurements.  *Cross-over randomized trial  *Reported as n(%)  **Reported as mean(range)  † Cross-over randomized trial  BMI: body mass index; COPD: Chronic Obstructive Pulmonary Disease; FEV_1_: Forced Expiratory Volume in one second; FVC: Forced Vital Capacity; IQR: interquartile range; LTOT: long-term oxygen therapy; NR: not reported; RHM: remote home monitoring; SD: standard deviation | | | | | | | | | | | | |

| **Table S4: Remote home monitoring components** | | | | | | | |
| --- | --- | --- | --- | --- | --- | --- | --- |
| **Study** | **User prompts or reminders** | **Mode of data transmission** | **Data administration** | **Frequency of data administration** | **Mechanism for detecting clinically significant changes in health** | **Response to clinically significant changes in health** | **User training provided** |
| ***RHM (smartphones, apps, tablets) vs no RHM*** | | | | | | | |
| Park 2020  South Korea | - Unclear if app prompted daily monitoring or recording - Health care provider contacted patient if data had not been entered into the app on four consecutive days | - Immediately over Internet | - Individual patient data reviewed by health care provider | - Daily for routine data - Immediately for clinically significant changes | - An alert sent to health care provider if clinically significant changes were detected | - Health care provider called patient or texted patient within app | - In person group training provided on how to use the app - Training provided by advanced practice nurse and exercise specialist |
| Boer 2019  Netherlands | - None | - Immediately with Wi-Fi over internet - System capable of operating autonomously with no data transmission | - Optional web-based interface accessible to healthcare providers (not used in this study) | - Immediately - Automated monitoring without the involvement of a health care professional | - Automated decision tree using a Bayesian prediction model - Individual reference values for FEV_1_ and peripheral oxygen saturation set manually at baseline | - Automated self-management advice generated and displayed to patient based on data entered by patient and from physiological measurements | - In person group training provided on how to use the smart mobile health tool - Training provided by nurse - Patients practiced using the smart mobile health tool for two weeks before intervention began |
| Walker 2018  Spain, UK, Slovenia, Estonia, Sweden | - Technical alert prompted staff to contact patient if no data had been entered for two consecutive days | - Immediately over mobile data network | - Automated data administration within system | - Immediately - Automated monitoring without the involvement of a health care professional | - Algorithm generates alert and sends it to nurse if worsening symptoms detected or measurements exceeded predefined thresholds | - Nurse contacts patient when alerted to a worsening of symptoms | - Patients instructed on use of equipment in clinic - Training session repeated after monitoring system installed at home of patient |
| Tabak 2014a  Netherlands | - Patient prompted to record data when logged into web portal - Real-time feedback | - Immediately over internet | - Interactive software provides immediate education and self-management support to patients based on reported symptoms - Individual patient data available to health care provider | - Immediately by device software | - Decision-tree translated data into a decision-support system | - Decision-support system automatically generated advice in response to data captured | - Patients education on self-management embedded in telehealth program - Training on how to access and use the web-based tools not reported |
| Tabak 2014b  Netherlands | - Patient prompted to record data when logged into web portal - Real-time feedback | - Immediately over internet | - Interactive software provides immediate self-management support to patients based on reported symptoms | - Immediately by device software | - Decision- support system | - Decision-support system automatically generated advice in response to data captured | - Patients education on self-management embedded in telehealth program - Training provided by nurse practitioner - Two 90-minute sessions |
| ***RHM (dedicated monitoring devices) vs no RHM*** | | | | | | | |
| Shany 2017  Australia | - None | - Every night or immediately if transfer initiated by patient - Data transmitted via the internet to a secure central server | - Web-based interface accessible to healthcare providers | - Weekdays and occasionally on weekends by nurse | - Alert system on the website flagged patients with measurements outside of predefined thresholds - Nurse reviewed trends on the multi-measurement display to identify patients with potentially deteriorating health | - Nurse contacts patient | - Patients instructed on use of equipment in home - Training provided by nurse - Duration of training dictated by time needed for patient to understand the telehealth unit |
| Vianello 2016  Italy | - No prompts for daily measurements - Prompt from telemonitoring operator for second measurement if alarm triggered - Prompt from health care provider for additional measurements if alarm confirmed by second measurement | - Immediately over telephone line | - Individual patient data reviewed by telemonitoring operator at central data management unit | - Immediately | - Alarm triggered for telehealth operator if measurements outside of normal range - Telemonitoring operators contacted patients to request a second measurement if daily measurements outside of predefined thresholds | - Telemonitoring operator contacts health care provider to alert them if second measurement confirmed to be outside normal range - Health care provider contacts patient by telephone if alerted | - Patients provided self-management materials and instructed on use of equipment in clinic - Clinic training provided by clinical team responsible for patient - Training session repeated after monitoring system installed at home of patient - In-home training provided by technician installing telehealth unit |
| Segrelles 2014  Spain | - Central clinical monitoring centre staff contacted patient if no data had been uploaded | - Immediately via modem over telephone landline | - Individual patient data reviewed by central clinical monitoring centre staff - Individual patient data available to health care provider | - Daily by central clinical monitoring centre staff - Immediately by central clinical monitoring centre staff if alert triggered | - Central server applies algorithm to assign patients to risk categories | - Central clinical monitoring centre staff contacts patient if alert triggered - Individual patient data reviewed immediately by health care provider if clinical alert escalated by central clinical and patient contacted for follow-up | - Patients instructed on use of equipment in home - Patients provided with written instructions on how to use the devices and how to transmit the data - Training provided by nurse - Duration of training not specified |
| De San Miguel 2013  Australia | - None | - Immediately via telephone to a secure website | - Individual patient data reviewed by health care provider | - Daily by health care provider | - Alert triggered if measurements outside of defined parameters | - Health care provider contacts patient by telephone if alerted | - Patients instructed on use of equipment in home - Patients provided with written instructions on how to use the telehealth equipment - Training provided by nurse - Duration of training not specified |
| Jehn 2013  Germany | - Nurse contacted patient if no data had been transferred | - Immediately via mobile network directly to study centre | - Individual patient data reviewed by nurse for completeness and by physician for safety | - Daily by nurse and physician | - Unclear | - Unclear | - Patients instructed on use of equipment in home - Training provided by nurse - Duration of training not specified |
| Jodar-Sanchez 2013  Spain | - Central clinical call centre staff contacted patient if no data had been uploaded | - Immediately when patient presses data transmission button via modem over telephone landline | - Individual patient data reviewed by central clinical call centre staff - Individual patient data available to health care provider | - Daily by central clinical call centre staff - Immediately by central clinical call centre staff if alert triggered | - Central server applies algorithm to assign patients to risk categories | - Central clinical call centre staff contacts patient if alert triggered - Individual patient data reviewed immediately by health care provider if clinical alert escalated by central clinical call centre staff and patient contacted for follow-up | - Patients instructed on use of equipment in home - Training provided by nurse - Duration of training not specified |
| Pare 2013  Canada | - Device prompted patient to enter data if not entered on date prescribed - Real-time feedback | - Immediately over internet | - Interactive system provides immediate self-management support to patients based on reported symptoms - Individual patient data monitored by nurse | - Immediately by device software | - Alert triggered if measurements outside of defined parameters | - Warning issued to patient immediately - Alert sent to nurse to review data and follow-up directly with patient if needed | - Patients education on self-management embedded in telehealth program - Patients instructed on use of equipment in home - Training provided by nurse - Duration of training not specified |
| Chau 2012  Hong Kong | - Unclear | - Immediately through General Packet Radio Service to a base station | - Individual patient data monitored by nurse | - Daily by health care provider | - Nurse monitors data for changes in measurements outside of parameters | - Nurse monitors data and takes immediate action if changes in patient physiological parameters detected | - Patients instructed on self-management and use of equipment in home - Training provided by nurse - Duration of training not specified |
| Dinesen 2012  Denmark | - Not reported | - Immediately using wireless technology via a secure line | - Individual patient data monitored by health care providers | - Not reported | - Individual patient data monitored by health care providers | - Health care providers offer advice to the patient | - Patients instructed on exercises and use of equipment in home - Training provided by health care providers - Duration of training not specified |
| Lewis 2010  UK | - Health care provider contacted patient by phone or sent a message to the monitor’s screen if there were seven consecutive days without a data upload | - Daily two times per day at specified time via home telephone line | - Individual patient data accessible to health care provider | - Daily two times per day at specified times | - Alert triggered and email automatically sent to Chronic Disease Management Team and copied to the hospital respiratory nurses if measurements outside of defined parameters | - Chronic Disease Management Team member contacts patient by telephone if alert triggered | - Patients instructed on use of equipment in home - Training provided by technical implementation team - One training session less than one hour |
| Au 2015  USA | - Device prompted patient to enter data - Device provided feedback to patients based on reported symptoms | - Immediately over internet | - Interactive device software provides immediate education and self-management support to patients based on reported symptoms - Individual patient data reviewed by health care provider | - Immediately by device software - Upon completion of daily session by central server - Daily by health care provider | - Central server applies algorithm to assign patients to risk categories | - Health care provider monitors patient data daily and follow-up with the patient directly based on patient’s calculate risk | - Training not described |
| Davis 2015  USA | - Device prompted patient to enter data - Device provided feedback to patients based on reported symptoms - Telehealth company’s monitoring staff contacted patient if no data had been uploaded for three consecutive days | - Immediately over telephone landline or wireless | - Interactive device software provides immediate education and self-management support to patients based on reported symptoms - Individual patient data monitored by telehealth company’s monitoring staff - Individual patient data available to health care provider | - Immediately by device software - Immediately by telehealth company’s monitoring staff if alert sent triggered by device software - Unclear how frequently health care providers reviewed data | - Alert triggered by telehealth unit if acute changes in symptomatology detected | - Telehealth company’s monitoring staff contacts patient if alert triggered by device software - Health care provider notified by telehealth company’s monitoring staff if alert requires follow-up | - Patients instructed on self-management and use of equipment in home - Training provided by nurse - Duration of training not specified |
| ***RHM with feedback (phone calls, text messages) vs RHM with no feedback*** | | | | | | | |
| Sink 2018  USA | - Automated system contacted patient by telephone or text | - Immediately over land telephone lines or cellular network | - Automated data administration within system | - Immediately - Automated monitoring without the involvement of a health care professional | - Automated decision tree using a Bayesian prediction model | - Provider engagement platform triggers alert to health care provider if patient reports worse symptoms | - None required |
| Franke 2016  Germany | - None | - Immediately over internet | - Individual patient data reviewed by health care provider | - Weekly | - Average weekly training time calculated - An average weekly training time of less than 20 minutes per day was threshold for patient follow-up | - Health care provider contacts patient for a five minute motivational telephone call | - Patients instructed on use of equipment in home |
| Notes: Tabak 2014a and Tabak 2014b used the same exercise monitoring device and smartphone technology. De San Miguel 2013 and Lewis 2010 used the same telemonitoring device. Segrelles 2014 and Jodar-Sanchez 2013 used the same devices to collect vital signs measures and modem technology to transmit collected measurements. | | | | | | | |

| Table S5: Description of remote home monitoring programs and technology | | | | | | |
| --- | --- | --- | --- | --- | --- | --- |
| Study | Duration  of Remote Home Monitoring | Comparator | Remote Home Monitoring Intervention | Remote Home Monitoring Technology | Remote Home Monitoring Technology Description | Data Capture |
| *RHM (smartphones, apps, tablets) vs no RHM* | | | | | | |
| *Park 2020*  *South Korea* | 6 months | - Four weekly group education sessions - Four weekly group exercise sessions - Personalized exercise prescription - Exercise pamphlet - Monthly telephone call to check on health status | - Four weekly group education sessions - Four weekly group exercise sessions - Personalized exercise prescription - Exercise pamphlet - Educational material available on smartphone app - Videos of exercises available on smart phone app - Daily self-monitoring and recording in smartphone app - Communication with other patients within app - Minimum monthly communication with providers through text messaging within smartphone app or telephone call | - Smartphone app - Additional devices: Pedometer | - Downloadable COPD self-management smartphone app for Android platform - Adjustable font size - Pedometer (independent device not paired with app) | - Manual data entry by patient |
| *Boer 2019*  *Netherlands* | 12 months | - Group education session on exacerbation self-management - Exacerbation self-management support using a paper-based action plan (considered Usual Care) - Follow-up with nurse at 3 months to evaluate exacerbation self-management | - Group education session on exacerbation self-management - Exacerbation self-management support using a smart mobile health tool - Two-week daily trial use of smart mobile health tool - Nurse evaluation of patient use of system after two-week trial period - Reference values for FEV_1_ and peripheral oxygen saturation set in smart mobile health tool - Patients instructed to use smart mobile health tool whenever they experienced a change in symptoms or disease burden - Smart mobile health tool provided automated self-management advice tailored to the individual patient - Follow-up with nurse at 3 months to evaluate exacerbation self-management | - Mobile phone - Smartphone app - Sensor-interface - Peripheral devices: Pulse Oximeter, Spirometer, Forehead thermometer | - Smartphone - Adaptive Computerized COPD Exacerbation Self-management Support System (ACCESS) - Disease-specific probabilistic model to tailor automated advice - Bluetooth communication with sensors | - Bluetooth communication with sensors |
| *Walker 2018*  *Spain, UK, Slovenia, Estonia, Sweden* | 9 months | - Usual Care - Nurse contacted patients every three months to assess medication use | - Daily measurement of physiological symptoms and health status by patient - Nurse contacts patient when alerted to a worsening of symptoms - Technical alert prompted staff to contact patient if no data entered for two consecutive days - Nurse contacted patients every three months to assess medication use | - Telehealth measurement unit with integrated measurement device - Peripheral devices: wearable device to measure blood pressure, oxygen saturation, heart rate and temperature | - Hand-held touchscreen computer with integrated device measuring within-breath respiratory mechanical impedance - Wearable device - Mobile modem | - Integrated device - Additional manual data entry by patient |
| *Tabak 2014a*  *Netherlands* | 9 months | - Usual care | - Technology supported interactive telehealth program - Program modules include teleconsultation, a web-based exercise program, automated activity coach, and a self-management education module | - Interactive web-based telehealth program - Mobile phone - Smartphone app - Peripheral device: accelerometer-based activity sensor | - Smartphone - Web portal - Interactive smartphone app - Wearable Bluetooth enabled activity sensor | - Manual data entry by patient - Peripheral device captured measurements in smartphone app |
| *Tabak 2014b*  *Netherlands* | 1 month | - Usual care | - Technology supported interactive telehealth program - Program included an automated activity coach, and an automated self-management decision-support system - Patients attended two 90-minute self-management education sessions delivered by a nurse practitioner - The first week of the activity coach monitoring involved data capture for baseline reference - Three weeks of interactive activity coaching and activity monitoring at least four days per week | - Interactive web-based telehealth program - Mobile phone - (Smart mobile health tool) Smartphone app - Peripheral device: accelerometer-based activity sensor | - Smartphone - Web portal - Wearable Bluetooth enabled activity sensor | - Manual data entry by patient - Peripheral device captured measurements in smartphone app |
| *RHM (dedicated monitoring devices) vs no RHM* | | | | | | |
| *Shany 2017*  *Australia* | 12 months | - Usual care - Weekly scheduled home visits - Urgent home visits by respiratory community nurse (no maximum) - Twenty-four hour, seven days per week nurse managed phone line - Scheduled visits to a respiratory rehabilitation outpatient clinic | - Weekly scheduled home visits - Urgent home visits by respiratory community nurse (no maximum) - Twenty-four hour, seven days per week nurse managed phone line - Scheduled visits to a respiratory rehabilitation outpatient clinic - Daily monitoring of health with home telehealth measurement unit - Ad hoc measurement with immediate transmission of health measurements | - Telehealth measurement unit - Peripheral devices: differential pressure spirometer, tubular grips for electrocardiogram, pulse oximeter sensor, blood pressure measurement, weight scale, glucometer and thermometer | - Telehealth measurement unit with touchscreen user interface - Wireless communication between peripheral devices and telehealth unit - Website for health care providers to monitor patient data | - Integrated and peripheral devices captured measurements in telehealth unit |
| *Vianello 2016*  *Italy* | 12 months | - Usual care | - Daily monitoring of health with home telemonitoring measurement unit - Ad hoc measurement with immediate transmission of health measurements in the event of worsening condition - Telemonitoring operators contacted patients to request a second measurement if daily measurements outside of normal range - Telemonitoring operator contacts health care provider to alert them if second measurement confirmed to be outside normal range - Health care provider contacts patient by telephone if alerted | - Wearable telehealth measurement unit with integrated finger pulse oximeter - Peripheral device: Gateway device - Telephone | - Wireless wearable device - Gateway device for data transmission over a telephone line to a central data management unit - Website for health care providers to access patient data | - Integrated device captured measurements automatically |
| *Segrelles 2014*  *Spain* | 7 months | - Usual care | - Daily monitoring of health with home telemonitoring measurement unit - Clinical monitoring centre contacted patients when alerted by system - Clinical monitoring centre escalated to alert the pneumologist who contacted the patient by telephone to determine appropriate action | - Telehealth measurement units with integrated measurement devices - Integrated devices: spirometer, peak flow meter, pulse oximeter, heart rate monitor and blood pressure monitor | - Telehealth measurement units with integrated measurement devices - Data transmission via modem over telephone line to central clinical monitoring centre - Central clinical monitoring centre with customized monitoring software | - Integrated devices captured measurements in telehealth unit |
| *De San Miguel 2013*  *Australia* | 6 months | - Usual care - Telehealth nurse visited patients at home once and provided patients with educational booklet on COPD - No other contact with the telehealth nurse - Patients were provided with a calendar to record health care service use | - Telehealth nurse visited patients at home once and provided patients with educational booklet on COPD - Telehealth nurse provided training on the telehealth equipment and provided patients with a telehealth instruction manual - Patients provided with a home telemonitoring device - Patients recorded physiological symptoms, and completed standardized assessments daily - Nurse contacts patient when alerted to a worsening of symptoms - Patients were provided with a calendar to record health care service use | - Telehealth monitoring device - Integrated and peripheral devices: weight scale, blood pressure monitor, pulse oximeter and thermometer | - Small portable telehealth measurement unit with an integrated display, large keys - Data transmission via modem over telephone line to secure website | - Manual data entry by patient - Some integrated devices captured measurements in telehealth unit - Unclear if peripheral devices required manual data entry |
| *Jehn 2013*  *Germany* | 9 months | - Usual care - Initial baseline visit - Follow-up visits at three, six and nine months | - Usual care - Initial Baseline visit - Follow-up visits at three, six and nine months - Patients provided with a home telemonitoring device - Patients completed measurements in the morning during a two-hour time window and recorded physiological symptoms, and completed standardized assessments - Nurse available to provide technical support to patients on weekdays during regular business hours - Nurse contacted patients to remind them to complete the monitoring if not completed in measurement window - Incoming data reviewed by physician for safety | - Telehealth measurement units with integrated or peripheral devices - Integrated or peripheral devices: spirometer, and accelerometer | - Handheld mobile medical assistant personal data assistant system - Hand held spirometer - Accelerometer | - Manual data entry by patient - Integrated device captured measurements - Unclear if peripheral device required manual data entry |
| *Jodar-Sanchez 2013*  *Spain* | 4 months | - Usual care - Initial visit by nurse to patient home for baseline | - Initial visit by nurse to patient home for baseline - Daily monitoring of health with home telemonitoring measurement unit - Clinical call centre contacted patients when alerted by system - Clinical call centre escalated to alert the case manager and a specialist in respiratory medicine who contacted the patient by telephone to determine appropriate action | - Telehealth measurement units with integrated measurement devices - Integrated devices: spirometer, pulse oximeter, heart rate monitor and blood pressure monitor | - Telehealth measurement units with integrated measurement devices - Data transmission via modem over telephone line to central clinical monitoring centre - Central clinical monitoring centre with customized monitoring software | - Integrated devices captured measurements in telehealth unit |
| *Pare 2013*  *Canada* | 12 months | - Usual care | - Patients provided with a home telemonitoring and self-management educational device - Patients completed a data entry table documenting symptoms and medication daily - Patients read education modules on self-management on device daily - Health care provider monitors patient data daily and follow-up with the patient directly | - Telehealth monitoring and self-management device | - Telehealth device was a tablet with a touch screen and integrated modem - Customized follow-up protocol programmed into device | - Manual data entry by patient |
| *Chau 2012*  *Hong Kong* | 2 months | - Usual care - Home visit by nurse to conduct education on self-management techniques | - Usual care - Home visit by nurse to conduct education on self-management techniques - Remote monitoring device kit provided to patient for use three times a day - Nurse provided training on the telehealth equipment | - Telehealth monitoring device with peripheral devices - Peripheral devices: respiratory rate sensor and pulse oximeter | - Hand held telemonitor (Specially designed mobile phone telehealth measurement unit) - Wearable Bluetooth enabled peripheral devices - Online network platform to record and monitor health data - Nurse-led call centre | - Integrated devices captured measurements in telehealth unit |
| *Dinesen 2012*  *Denmark* | 4 months | - Instruction provided on how to perform home exercises - Patients responsible for performing exercises at home - No planned contact with health care providers regarding home exercise | - Instruction provided on how to perform home exercises - Telehealth monitoring device installed in patient home - Training on how to use equipment - Physician prescribed frequency of measurement - Data monitored by health care providers to enable feedback - Monthly video meeting with tele-rehabilitation team to discuss and coordinate individual patient programs | - Telehealth monitoring device with peripheral devices - Peripheral devices: blood pressure monitor, weight scale, spirometer and pulse oximeter - Additional device: pedometer | - Telehealth measurement units with peripheral measurement devices - Using wireless technology, the telehealth monitor can collect and transmit data via a secure line - Data transmitted to web-based portal or patient’s electronic health record | - Integrated devices captured measurements in telehealth unit |
| *Lewis 2010*  *UK* | 26 weeks | - Usual care - Instructed to seek help from their GP or an emergency doctor if urgent treatment was needed | - Usual care - Instructed to seek help from their GP or an emergency doctor if urgent treatment was needed - Telemonitoring for twenty-six weeks followed by no telemonitoring for twenty-six weeks | - Telehealth monitoring device with peripheral devices - Peripheral device: pulse oximeter - Additional device: thermometer | - Handheld telemonitor with integrated or peripheral devices - Central server accessible to health care providers | - Manual data entry by patient - Peripheral device connected to telemonitor captured measurements |
| *Au 2015*  *USA* | 12 months | - Intervention and control patients were propensity-score matched 1:1 using administrative data | - Patients provided with a home telemonitoring and self-management device - Patients prompted daily by device to measure and record physiological symptom, and complete standardized assessments - Interactive device provides immediate education and self-management support to patients based on reported symptoms - Health care provider monitors patient data daily and follow-up with the patient directly based on patient’s calculated risk | - Telehealth monitoring and self-management device - Peripheral or additional devices not clearly reported | - Telehealth measurement unit with high resolution screen and four buttons to collect information from patients and provide interactive advice - Unclear if or how data is transmitted from peripheral devices to telehealth unit - Website for health care providers to monitor patient data | - Manual data entry by patient - Unclear how peripheral or additional devices capture measurements in telehealth unit |
| *Davis 2015*  *USA* | 3 months | - Non-treated propensity-score matched retrospective cohort | - Patients provided with educational materials on self-management - Nurse reviewed educational material with patients before hospital discharge or at initial home visit - Home visit one week post hospital discharge - Nurse completed medication reconciliation at home visit - Personal disease management plan developed - Patients provided with an interactive remote monitoring device - Additional home visits as needed - Final home visit ninety days from initial visit | - Telehealth monitoring and self-management device - Integrated and peripheral devices: weight scale, blood pressure, pulse oximeter | - Telehealth measurement unit with interactive voice response system and response buttons | - Manual data entry by patient - Integrated and peripheral devices captured measurements in telehealth unit |
| *RHM with feedback (phone calls, text messages) vs RHM with no feedback* | | | | | | |
| *Sink 2018*  *USA* | 8 months | - Daily automated phone call or text message to assess breathing - Automated message frequency reduced to twice weekly if 30 consecutive days of no worsening in breathing reported - Automated message frequency temporarily resumed to daily if breathing assessment detected a worsening in breathing - No alerts sent to health care provider based on response to automated breathing assessment - Self-directed access to health care provider | - Daily automated phone call or text message to assess breathing - Automated message frequency reduced to twice weekly if 30 consecutive days of no worsening in breathing reported - Automated message frequency temporarily resumed to daily if breathing assessment detected a worsening in breathing - Alerts sent to health care provider if patient response to automated breathing assessment indicated a worsening in breathing - Health care provider contacts patient when alerted to a worsening of symptoms | - Automated phone call or text message with alerts | - Patient engagement platform contacts patient by automated phone call or text message - Provider engagement platform triggers alert to health care provider if patient reports worse symptoms - Bidirectional communication enabled between patients and health care providers | - Patient replies to automated question by typing response into telephone or text message |
| *Franke 2016*  *Germany* | 3 months | - Home exercise cycle training | - Home exercise cycle training - Weekly motivational telephone calls | - Telephone - Bicycle ergometer with data transmission system - Peripheral devices: Magnetic switch to measure bicycle pedal movement | - Bicycle ergometer equipped with a data transmission system and integrated micro-controller - Magnetic switch recorded pedal movement | - Integrated and peripheral devices captured measurements automatically |

| **Table S6: Remote home monitoring components** | | | | | | | |
| --- | --- | --- | --- | --- | --- | --- | --- |
| **Study** | **User prompts or reminders** | **Mode of data transmission** | **Data administration** | **Frequency of data administration** | **Mechanism for detecting clinically significant changes in health** | **Response to clinically significant changes in health** | **User training provided** |
| ***RHM (smartphones, apps, tablets) vs no RHM*** | | | | | | | |
| *Park 2020*  *South Korea* | - Unclear if app prompted daily monitoring or recording - Health care provider contacted patient if data had not been entered into the app on four consecutive days | - Immediately over Internet | - Individual patient data reviewed by health care provider | - Daily for routine data - Immediately for clinically significant changes | - An alert sent to health care provider if clinically significant changes were detected | - Health care provider called patient or texted patient within app | - In person group training provided on how to use the app - Training provided by advanced practice nurse and exercise specialist |
| *Boer 2019*  *Netherlands* | - None | - Immediately with Wi-Fi over internet - System capable of operating autonomously with no data transmission | - Optional web-based interface accessible to healthcare providers (not used in this study) | - Immediately - Automated monitoring without the involvement of a health care professional | - Automated decision tree using a Bayesian prediction model - Individual reference values for FEV_1_ and peripheral oxygen saturation set manually at baseline | - Automated self-management advice generated and displayed to patient based on data entered by patient and from physiological measurements | - In person group training provided on how to use the smart mobile health tool - Training provided by nurse - Patients practiced using the smart mobile health tool for two weeks before intervention began |
| *Walker 2018*  *Spain, UK, Slovenia, Estonia, Sweden* | - Technical alert prompted staff to contact patient if no data had been entered for two consecutive days | - Immediately over mobile data network | - Automated data administration within system | - Immediately - Automated monitoring without the involvement of a health care professional | - Algorithm generates alert and sends it to nurse if worsening symptoms detected or measurements exceeded predefined thresholds | - Nurse contacts patient when alerted to a worsening of symptoms | - Patients instructed on use of equipment in clinic - Training session repeated after monitoring system installed at home of patient |
| *Tabak 2014a*  *Netherlands* | - Patient prompted to record data when logged into web portal - Real-time feedback | - Immediately over internet | - Interactive software provides immediate education and self-management support to patients based on reported symptoms - Individual patient data available to health care provider | - Immediately by device software | - Decision-tree translated data into a decision-support system | - Decision-support system automatically generated advice in response to data captured | - Patients education on self-management embedded in telehealth program - Training on how to access and use the web-based tools not reported |
| *Tabak 2014b*  *Netherlands* | - Patient prompted to record data when logged into web portal - Real-time feedback | - Immediately over internet | - Interactive software provides immediate self-management support to patients based on reported symptoms | - Immediately by device software | - Decision- support system | - Decision-support system automatically generated advice in response to data captured | - Patients education on self-management embedded in telehealth program - Training provided by nurse practitioner - Two 90-minute sessions |
| ***RHM (dedicated monitoring devices) vs no RHM*** | | | | | | | |
| *Shany 2017*  *Australia* | - None | - Every night or immediately if transfer initiated by patient - Data transmitted via the internet to a secure central server | - Web-based interface accessible to healthcare providers | - Weekdays and occasionally on weekends by nurse | - Alert system on the website flagged patients with measurements outside of predefined thresholds - Nurse reviewed trends on the multi-measurement display to identify patients with potentially deteriorating health | - Nurse contacts patient | - Patients instructed on use of equipment in home - Training provided by nurse - Duration of training dictated by time needed for patient to understand the telehealth unit |
| *Vianello 2016*  *Italy* | - No prompts for daily measurements - Prompt from telemonitoring operator for second measurement if alarm triggered - Prompt from health care provider for additional measurements if alarm confirmed by second measurement | - Immediately over telephone line | - Individual patient data reviewed by telemonitoring operator at central data management unit | - Immediately | - Alarm triggered for telehealth operator if measurements outside of normal range - Telemonitoring operators contacted patients to request a second measurement if daily measurements outside of predefined thresholds | - Telemonitoring operator contacts health care provider to alert them if second measurement confirmed to be outside normal range - Health care provider contacts patient by telephone if alerted | - Patients provided self-management materials and instructed on use of equipment in clinic - Clinic training provided by clinical team responsible for patient - Training session repeated after monitoring system installed at home of patient - In-home training provided by technician installing telehealth unit |
| *Segrelles 2014*  *Spain* | - Central clinical monitoring centre staff contacted patient if no data had been uploaded | - Immediately via modem over telephone landline | - Individual patient data reviewed by central clinical monitoring centre staff - Individual patient data available to health care provider | - Daily by central clinical monitoring centre staff - Immediately by central clinical monitoring centre staff if alert triggered | - Central server applies algorithm to assign patients to risk categories | - Central clinical monitoring centre staff contacts patient if alert triggered - Individual patient data reviewed immediately by health care provider if clinical alert escalated by central clinical and patient contacted for follow-up | - Patients instructed on use of equipment in home - Patients provided with written instructions on how to use the devices and how to transmit the data - Training provided by nurse - Duration of training not specified |
| *De San Miguel 2013*  *Australia* | - None | - Immediately via telephone to a secure website | - Individual patient data reviewed by health care provider | - Daily by health care provider | - Alert triggered if measurements outside of defined parameters | - Health care provider contacts patient by telephone if alerted | - Patients instructed on use of equipment in home - Patients provided with written instructions on how to use the telehealth equipment - Training provided by nurse - Duration of training not specified |
| *Jehn 2013*  *Germany* | - Nurse contacted patient if no data had been transferred | - Immediately via mobile network directly to study centre | - Individual patient data reviewed by nurse for completeness and by physician for safety | - Daily by nurse and physician | - Unclear | - Unclear | - Patients instructed on use of equipment in home - Training provided by nurse - Duration of training not specified |
| *Jodar-Sanchez 2013*  *Spain* | - Central clinical call centre staff contacted patient if no data had been uploaded | - Immediately when patient presses data transmission button via modem over telephone landline | - Individual patient data reviewed by central clinical call centre staff - Individual patient data available to health care provider | - Daily by central clinical call centre staff - Immediately by central clinical call centre staff if alert triggered | - Central server applies algorithm to assign patients to risk categories | - Central clinical call centre staff contacts patient if alert triggered - Individual patient data reviewed immediately by health care provider if clinical alert escalated by central clinical call centre staff and patient contacted for follow-up | - Patients instructed on use of equipment in home - Training provided by nurse - Duration of training not specified |
| *Pare 2013*  *Canada* | - Device prompted patient to enter data if not entered on date prescribed - Real-time feedback | - Immediately over internet | - Interactive system provides immediate self-management support to patients based on reported symptoms - Individual patient data monitored by nurse | - Immediately by device software | - Alert triggered if measurements outside of defined parameters | - Warning issued to patient immediately - Alert sent to nurse to review data and follow-up directly with patient if needed | - Patients education on self-management embedded in telehealth program - Patients instructed on use of equipment in home - Training provided by nurse - Duration of training not specified |
| *Chau 2012*  *Hong Kong* | - Unclear | - Immediately through General Packet Radio Service to a base station | - Individual patient data monitored by nurse | - Daily by health care provider | - Nurse monitors data for changes in measurements outside of parameters | - Nurse monitors data and takes immediate action if changes in patient physiological parameters detected | - Patients instructed on self-management and use of equipment in home - Training provided by nurse - Duration of training not specified |
| *Dinesen 2012*  *Denmark* | - Not reported | - Immediately using wireless technology via a secure line | - Individual patient data monitored by health care providers | - Not reported | - Individual patient data monitored by health care providers | - Health care providers offer advice to the patient | - Patients instructed on exercises and use of equipment in home - Training provided by health care providers - Duration of training not specified |
| *Lewis 2010*  *UK* | - Health care provider contacted patient by phone or sent a message to the monitor’s screen if there were seven consecutive days without a data upload | - Daily two times per day at specified time via home telephone line | - Individual patient data accessible to health care provider | - Daily two times per day at specified times | - Alert triggered and email automatically sent to Chronic Disease Management Team and copied to the hospital respiratory nurses if measurements outside of defined parameters | - Chronic Disease Management Team member contacts patient by telephone if alert triggered | - Patients instructed on use of equipment in home - Training provided by technical implementation team - One training session less than one hour |
| *Au 2015*  *USA* | - Device prompted patient to enter data - Device provided feedback to patients based on reported symptoms | - Immediately over internet | - Interactive device software provides immediate education and self-management support to patients based on reported symptoms - Individual patient data reviewed by health care provider | - Immediately by device software - Upon completion of daily session by central server - Daily by health care provider | - Central server applies algorithm to assign patients to risk categories | - Health care provider monitors patient data daily and follow-up with the patient directly based on patient’s calculate risk | - Training not described |
| *Davis 2015*  *USA* | - Device prompted patient to enter data - Device provided feedback to patients based on reported symptoms - Telehealth company’s monitoring staff contacted patient if no data had been uploaded for three consecutive days | - Immediately over telephone landline or wireless | - Interactive device software provides immediate education and self-management support to patients based on reported symptoms - Individual patient data monitored by telehealth company’s monitoring staff - Individual patient data available to health care provider | - Immediately by device software - Immediately by telehealth company’s monitoring staff if alert sent triggered by device software - Unclear how frequently health care providers reviewed data | - Alert triggered by telehealth unit if acute changes in symptomatology detected | - Telehealth company’s monitoring staff contacts patient if alert triggered by device software - Health care provider notified by telehealth company’s monitoring staff if alert requires follow-up | - Patients instructed on self-management and use of equipment in home - Training provided by nurse - Duration of training not specified |
| ***RHM with feedback (phone calls, text messages) vs RHM with no feedback*** | | | | | | | |
| *Sink 2018*  *USA* | - Automated system contacted patient by telephone or text | - Immediately over land telephone lines or cellular network | - Automated data administration within system | - Immediately - Automated monitoring without the involvement of a health care professional | - Automated decision tree using a Bayesian prediction model | - Provider engagement platform triggers alert to health care provider if patient reports worse symptoms | - None required |
| *Franke 2016*  *Germany* | - None | - Immediately over internet | - Individual patient data reviewed by health care provider | - Weekly | - Average weekly training time calculated - An average weekly training time of less than 20 minutes per day was threshold for patient follow-up | - Health care provider contacts patient for a five minute motivational telephone call | - Patients instructed on use of equipment in home |
| Notes: Tabak 2014a and Tabak 2014b used the same exercise monitoring device and smartphone technology. De San Miguel 2013 and Lewis 2010 used the same telemonitoring device. Segrelles 2014 and Jodar-Sanchez 2013 used the same devices to collect vital signs measures and modem technology to transmit collected measurements. | | | | | | | |

| Table S7: Risk of bias | | | | | | | | | | | | | | | | | | | | | |
| --- | --- | --- | --- | --- | --- | --- | --- | --- | --- | --- | --- | --- | --- | --- | --- | --- | --- | --- | --- | --- | --- |
| **Risk of bias of RCTs** | | | | | | | | | | | | | | | | | | | | | |
| **Study** | **Selection bias** | | | | | **Performance bias** | | | **Detection bias** | | | | | **Attrition bias** | | | **Reporting bias** | | | **Other bias** | |
|  | **Random sequence generation** | **Description** | **Allocation concealment** | **Description** | **Blinding of participants and personnel** | | **Description** | **Blinding of outcome assessment (patient reported outcomes)** | | **Description** | **Blinding of outcome assessment (other outcomes)** | **Description** | **Incomplete outcome data** | | **Description** | **Selective reporting** | | **Description** | **Other bias** | | **Description** |
| ***RHM (smartphones, apps, tablets) vs no RHM*** | | | | | | | | | | | | | | | | | | | | | |
| Park 2020  South Korea | Low | Computer-generated randomization | Unclear | No information provided | High | | Owing to the type of intervention, patients and health care professionals could not be blinded to group assignment | High | | The outcome assessor (patients) were not blinded to the intervention | High | “The interventionist and outcome assessor were the same person, which could threaten internal validity.” | Low | | Missing data were small and similar across groups  Characteristics of patients who dropped out were similar to those remaining in the study  The intention to treat principle was applied to all statistical analyses | High | | No trial registration was found  Study did not present results of all domains of some tools | Low | | Study appears to be free from other sources of bias |
| Boer 2019  Netherlands | Low | Computer-generated randomization | Unclear | No information provided | High | | Owing to the type of intervention, patients and health care professionals could not be blinded to group assignment | High | | The outcome assessor (patients) were not blinded to the intervention | Low | Research team was not blinded to assignment, but outcomes were unlikely to be influenced by knowledge of intervention received | Low | | The number of patients who did not complete the study was similar in the intervention and control groups | Low | | Trial was registered  All of the study's pre-specified outcomes were reported | Low | | Study appears to be free from other sources of bias |
| Sink 2018  USA | High | 17 patients were added to control group without randomization. | Unclear | Insufficient information provided | High | | Owing to the type of intervention, patients and health care professionals could not be blinded to group assignment | N/A | | N/A | Low | Whether the assessor was blinded was unclear, but outcomes were unlikely to be influenced by knowledge of intervention received | High | | Patients in the intervention group were censored from analysis the day they stopped messages  For the control group, patients were censored from the analysis on the last day data were collected | Low | | All of the study’s pre-specified outcomes have been reported | Low | | Study appears to be free from other sources of bias |
| Walker 2018    Spain, UK, Slovenia, Estonia, Sweden | Low | Computer-generated randomization | Unclear | No information provided | High | | Owing to the type of intervention, patients and health care professionals could not be blinded to group assignment | High | | The outcome assessors (patients) were not blinded to the intervention | Low | Data were analyzed by an independent party and outcomes were unlikely to be influenced by knowledge of intervention received | Low | | Intention-to-treat analysis was used and multiple imputation with 20 iteration was conducted | Low | | Study protocol was available in the online supplement  All of the study’s pre-specified outcomes were reported | Unclear | | Study criteria for hospitalization and COPD care were not standardized across the multiple centres involved in the study |
| ***RHM (dedicated monitoring devices) vs no RHM*** | | | | | | | | | | | | | | | | | | | | | |
| Shany 2017  Australia | Unclear | No information provided | Unclear | Insufficient information provided | High | | Owing to the type of intervention, patients and health care professionals could not be blinded to group assignment | High | | The outcome assessors (patients) were not blinded to the intervention | Low | Assessors were blinded and outcomes were unlikely to be influenced by knowledge of intervention received | High | | The number of missing data was but still significantly different between intervention and control | Unclear | | No protocol or study registration was provided | Low | | Study appears to be free from other sources of bias |
| Franke 2016  Germany | Unclear | Insufficient information provided | Unclear | No information provided | High | | Owing to the type of intervention, patients and health care professionals could not be blinded to group assignment | High | | The outcome assessors (patients) were not blinded to the intervention | Low | It is unclear if the assessor was blinded, but outcomes were unlikely to be influenced by knowledge of intervention received | Unclear | | Nine patients had missing data  No information on differences between patients who completed the study and those who dropped out was presented | Unclear | | Trial registration was provided, but it did not contain enough information | High | | Case-crossover design resulted in potential bias: patients who started with the intervention had higher daily training time in the control phase compared to those first randomized to the control phase |
| Vianello 2016  Italy | Low | Computer-generated randomization | Unclear | No information provided | High | | Owing to the type of intervention, patients and health care professionals could not be blinded to group assignment | High | | The outcome assessors (patients) were not blinded to the intervention | Low | It is unclear if the assessor was blinded, but outcomes were unlikely to be influenced by knowledge of intervention received | Low | | Missing data was small (21% in the intervention group and 22% in the control group) | Low | | All of the study’s pre-specified outcomes were reported | Low | | Study appears to be free from other sources of bias |
| Tabak 2014a  Netherlands | High | While patients were randomized using a computer-generated randomization list, the groups were not comparable -Dyspnea scores in the intervention group were significantly better than those in the control group at baseline | Low | Participants were allocated by a data manager in order of inclusion following the randomization list, placed in a sealed envelope. | High | | Owing to the type of intervention, patients and health care professionals could not be blinded to group assignment | High | | The outcome assessors (patients) were not blinded to the intervention | Unclear | Insufficient information provided | High | | The percentages of drop-outs in the intervention and control groups were 33% and 86%, respectively | High | | No protocol or study registration was provided  Authors collected data on exacerbations and other outcomes at 6 and 9 months of follow-up, but none of these data were reported | Low | | Study appears to be free from other sources of bias |
| Tabak 2014b    Netherlands | Low | Computer-generated randomization | Unclear | Insufficient information provided | High | | Owing to the type of intervention, patients and health care professionals could not be blinded to group assignment | High | | The outcome assessors (patients) were not blinded to the intervention | Low | Assessors were not blinded but outcomes were unlikely to be influenced by knowledge of intervention received | Low | | The amount of missing data was small | Unclear | | No protocol or trial registration was provided | Low | | Study appears to be free from other sources of bias |
| Segrelles 2014  Spain | Low | Drawing of lots | High | Allocation was by centre  Patients in selected intervention centres were all included in the intervention  Investigators could foresee assignment prior to randomization | High | | Owing to the type of intervention, patients and health care professionals could not be blinded to group assignment | N/A | | N/A | Unclear | Insufficient information was provided | Low | | The amount of missing data was small | Low | | All of the study’s pre-specified outcomes were reported | low | | Study appears to be free from other sources of bias |
| De San Miguel 2013  Australia | Low | Computer-generated randomization | Unclear | Insufficient information provided | High | | Owing to the type of intervention, patients and health care professionals could not be blinded to group assignment | High | | The outcome assessor (participants) were not blinded to the intervention | N/A | N/A | Low | | Amount of missing data was small and similar across groups | High | | No protocol or trial registration was found  Study mentioned differences in health-related quality of life outcomes across groups but no actual data were. were presented | low | | Study appears to be free from other sources of bias |
| Jehn 2013  Germany | Unclear | No information provided | Unclear | No information provided | High | | Owing to the type of intervention, patients and health care professionals could not be blinded for group assignment | High | | The outcome assessor (participants) were not blinded to the intervention | Unclear | No information provided | Low | | Amount of missing data was small and similar across groups | Unclear | | No protocol or trial registration was found | Low | | Study appears to be free from other sources of bias |
| Jodar-Sanchez 2013  Spain | high | Baseline SGRQ scores are statistically significantly different between groups | Unclear | No information provided | High | | Owing to the type of intervention, patients and health care professionals could not be blinded o group assignment | High | | The outcome assessor (patients) were not blinded to the intervention | Unclear | No information provided | Low | | As only one patient per group was lost due to death; therefore, the probability of missing data was low    Analysis was based on all patients | Unclear | | No protocol was mentioned  It is unclear is if the outcomes in the study were pre-defined | Low | | Study appears to be free from other sources of bias |
| Pare 2013  Canada | Unclear | No information provided | Low | “The members of each pair were randomly and blindly assigned to either the control group or the experimental group” | High | | Owing to the type of intervention, patients and health care professionals could not be blinded to group assignment | High | | The outcome assessor (patients) were not blinded to the intervention | Unclear | No information provided | Low | | The characteristics of patients who completed the study were similar to those with missing data | Unclear | | There was no mention of study protocol or a trial registration  It is unclear if the outcomes were pre-determined | High | | Patients were recruited between September 2010 and March 2011 and follow-up period ended in October 2011 for all patients  The length of follow-up was different among participants  The study did not provide enough information to clarify how or if any adjustments were made |
| Chau 2012  Hong Kong | Low | Drawing of lots | Unclear | Insufficient information provided | High | | Owing to the type of intervention, patients and health care professionals could not be blinded to group assignment | High | | The outcome assessors (patients) were not blinded to the intervention | High | “No blinding of outcome assessors may have introduced bias” | High | | 73% and 78% of patients completed the study in the intervention and control groups, respectively  The amount of missing data and reasons for dropping out differed between groups | Unclear | | No protocol or trial registration was available | Low | | Study appears to be free from other sources of bias |
| Dinesen 2012  Denmark | Low | Drawing of lots | Low | The envelopes were sealed, blinding health-care professionals, patients and researchers to allocation assignment | High | | Owing to the type of intervention, patients and health care professionals could not be blinded to group assignment | N/A | | N/A | Low | It is unclear if the assessor was blinded, but outcomes were unlikely to be influenced by knowledge of intervention received | Low | | Number of drop outs was small  Missing outcomes data were balanced in across groups | High | | Study was published in 2012  According to authors, "results on health-related quality of life will be reported in a future paper"  However, no published papers containing such information could be found | Low | | Study appears to be free from other sources of bias |
| Lewis 2010  UK | High | Patients were randomised into two groups using a random number generator  Patients in the intervention group had a significantly longer period since completing PR at baseline | Low | “Allocation used sealed opaque envelopes” | High | | Owing to the type of intervention, patients and health care professionals could not be blinded to group assignment | High | | The outcome assessor (patients) were not blinded to the intervention | Unclear | No information provided | Unclear | | No information provided | High | | Study did not report findings on all domains of SGRQ | Low | | Study appears to be free from other sources of bias |

| Table S8. Health-related quality of life - CAT, CCQ and other instruments | | | | | | | | | | | |
| --- | --- | --- | --- | --- | --- | --- | --- | --- | --- | --- | --- |
| **Study** | **Sample size** | **Time** | **CAT** | | | **CCQ** | | | **Other HRQoL measures** | | |
|  |  |  | **RHM**  **Mean±SD** | **Comparison group**  **Mean±SD** | **Diff. between groups**  **MD(95%CI)** | **RHM**  **Mean±SD** | **Comparison group**  **Mean±SD** | **Diff. between groups**  **MD(95%CI)** | **RHM**  **Mean±SD** | **Comparison group**  **Mean±SD** | **Diff. between groups**  **MD(95%CI)** |
| ***RHM (smartphones, apps, tablets) vs no RHM*** | | | | | | | | | | | |
| Park 2020(25)  South Korea | RHM: 23  no RHM: 21 | Baseline | NR | NR | NR | NR | NR | NR | **SF-36 (PCS):**  RHM(n=23): 43.4±9.0  **SF-36 (MCS):**  RHM(n=23): 51.6±8.7 | **SF-36 (PCS):**  no RHM(n=20): 46.4±5.6  **SF-36 (MCS):**  no RHM(n=20): 52.1±8.5 | **SF-36 (PCS):**  p=0.22  **SF-36 (MCS):**  p=0.85 |
|  |  | 6 months  *Diff. within group* | NR | NR | NR | NR | NR | NR | **SF-36 (PCS):**  RHM(n=23): 43.9±9.0  *p>0.05*  **SF-36 (MCS):**  RHM(n=23): 50.1±8.3  *p>0.05* | **SF-36 (PCS):**  no RHM(n=20): 44.9±5.9  *p>0.05*  **SF-36 (MCS):**  no RHM(n=20): 49.0±11.0  *p>0.05* | **SF-36 (PCS):**  NR  p=0.36  **SF-36 (MCS):**  NR  p=0.60 |
| Boer 2019(26)  Netherlands | RHM: 43  no RHM: 44 | Baseline | NR | NR | NR | **Total:**  RHM: 2.1±1.0  **Symptoms:**  RHM: 2.4±1.1  **Functional status:**  RHM: 2.2±1.4  **Mental status:**  RHM: 1.0±1.0 | **Total:**  no RHM: 2.3±1.1  **Symptoms:**  no RHM: 2.6±1.3  **Functional status:**  no RHM: 2.5±1.4  **Mental status:**  no RHM: 1.3±1.0 | NR | **NCSI (QOL):**  RHM: 12.9±8.3  **NCSI (HRQOL):**  RHM: 4.2±1.7  **NCSI (Relationship):**  RHM: 2.4±0.9  **EQ-5D:**  RHM: 0.81±0.15  **EQ-5D (VAS):**  RHM: 65.53±17.37 | **NCSI (QOL):**  no RHM: 19.1±11.9  **NCSI (HRQOL):**  no RHM: 4.7±1.8  **NCSI (Relationship):**  no RHM: 3.4±1.7  **EQ-5D:**  no RHM: 0.74±0.20  **EQ-5D (VAS):**  no RHM: 64.20±15.35 | NR |
|  |  | 12 months | NR | NR | NR | **Total:**  RHM(n=35): 1.8±0.8  **Symptoms:**  RHM(n=35): 2.2±0.8  **Functional status:**  RHM(n=35): 2.0±1.2  **Mental status:**  RHM(n=35): 0.8±0.8 | **Total:**  no RHM(n=41): 2.2±1.0  **Symptoms:**  no RHM(n=41): 2.5±1.2  **Functional status:**  no RHM(n=41): 2.4±1.3  **Mental status:**  no RHM(n=41): 1.0±1.0 | **Total:**  −0.1 (−0.4, 0.3)  p=0.70  **Symptoms:**  −0.2 (−0.7, 0.2)  p=0.34  **Functional status:**  0.0 (−0.3, 0.4)  p=0.76  **Mental status:**  0.1 (−0.3, 0.5)  p=0.68 | **NCSI (QOL):**  RHM(n=35): 13.0±8.3  **NCSI (HRQOL):**  RHM(n=35): 4.0±1.6  **NCSI (Relationship):**  RHM(n=35): 2.6±0.9  **EQ-5D:**  RHM(n=35): 0.79±0.16  **EQ-5D (VAS):**  RHM(n=35): 70.94±12.92 | **NCSI (QOL):**  no RHM(n=41): 17.1±12.1  **NCSI (HRQOL):**  no RHM(n=41): 4.8±1.93  **NCSI (Relationship):**  no RHM(n=41): 3.2±1.6  **EQ-5D:**  no RHM(n=41): 0.77±0.21  **EQ-5D (VAS):**  no RHM(n=41): 62.63±19.14 | **NCSI (QOL):**  2.53 (−1.3, 6.3)  p=0.19  **NCSI (HRQOL):**  −0.2 (−0.9, 0.6)  p=0.66  **NCSI (Relationship):**  0.3 (−0.3, 0.8)  p=0.29  **EQ-5D:**  −0.05 (−0.13, 0.03)  p=0.22  **EQ-5D (VAS):**  6.28 (−0.56, 13.11)  p=0.07 |
| Walker 2018 (27)  Spain, UK, Slovenia, Estonia, Sweden | RHM: 154  no RHM: 158 | Baseline | RHM: 17.4±7.9 | no RHM: 17.7±8.4 | NR | NR | NR | NR | **EQ-5D:**  RHM: 0.641±0.224  **EQ-5D (VAS):**  RHM: 58.80±17.76 | **EQ-5D:**  no RHM: 0.663±0.225  **EQ-5D (VAS):**  no RHM: 57.32±20.72 | NR |
|  |  | 8 months | RHM(n=150): 16.8±7.7 | no RHM(n=154): 17.2±8.3 | NR  p=0.66 | NR | NR | NR | **EQ-5D:**  RHM(n=150): 0.637±0.225  **EQ-5D (VAS):**  RHM(n=150): 55.35±18.46 | **EQ-5D:**  no RHM(n=153): 0.640± 0.248  **EQ-5D (VAS):**  no RHM(N=153): 55.75±21.17 | **EQ-5D:**  NR  p=0.91  **EQ-5D (VAS):**  NR  p=0.87 |
| Tabak 2014a(28)  Netherlands | RHM: 15  no RHM: 14 | Baseline | NR | NR | NR | **Total:**  RHM(n=12): 2.0±0.3 | **Total:**  no RHM(n=12): 2.7±0.3 | p=0.08 | **EQ-5D:**  RHM(n=12):0.76±0.03  **EQ-5D (VAS):**  RHM(n=12):64.7±4.4 | **EQ-5D:**  no RHM(n=12):0.70±0.04  **EQ-5D (VAS):**  no RHM(n=12):65.0±4.5 | NR |
|  |  | 1 month | NR | NR | NR | **Total:**  RHM(n=11): 1.9±0.2 | **Total:**  no RHM(n=9): 2.3±0.2 | NR | **EQ-5D:**  RHM(n=11):0.81±0.03  **EQ-5D (VAS):**  RHM(n=11):73.1±4.0 | **EQ-5D:**  no RHM(n=9):0.72±0.03  **EQ-5D (VAS):**  no RHM(n=9):69.3±4.9 | NR |
|  |  | 3 months | NR | NR | NR | **Total:**  RHM(n=11): 1.8±0.2 | **Total:**  no RHM(n=9): 2.3±0.3 | NR | **EQ-5D:**  RHM(n=11): 0.78±0.08  **EQ-5D (VAS):**  RHM(n=11): 72.3±3.1 | **EQ-5D:**  no RHM(n=9): 0.61±0.09  **EQ-5D (VAS):**  no RHM(n=9): 62.4±3.5 | NR |
| Tabak 2014b  Netherlands (29) | RHM: 18  no RHM: 16 | Baseline | NR | NR | NR | **Total:**  RHM(n=14): 2.0±0.8 | **Total:**  no RHM(n=16): 1.8±1.0 | NR | NR | NR | NR |
|  |  | 1 month  *Diff. within group*  *MD±SD* | NR | NR | NR | **Total:**  RHM(n=14): NR  *-0.3±0.5*  *p=0.04* | **Total:**  no RHM(n=16): NR  *0.0±0.6*  *p=0.89* | NR (-0.1, 0.8)  p=0.10 | NR | NR | NR |
| ***RHM (dedicated monitoring devices) vs no RHM*** | | | | | | | | | | | |
| Shany 2017(30)  Australia | RHM: 21  no RHM: 21 | Baseline | NR | NR | NR | NR | NR | NR | NR | NR | NR |
| Vianello 2016 (31)  Italy | RHM: 230  no RHM: 104 | Baseline | RHM: 15.2±8.2 | no RHM: 14.0±6.8 | p=0.37 | NR | NR | NR | **SF-36 (PCS):**  RHM: 39.2±9.7  **SF-36(MCS):**  RHM: 45.6±11.2 | **SF-36 (PCS):**  no RHM: 38.39 ±8.98  **SF-36(MCS):**  no RHM: 44.98 ±10.72 | **SF-36 (PCS):**  p=0.59  **SF-36(MCS):**  p=0.31 |
|  |  | 12 months  *Diff. within group*  *MD±SD* | NR | NR | NR |  |  |  | **SF-36 (PCS):**  RHM(n=181): 37.1±9.1  *-2.1±9.0*  *p<0.01*  **SF-36(MCS):**  RHM(n=181): 44.6±10.9  *-1.1±11.3*  *p=NR* | **SF-36 (PCS):**  no RHM(n=81): 36.5± 8.6  *-1.9±7.7*  *p=0.02*  **SF-36(MCS):**  no RHM(n=81): 43.1±10.9  *-1.9±10.9*  *p=NR* | **SF-36 (PCS):**  -0.2 (-2.4, 2.1)  p=0.89  **SF-36(MCS):**  -0.8 (-2.1, 3.8)  p=0.57 |
| Segrelles 2014(32)  Spain | RHM: 30  no RHM: 30 | NR | NR | NR | NR | NR | NR | NR | NR | NR | NR |
| De San Miguel 2013(33)  Australia | RHM: 40  no RHM: 40 | NR | NR | NR | NR | NR | NR | NR | NR | NR | NR |
| Jehn 2013(34)  Germany | RHM: 32  no RHM: 30 | Baseline | RHM(=27): 19.0±6.9 | no RHM(n=25): 17.6±5.0 | NR | NR | NR | NR | NR | NR | NR |
|  |  | 9 months  *Diff. within group*  *MD±SD* | RHM(=27): 16.0±5.6  *-2.9±4.5*  *p=0.04* | no RHM(n=25): 22.0±6.9  *4.4±5.7*  *p=0.01* | NR | NR | NR | NR | NR | NR | NR |
| Jodar-Sanchez 2013(35)  Spain | RHM: 24  no RHM: 21 | Baseline | NR | NR | NR | NR | NR | NR | **EQ-5D:**  RHM: 0.44±0.27 | **EQ-5D:**  no RHM: 0.55±0.33 | **EQ-5D:**  p=0.24 |
|  |  | 4 months  *Diff. within group*  *MD±SD* | NR | NR | NR | NR | NR | NR | **EQ-5D:**  RHM: NR  *0.04±0.28*  *p=NR* | **EQ-5D:**  no RHM: NR  *0.00±0.24*  *p=NR* | **EQ-5D:**  0.03±0.08*  p=0.68 |
| Pare 2013(36)  Canada | RHM: 60  no RHM: 60 | NR | NR | NR | NR | NR | NR | NR | NR | NR | NR |
| Chau 2012(37)  Hong Kong | RHM: 30  no RHM: 23 | NR | NR | NR | NR | NR | NR | NR | NR | NR | NR |
| Dinesen 2012(38)  Denmark | RHM: 60  no RHM: 51 | NR | NR | NR | NR | NR | NR | NR | NR | NR | NR |
| Lewis 2010(39;44)  UK | RHM: 20  no RHM: 20 | Baseline | NR | NR | NR | NR | NR | NR | **EQ-5D:**  RHM: 0*.*54±0*.*27 | **EQ-5D:**  no RHM: 0*.*51±0*.*27 | 0.60 |
|  |  | 12 months | NR | NR | NR | NR | NR | NR | NR | NR | NR  p=0.45 |
| Au 2015(40)  USA | RHM: 619  no RHM: 619 | NR | NR | NR | NR | NR | NR | NR | NR | NR | NR |
| Davis 2015(41)  USA | RHM: 69  no RHM: 174 | Baseline | NR | NR | NR | **Total:**  RHM: 3.8±0.9  **Functional status:**  RHM:3.4±1.4  **Mental health:**  RHM:3.9±1.7 | NR | NR | NR | NR | NR |
|  |  | NR  *Diff. within group MD* | NR | NR | NR | **Total:**  RHM: 1.9±1.0  *1.9*  **Functional status:**  RHM: 2.0±1.0  *1.4*  **Mental health:**  RHM: 1.8±1.8  *2.0* | NR | NR | NR | NR | NR |
| ***RHM with feedback vs RHM with no feedback*** | | | | | | | | | | | |
| Sink 2018(42)  USA | RHM: 83  no RHM: 85 | NR | NR | NR | NR | NR | NR | NR | NR | NR | NR |
| Franke 2016(43)  Germany | Total: 53 | Baseline | Total(n=44)*: 17.6±6.1 | | NA | NR | NR | NR | NR | NR | NR |
|  |  | 3 months | Total(n=44)*: 15.4±8.0 | | NA | NR | NR | NR | NR | NR | NR |
|  |  | 6 months | RHM phase(n=44)*: 15.3±7.6  p=0.01 | no RHM phase(n=44)*: 15.7±7.3  p=0.02 | NR | NR | NR | NR | NR | NR | NR |
| Notes: De San Miguel 2013 (33) and Lewis 2010 (39;44) used the same telemonitoring device. Segrelles 2014 (32) and Jodar-Sanchez 2013 (35) used the same devices to collect vital signs measures and modem technology to transmit collected measurements.  *Cross-over randomized trial.  CAT: COPD assessment test; CCQ: Clinical COPD Questionnaire; CI: confidence interval; COPD: chronic obstructive pulmonary disease; EQ-5D: Euroqol 5D; HRQOL: health-related quality of life; MCS: mental component summary; MD: mean difference; MFI: Multidimensional Fatigue Inventory; NA: not applicable; NCSI: Nijmegen Clinical Screening Instrument; NR: not reported; PCS: physical component summary; QOL: quality of life; RHM: remote home monitoring; SD: standard deviation; SF-36: 36-item Short Form Survey | | | | | | | | | | | |

| Table S9. Health related quality of life - CRQ | | | | | | | | | | | | | | |
| --- | --- | --- | --- | --- | --- | --- | --- | --- | --- | --- | --- | --- | --- | --- |
| **Study** | **Sample size** | **Time** | **Dyspnoea** | | | **Emotional function** | | | **Fatigue** | | | **Mastery** | | |
|  |  |  | **RHM**  **Mean±SD**  **Median(IQR)** | **Comparison group**  **Mean±SD**  **Median(IQR)** | **Diff. between groups**  **MD(95%CI)** | **RHM**  **Mean±SD**  **Median(IQR)** | **Comparison group**  **Mean±SD**  **Median(IQR)** | **Diff. between groups**  **MD(95%CI)** | **RHM**  **Mean±SD**  **Median(IQR)** | **Comparison group**  **Mean±SD**  **Median(IQR)** | **Diff. between groups**  **MD(95%CI)** | **RHM**  **Mean±SD**  **Median(IQR)** | **Comparison group**  **Mean±SD**  **Median(IQR)** | **Diff. groups**  **MD(95%CI)** |
| ***RHM (smartphones, apps, tablets) vs no RHM*** | | | | | | | | | | | | | | |
| Park 2020(25)  South Korea | RHM: 23  no RHM: 21 | NR | NR | NR | NR | NR | NR | NR | NR | NR | NR | NR | NR | NR |
| Boer 2019(26)  Netherlands | RHM: 43  no RHM: 44 | NR | NR | NR | NR | NR | NR | NR | NR | NR | NR | NR | NR | NR |
| Walker 2018 (27)  Spain, UK, Slovenia, Estonia, Sweden | RHM: 154  no RHM: 158 | NR | NR | NR | NR | NR | NR | NR | NR | NR | NR | NR | NR | NR |
| Tabak 2014a(28)  Netherlands | RHM: 15  no RHM: 14 | NR | NR | NR | NR | NR | NR | NR | NR | NR | NR | NR | NR | NR |
| Tabak 2014b (29)  Netherlands | RHM: 18  no RHM: 16 | NR | NR | NR | NR | NR | NR | NR | NR | NR | NR | NR | NR | NR |
| ***RHM (dedicated monitoring devices) vs no RHM*** | | | | | | | | | | | | | | |
| Shany 2017(30)  Australia | RHM: 21  no RHM: 21 | NR | NR | NR | NR | NR | NR | NR | NR | NR | NR | NR | NR | NR |
| Vianello 2016 (31)  Italy | RHM: 230  no RHM: 104 | NR | NR | NR | NR | NR | NR | NR | NR | NR | NR | NR | NR | NR |
| Segrelles 2014(32)  Spain | RHM: 30  no RHM: 30 | NR | NR | NR | NR | NR | NR | NR | NR | NR | NR | NR | NR | NR |
| De San Miguel 2013(33)  Australia | RHM: 40  no RHM: 40 | Baseline | RHM: NR | no RHM: NR | p>0.05 | RHM: NR | no RHM: NR | p>0.05 | RHM: NR | no RHM: NR | p>0.05 | RHM: NR | no RHM: NR | p>0.05 |
|  |  | 6 months | RHM: NR | no RHM: NR | NR  p>0.05 | RHM: NR | no RHM: NR | NR  p>0.05 | RHM: NR | no RHM: NR | NR  p>0.05 | RHM: NR | no RHM: NR | NR  p>0.05 |
| Jehn 2013(34)  Germany | RHM: 32  no RHM: 30 |  | NR | NR | NR | NR | NR | NR | NR | NR | NR | NR | NR | NR |
| Jodar-Sanchez 2013(35)  Spain | RHM: 24  no RHM: 21 | NR | NR | NR | NR | NR | NR | NR | NR | NR | NR | NR | NR | NR |
| Pare 2013(36)  Canada | RHM: 60  no RHM: 60 | NR | NR | NR | NR | NR | NR | NR | NR | NR | NR | NR | NR | NR |
| Chau 2012(37)  Hong Kong | RHM: 30  no RHM: 23 | Baseline | RHM(n=22): 4.27±1.23 | no RHM(n=18): 4.20±0.83 | p>0.05 | RHM(n=22): 4.84±1.47 | no RHM(n=18): 5.24±1.42 | p>0.05 | RHM(n=22): 4.09±1.26 | no RHM(n=18): 4.40±0.99 | p>0.05 | RHM(n=22): 4.60±1.43 | no RHM(n=18): 4.94±1.16 | p>0.05 |
|  |  | mean  Home monitoring: 65.18 days  Usual care: 68.44 days | RHM(n=22): 3.97±1.17 | no RHM(n=18): 4.45±0.96 | NR  p=0.11 | RHM(n=22): 4.92±1.40 | no RHM(n=18): 5.61±1.17 | NR  p=0.37 | RHM(n=22): 4.11±1.25 | no RHM(n=18): 4.79±1.07 | NR  p=0.27 | RHM(n=22): 4.61±1.62 | no RHM(n=18): 4.88±1.27 | NR  p=0.84 |
| Dinesen 2012(38)  Denmark | RHM: 60  no RHM: 51 | NR | NR | NR | NR | NR | NR | NR | NR | NR | NR | NR | NR | NR |
| Lewis 2010(39;44)  UK | RHM: 20  no RHM: 20 | NR | NR | NR | NR | NR | NR | NR | NR | NR | NR | NR | NR | NR |
| Au 2015(40)  USA | RHM: 619  no RHM: 619 | NR | NR | NR | NR | NR | NR | NR | NR | NR | NR | NR | NR | NR |
| Davis 2015(41)  USA | RHM: 69  no RHM: 174 | NR | NR | NR | NR | NR | NR | NR | NR | NR | NR | NR | NR | NR |
| ***RHM with feedback vs RHM with no feedback*** | | | | | | | | | | | | | | |
| Sink 2018(42)  USA | RHM: 83  no RHM: 85 | NR | NR | NR | NR | NR | NR | NR | NR | NR | NR | NR | NR | NR |
| Franke 2016(43)  Germany | Total: 53 | NR | NR | NR | NR | NR | NR | NR | NR | NR | NR | NR | NR | NR |
| Notes: Tabak 2014a(28) and Tabak 2014b(29) used the same exercise monitoring device and smartphone technology. De San Miguel 2013 (33) and Lewis 2010 (39;44) used the same telemonitoring device. Segrelles 2014 (32) and Jodar-Sanchez 2013 (35) used the same devices to collect vital signs measures and modem technology to transmit collected measurements.  CI: confidence interval; CRQ: Chronic Respiratory Disease Questionnaire; IQR: interquartile range; MD: mean difference; NR: not reported; RHM: remote home monitoring; SD: standard deviation | | | | | | | | | | | | | | |

| Table S10. Health related quality of life - SGRQ | | | | | | | | | | | | | | |
| --- | --- | --- | --- | --- | --- | --- | --- | --- | --- | --- | --- | --- | --- | --- |
| **Study** | **Sample size** | **Time** | **Symptoms** | | | **Activity** | | | **Impact** | | | **Total score** | | |
|  |  |  | **RHM**  **Mean±SD** | **Comparison group**  **Mean±SD** | **Diff. between groups**  **MD(95%CI)** | **RHM**  **Mean±SD** | **Comparison group**  **Mean±SD** | **Diff. between groups**  **MD(95%CI)** | **RHM**  **Mean±SD** | **Comparison group**  **Mean±SD** | **Diff. between groups**  **MD(95%CI)** | **RHM**  **Mean±SD** | **Comparison group**  **Mean±SD** | **Diff. between groups**  **MD(95%CI)** |
| ***RHM (smartphones, apps, tablets) vs no RHM*** | | | | | | | | | | | | | | |
| Park 2020(25)  South Korea | RHM: 23  no RHM: 21 | NR | NR | NR | NR | NR | NR | NR | NR | NR | NR | NR | NR | NR |
| Boer 2019(26)  Netherlands | RHM: 43  no RHM: 44 | NR | NR | NR | NR | NR | NR | NR | NR | NR | NR | NR | NR | NR |
| Walker 2018 (27)  Spain, UK, Slovenia, Estonia, Sweden | RHM: 154  no RHM: 158 | Baseline | NR | NR | NR | NR | NR | NR | NR | NR | NR | RHM: 46.2 (35.6–64.3) | no RHM: 50.9 (34.7–63.4) | NR |
| Tabak 2014a(28)  Netherlands | RHM: 15  no RHM: 14 | NR | NR | NR | NR | NR | NR | NR | NR | NR | NR | NR | NR | NR |
| Tabak 2014b (29)  Netherlands | RHM: 18  no RHM: 16 | NR | NR | NR | NR | NR | NR | NR | NR | NR | NR | NR | NR | NR |
| ***RHM (dedicated monitoring devices) vs no RHM*** | | | | | | | | | | | | | | |
| Shany 2017(30)  Australia | RHM: 21  no RHM: 21 | Baseline | NR | NR | NR | NR | NR | NR | NR | NR | NR | RHM: 62.8±14.0 | no RHM: 58.2±17.8 | p>0.05 |
|  |  | 12 months | NR | NR | NR | NR | NR | NR | NR | NR | NR | RHM: NR | no RHM: NR | NR  p>0.05 |
| Vianello 2016 (31)  Italy | RHM: 230  no RHM: 104 | NR | NR | NR | NR | NR | NR | NR | NR | NR | NR | NR | NR | NR |
| Segrelles 2014(32)  Spain | RHM: 30  no RHM: 30 | NR | NR | NR | NR | NR | NR | NR | NR | NR | NR | NR | NR | NR |
| De San Miguel 2013(33)  Australia | RHM: 40  no RHM: 40 | NR | NR | NR | NR | NR | NR | NR | NR | NR | NR | NR | NR | NR |
| Jehn 2013(34)  Germany | RHM: 32  no RHM: 30 | Baseline | NR | NR | NR | NR | NR | NR | NR | NR | NR | RHM: 51.3±18.7 | no RHM: 48.7±14.7 | p=0.56 |
| Jodar-Sanchez 2013(35)  Spain | RHM: 24  no RHM: 21 | Baseline | RHM: 63±23 | no RHM: 44±23 | p=0.01 | RHM: 87±20 | no RHM: 75±24 | p=0.02 | RHM: 61±20 | no RHM: 51±25 | p=0.18 | RHM: 69±17 | no RHM: 57±22 | p=0.05 |
|  |  | 4 months  *Diff. within group MD±SD* | NR  *-12.8±32.6*  *p=NR* | no RHM: NR  *-3.7±19.6*  *p=NR* | 9.1±8.1*  p=0.31 | RHM: NR  *-13.5±25.4*  *p=NR* | no RHM: NR  *-4.0±37.9*  *p=NR* | 9.5±8.2*  p=0.26 | RHM: NR  *-8.7±24.1*  *p=NR* | no RHM: NR  *-2.1±23.2*  *p=NR* | 6.7±7.2*  p=0.38 | RHM: NR  *-10.9±21.9*  *p=NR* | no RHM: NR  *-4.5±19.7*  *p=NR* | 6.4±6.3*  p=0.53 |
| Pare 2013(36)  Canada | RHM: 60  no RHM: 60 | NR | NR | NR | NR | NR | NR | NR | NR | NR | NR | NR | NR | NR |
| Chau 2012(37)  Hong Kong | RHM: 30  no RHM: 23 | NR | NR | NR | NR | NR | NR | NR | NR | NR | NR | NR | NR | NR |
| Dinesen 2012(38)  Denmark | RHM: 60  no RHM: 51 | NR | NR | NR | NR | NR | NR | NR | NR | NR | NR | NR | NR | NR |
| Lewis 2010(39;44)  UK | RHM: 20  no RHM: 20 | Baseline | NR | NR | NR | NR | NR | NR | NR | NR | NR | RHM: 60*.*7±15*.*3 | no RHM: 59*.*9±15*.*2 | NR  p=0.83 |
|  |  | 12 months | NR | NR | NR | NR | NR | NR | NR | NR | NR | NR | NR | p=0.90 |
| Au 2015(40)  USA | RHM: 619  no RHM: 619 | NR | NR | NR | NR | NR | NR | NR | NR | NR | NR | NR | NR | NR |
| Davis 2015(41)  USA | RHM: 69  no RHM: 174 | NR | NR | NR | NR | NR | NR | NR | NR | NR | NR | NR | NR | NR |
| ***RHM with feedback vs RHM with no feedback*** | | | | | | | | | | | | | | |
| Sink 2018(42)  USA | RHM: 83  no RHM: 85 | NR | NR | NR | NR | NR | NR | NR | NR | NR | NR | NR | NR | NR |
| Franke 2016(43)  Germany | RHM: 51  no RHM: 51 | NR | NR | NR | NR | NR | NR | NR | NR | NR | NR | NR | NR | NR |
| Note: Tabak 2014a(28) and Tabak 2014b(29) used the same exercise monitoring device and smartphone technology. De San Miguel 2013 (33) and Lewis 2010 (39;44) used the same telemonitoring device. Segrelles 2014 (32) and Jodar-Sanchez 2013 (35) used the same devices to collect vital signs measures and modem technology to transmit collected measurements.  *Reported as mean±SE  CI: confidence interval; MD: mean difference; NR: not reported; RHM: remote home monitoring; SD: standard deviation; SGRQ: St. George’s respiratory questionnaire; SE: standard error | | | | | | | | | | | | | | |

| Table S11. Patient experience and satisfaction with RHM | | | | | |
| --- | --- | --- | --- | --- | --- |
| **Study** | **Sample size** | **Method** | **Findings** | | |
|  |  |  | **Experience using technology** | **Perceived benefits and experience with disease management** | **Overall satisfaction** |
| ***RHM (smartphones, apps, tablets) vs no RHM*** | | | | | |
| Park 2020(25)  South Korea | RHM: 23  no RHM: 21 | - Patients were interviewed about their experience in the program. - One open ended question about patient’s experience in the program. - Four questions on an 11-point rating scale ranging from 0 to 100%. Higher scores indicated higher support/satisfaction. | NR | **Support disease management:**  RHM(n=22): 95.9± 9.6  no RHM(n=20): 91.0± 13.3  p=0.18  **Support for symptom management:**  RHM(n=22): 95.0± 9.6  no RHM(n=20): 91.0± 10.2  p=0.20  **Support for increasing physical activity and reducing sedentary time:**  RHM(n=22): 93.2± 12.9  no RHM(n=20): 85.5±13.9  p=0.07 | **Overall satisfaction- mean±SD:**  RHM(n=22): 94.5± 9.6  no RHM(n=20): 89.5± 10.5  p= 0.11 |
| Boer 2019(26)  Netherlands | RHM: 43  no RHM: 44 | - Patients were asked to complete the system usability scale (SUS) which generated one total score ranging from 0 to 100. - SUS<68 are considered low - 68<SUS<80.3 are considered good - SUS>80.3 are considered excellent | **SUS overall score– Mean±SD:**  RHM(n=26): 78.5±14.4 | NR | NR |
| Walker 2018 (27)  Spain, UK, Slovenia, Estonia, Sweden | RHM: 154  no RHM: 158 | NR | NR | NR | NR |
| Tabak 2014a(28)  Netherlands | RHM: 15  no RHM: 14 | - Patients were asked to complete the Client Satisfaction Questionnaire 8. - The total score range was 8–32, and a higher score indicated a higher degree of client satisfaction. | NR | NR | **Overall satisfaction- mean±SE:**  *1 month:*  RHMN(n=11): 26.4±1.3  no RHM(n=9): 30.4±1.5  p=NR  *3 months:*  RHM(n=11): 26.3±1.3  no RHM(n=9): 29.9±1.4  p=NR |
| Tabak 2014b (29)  Netherlands | RHM: 18  no RHM: 16 | NR | NR | NR | NR |
| ***RHM (dedicated monitoring devices) vs no RHM*** | | | | | |
| Shany 2017(30)  Australia | RHM: 21  no RHM: 21 | - Patients were asked about usability of the system. No further details provided. | - Within the first 2 weeks, three patients reported that RHM technology was difficult to use. However, at four months, no comments like these were received. - At the end of study, all remaining patients disagreed that technology was hard to use. One patient reported difficulties using the technology and withdrew from the study. - Apart from 1, the remaining patients enjoyed using the intervention. | - Patients reported that technology allowed them better management of the disease. | **Recommend to others -n (%recommend):**  RHM(n=11): 11(100)  **Wish to continue with RHM- n(%yes):**  RHM(n=11): 9(82) |
| Vianello 2016 (31)  Italy | RHM: 230  no RHM: 104 | NR | NR | NR | NR |
| Segrelles 2014(32)  Spain | RHM: 30  no RHM: 30 | NR | NR | NR | NR |
| De San Miguel 2013(33)  Australia | RHM: 40  no RHM: 40 | - In-person interviews with patients. No further details provided. | - Patients reported that equipment was easy to use - Entering of daily data took 5 minutes - Taking own measurements was reassuring | - Half of the patients reported perceived reduction in hospital visits - Patients felt more control over their disease and reassured that a nurse was monitoring their health. | NR |
| Jehn 2013(34)  Germany | RHM: 32  no RHM: 30 | NR | NR | NR | NR |
| Jodar-Sanchez 2013(35)  Spain | RHM: 24  no RHM: 21 | - Survey was developed to seek satisfaction with the telehealth program. - Four questions on a 4- point rating scale ranging from very dissatisfied to very satisfied. - Seven questions on a 5-point rating scale ranging from strongly disagree to strongly agree. | **Satisfaction with explanations about the operation and maintenance of equipment given by staff – n(%satisfied)*:**  RHM(n=23): 21 (91)  **Satisfaction with telephone support– n(%satisfied)*:**  RHM(n=23): 22(96)  **Satisfaction with the troubleshooting of clinical and technical problems through the telephone support- n(%satisfied)*:**  RHM(n=23): 19 (83)  **Technology is safe to operate- n(%agreed)*:**  RHM(n=23): 15(65)  **Technology is easy to use- n(%agreed)*:**  RHM(n=23): 21(91) | **Intervention helped coping better with symptoms- n(%agreed)*:**  RHM(n=23): 15(65)  **Intervention helped understanding the illness better- n(%agreed)*:**  RHM(n=23): 14(61)  **Intervention helped reduce anxiety levels-** **n(%agreed)*:**  RHM(n=23): 18(78)  **Intervention improved autonomy and reduced visits to health centres or hospitals- n(%agreed)*:**  RHM(n=23): 11(48)  **Intervention had positive impact on coping with illness- n(%agreed)*:**  RHM(n=23): 20 (87)  **Intervention benefited family members in reducing burden due to disease-n(%agreed)*:**  RHM(n=23): 17(74) | NR |
| Pare 2013(36)  Canada | RHM: 60  no RHM: 60 | - A survey was developed. Nine questions on a 5- point rating scale ranging from not at all to enormously. | **Technology is easy to use- Mean±SD:**  RHM: 4.2±0.9  **Technology is user-friendly- Mean±SD:**  RHM: 3.9±1.0  **The automated system is easy to understand- Mean±SD:**  RHM: 4.0±0.9  **The automated system worked well- mean±SD:**  RHM: 3.9±1.0  **The information from intervention is complete- mean±SD:**  RHM: 3.9±0.8 | **Intervention met personal needs- mean±SD:**  RHM: 3.9±1.0  **Intervention helped managing care- mean±SD:**  RHM: 4.0±0.8 | **Overall satisfaction- mean±SD:**  RHM: 4.2±0.9  **Wish to continue with RHM- n(%yes):**  RHM: 51(86) |
| Chau 2012(37)  Hong Kong | RHM: 30  no RHM: 23 | - Patient satisfaction was measured by a self-developed 10-item questionnaire. - Each item was rated on a 1-5 Likert scale, in which higher scores indicated higher levels of satisfaction. - Questionnaire also included three open-ended questions to identify the patients’ perception of the telecare system | **Understanding of the use of telecare equipment is adequate- mean±SD:**  RHM(n=22): 3.5±1.1  **Equipment is difficult to operate mean±SD:**  RHM(n=22): 2.4±0.8  Five patients commented that they did not know how to fully operate the device.  Some participants stated that the phone display was too small to operate and read.  Quite a few participants experienced problems in using the ‘belt’ to measure their respiratory rate.  Participants had issues to recharge the device.  **Satisfaction with the medication prompt- mean±SD:**  RHM(n=22): 3.5±0.8  Over 60% of patients were satisfied with the medication prompt  Some participants stated that they remembered to take their medications and managed their illness well, so the medication prompt was not very helpful to them.  **Automated health care advice is not necessary- mean±SD, n(%not necessary):**  RHM(n=22): 2.8±0.9, 11(50)  **Preference of technology to monitor condition at home- mean±SD:**  RHM(n=22): 3.6±0.8  Over 60% of patients preferred telecare service to monitor their respiratory condition at home  **Explanation for use of equipment was adequate- mean±SD, n(%satisfied):**  RHM(n=22): 3.9±0.6, 19(86) | **Support from nurse was helpful – mean±SD, n(%yes):**  RHM(n=22): 4.3±0.5, 22(100)  Nine patients reported that the nurse offering the telecare support was helpful in terms of timely care and prompt follow-up actions.  **Intervention is not useful in helping managing disease at home- mean±SD, n(%not useful):**  RHM(n=22): 2.8±1.0, 10 (45) | **Recommend to others- mean±SD:**  RHM(n=22):3.1±0.9  **Overall satisfaction- mean±SD, n(%satified):**  RHM(n=22):3.9±0.6, 20(91)  Overall, participants expressed satisfaction with the telecare service because it facilitated clinical monitoring of patients in their homes. |
| Dinesen 2012(38)  Denmark | RHM: 60  no RHM: 51 | NR | NR | NR | NR |
| Lewis 2010(39;44)  UK | RHM: 20  no RHM: 20 | - Patients completed a survey. No further details provided. | - None of the patients reported difficulties using technology | NR | **System was helpful- n(%):**  RHM(n=17):  Helpful: 15(88)  Neither agreed or disagree: 1 (6)  Not helpful: 1 (6) |
| Au 2015(40)  USA | RHM: 619  no RHM: 619 | NR | NR | NR | NR |
| Davis 2015(41)  USA | RHM: 69  no RHM: 174 | - Patients completed the Centura Telehealth Patient Satisfaction Survey. - Ten questions on a 5-point rating scale ranging from “no, definitely not” to “yes, definitely”. | **Explanation for the use of equipment was adequate- mean±SD:**  RHM: 5.0+0.1  **Equipment was easy to use- mean±SD:**  RHM: 5.0+0.1  **Uncomfortable using technology- mean±SD:**  RHM: 1.0+0.3  **Technology took too much time to use- mean±SD:**  RHM: 1.1+0.5  **Worries about privacy- mean±SD:**  RHM: 1.0±0.3 | **Intervention motivated health monitoring- mean±SD:**  RHM: 4.6+0.8  **Intervention improved health- mean±SD:**  RHM: 4.4+0.9  **Intervention improved involvement with patient’s healthcare:**  RHM: 4.4+1.0  **Care received with intervention was as good as having nurse visiting home:**  RHM: 4.4+1.0 | **Recommend to others- mean±SD:**  RHM: 4.9±0.4 |
| ***RHM with feedback vs RHM with no feedback*** | | | | | |
| Sink 2018(42)  USA | RHM: 83  no RHM: 85 | NR | NR | NR | NR |
| Franke 2016(43)  Germany | Total: 53 | NR | NR | NR | NR |
| Notes: Tabak 2014a(28) and Tabak 2014b(29) used the same exercise monitoring device and smartphone technology. De San Miguel 2013 (33) and Lewis 2010 (39;44) used the same telemonitoring device. Segrelles 2014 (32) and Jodar-Sanchez 2013 (35) used the same devices to collect vital signs measures and modem technology to transmit collected measurements.  *Number is based on patients who reported “strongly agree” and “agree” or “satisfied” and “very satisfied”.  NR: not reported; RHM: remote home monitoring; SD: standard deviation; SE: standard error; SUS: system usability scale | | | | | |

| Table S12. Frequency of exacerbations, Hospital admissions, ER visits and physician visits | | | | | | | | | |
| --- | --- | --- | --- | --- | --- | --- | --- | --- | --- |
| **Study** | **Sample size** | **Follow-up time** | **Number of exacerbations** | **Hospital admission due to COPD** | **Time to hospitalization due to COPD in days**  **Mean±SD**  **Median (IQR)** | **Hospital admission (all causes)** | **Length of hospitalization in days**  **Mean±SD**  **Median (IQR)** | **ER visits** | **Physician visits** |
| ***RHM (smartphones, apps, tablets) vs no RHM*** | | | | | | | | | |
| Park 2020(25)  South Korea | RHM: 23  no RHM: 21 | 6 months | **Exacerbations requiring ED, outpatient visit or hospitalization- n(%):**  RHM(n=22): 6 (27)  no RHM(n=20): 3 (15)  p=NR | **Number of patients- n(%):**  RHM(n=22): 2 (9)  no RHM(n=20): 2 (10)  p=1.00 | NR | NR | NR | **Due to COPD-n(%):**  RHM (n=22): 1 (5)  no RHM(n=20): 0 (0)  p=1.00 | **Due to COPD- n(%):**  RHM(n=22): 3 (14)  no RHM(n=20): 1(5)  p=0.61 |
| Boer 2019(26)  Netherlands | RHM: 43  no RHM: 44 | 12 months | **Symptom-based exacerbations- mean±SD:**  RHM(n=41): 4.5±2.3  no RHM: 4.3±2.1  RR (95%CI): 1.1 (0.6,1.7)  p=0.80  **Exacerbation requiring medication- mean±SD:**  RHM(n=41): 1.1±1.5  no RHM: 1.0±1.3  RR(95%CI): 1.0 (0.5, 1.9)  p=0.97  **Exacerbation requiring hospital admission- mean±SD:**  RHM(n=41): 0.1±0.4  no RHM: 0.1±0.4  RR(95%CI): 1.2 (0.3, 4.4)  p=0.74 | **Total:**  RHM(n=41): 6  no RHM: 5  **Mean±SD:**  RHM(n=41): 0.1±0.4  no RHM: 0.1±0.4  RR(95%CI): 1.2 (0.3, 4.4)  p=0.74 | NR | NR | NR | NR | **Due to COPD- mean±SD:**  RHM(n=41): 1.6±1.7  no RHM: 1.6±2.0  RR(95%CI): 0.9 (0.5, 1.7)  p=0.70 |
| Walker 2018 (27)  Spain, UK, Slovenia, Estonia, Sweden | RHM: 154  no RHM: 158 | 9 months | **Exacerbation requiring medication - Rate:**  RHM: 1.7  no RHM: 1.5  p=0.50 | **Number of patients- n(%):**  RHM: 45 (29)  no RHM: 41 (26)  p=0.34 | RHM: 224 (209-240)*  no RHM: 254 (240-270)*  p=0.34 | NR | **All:**  RHM: 1.0 (1.0-6.7)  no RHM: 4.0 (1.0-9.0)  p=0.04 | NR | NR |
| Tabak 2014a(28)  Netherlands | RHM: 15  no RHM: 14 | 9 months | **Exacerbation requiring medication – total, median(IQR):**  RHM(n=12): 33  2.0 (1.0-3.0)  no RHM: NR  p=NR | **Total:**  RHM(n=12): 4  no RHM(n=12): 5  p=NR | NR | **Total:**  RHM(n=12): 4  no RHM(n=12): 2  p=NR | **Due to COPD:**  RHM(n=12): 5.5(4.8-6.3)  no RHM(n=12): 7.0(6.0-7.0)  p=NR | **Due to COPD- total:**  RHM(n=12): 5  no RHM(n=12): 5  p=NR | NR |
| Tabak 2014b (29)  Netherlands | RHM: 18  no RHM: 16 | 1 month | NR | NR | NR | NR | NR | NR | NR |
| ***RHM (dedicated monitoring devices) vs no RHM*** | | | | | | | | | |
| Shany 2017(30)  Australia | RHM: 21  no RHM: 21 | 12 months | NR | **Total:**  RHM: 30  no RHM: 53  **Mean±SD:**  RHM: 1.4±1.4  no RHM: 2.5±2.1  IRR(95%CI): 0.8 (0.5, 1.2)  p=0.32 | RHM: 84 (210)  no RHM: 63 (209)  p=NR | NR | **Due to COPD:**  RHM: 6.7±8.1  no RHM: 9.6±10.5  p=NR | **Due to COPD- total:**  RHM: 38  no RHM: 62  **Mean±SD:**  RHM: 1.8±1.7  no RHM: 3.0±2.5  IRR(95%CI): 0.9 (0.6, 1.3)  p=0.86 | NR |
| Vianello 2016 (31)  Italy | RHM: 230  no RHM: 104 | 12 months | NR | **Incidence rate per year(95%CI):**  RHM: 0.7 (0.6, 0.9)  no RHM: 0.8 (0.7, 1.0)  IRR(95%CI): 0.9 (0.8, 1.0)  p= 0.16 | NR | **Incidence rate per year(95%CI):**  RHM: 1.2 (0.9, 1.2)  no RHM: 1.2 (1.0, 1.4)  IRR(95%CI): 0.9 (0.7, 1.0)  p=0.16 | **Due to COPD:**  RHM: 18.9±15.3  no RHM: 23.3±19.0  p=0.22  **All:**  RHM: 22.9±25.1  no RHM: 25.5±23.2  p=0.53 | **All, incidence rate per year(95%CI):**  RHM: 1.3 (1.1, 1.5)  no RHM: 1.4 (1.1, 1.6)  IRR(95%CI): 0.9 (0.8, 1.2)  p=0.58 | **Due to COPD (specialist)- incidence rate per year(95%CI):**  RHM: 1.4 (1.2, 1.6)  no RHM: 1.7 (1.5, 2.0)  IRR(95%CI): 0.8 (0.7, 1.0)  p=0.05 |
| Segrelles 2014(32)  Spain | RHM: 30  no RHM: 30 | 7 months | NR | **Total:**  RHM: 12  no RHM: 33  p=0.01 | RHM: 141.1±NR  no RHM: 77.3±NR  p=0.003 | NR | **Due to COPD:**  RHM: 105**  no RHM: 276**  p=0.02 | **Due to COPD- total:**  RHM: 20  no RHM: 57  p=0.001 | NR |
| De San Miguel 2013(33)  Australia | RHM: 40  no RHM: 40 | 6 months | NR | **Total:**  RHM(n=36): 8  no RHM(n=35): 17  **Mean±SD:**  RHM(n=36): 0.2 ±0.5  no RHM(n=35): 0.5±0.8  p>0.05 | NR | **Total:**  RHM(n=36): 16  no RHM(n=35): 26  **Mean±SD:**  RHM(n=36): 0.4±0.7  no RHM(n=35): 0.7±1.2  p>0.05 | **Due to COPD:**  RHM(n=36): 2.4±7.1  no RHM(n=35): 4.6±9.1  p>0.05  **All:**  RHM(n=36): 2.9±7.3  no RHM(n=35): 5.2±9.3  p>0.05 | **Due to COPD- total:**  RHM(n=36): 6  no RHM(n=35): 11  **Mean±SD:**  RHM(n=36): 0.17±0.51  no RHM(n=35): 0.31±0.63  p>0.05  **All- total:**  RHM(n=36): 18  no RHM(n=35): 21  **Mean±SD:**  RHM(n=36): 0.5±0.77  no RHM(n=35): 0.6±0.95  p>0.05 | **Due to COPD (specialist)- total:**  RHM(n=36): 60  no RHM(n=35): 55  **Mean±SD:**  RHM(n=36): 1.7±1.7  no RHM(n=35): 1.6±1.7  p>0.05  **All (specialist)- total:**  RHM(n=36): 95  no RHM(n=35): 96  **Mean±SD:**  RHM(n=36): 2.6±2.2  no RHM(n=35): 2.7±2.1  p>0.05  **Due to COPD (GP)- total:**  RHM(n=36): 35  no RHM(n=35): 33  **Mean±SD:**  RHM(n=36): 1.0±1.3  no RHM(n=35): 0.9±1.3  p>0.05  **All (GP)- total:**  RHM(n=36): 251  no RHM(n=35): 208  **Mean±SD:**  RHM(n=36): 7.0±5.1  no RHM(n=35): 5.9±4.4  p>0.05 |
| Jehn 2013(34)  Germany | RHM: 32  no RHM: 30 | 9 months | **Exacerbations requiring hospitalization- Total:**  RHM: 7  no RHM: 22  p=0.01 | **Total:**  RHM: 7  no RHM: 22  p=0.01 | NR | NR | **Due to COPD:**  RHM: 34**  no RHM: 97**  P<0.05 | NR | **Due to COPD (specialist)- total:**  RHM: 24  no RHM: 42  p=0.04  **Due to COPD (GP)- total:**  RHM: 9  no RHM: 11  p=0.76 |
| Jodar-Sanchez 2013(35)  Spain | RHM: 24  no RHM: 21 | 4 months | NR | NR | NR | **Unspecified cause of hospitalization- n(%):**  RHM: 5(21)  no RHM: 3(14)  **Mean±SD:**  RHM: 0.4±0.8  no RHM: 0.1±0.4  p=0.47 | **Unspecified:**  RHM: 4.4±12.2  no RHM: 1.4±4.0  p=0.50 | **Unspecified, n(%):**  RHM: 4(17)  no RHM: 7(33)  **Mean±SD:**  RHM: 0.4±0.7  no RHM: 0.3±0.7  p=0.25 | **Unspecified (specialist)-n(%):**  RHM: 4(17)  0.25±0.61  no RHM: 1 (5)  **Mean±SD:**  RHM: 0.2±0.6  no RHM: 0.0±0.2  p=0.20 |
| Pare 2013(36)  Canada | RHM: 60  no RHM: 60 | Pre-phase: 12 months  Post phase: 12 months | NR | **Number of patients in the 6 months with intervention- n(%):**  RHM: 17 (28)  no RHM: 15 (25)  p=NR  **Mean±SD:**  RHM: 0.4±0.8  no RHM: 0.5±1.0  p=NR  **Number of patients in the 6 months post intervention- n(%):**  RHM: 10 (17)  no RHM: 16 (27)  **Mean±SD:**  RHM: 0.4±1.0  no RHM: 0.6±1.2  p=NR | NR | NR | **6 months with intervention due to COPD:**  RHM: 6.5±16.1  no RHM: 3.5±9.6  p=NR  **6 months post intervention due to COPD:**  RHM: 4.3±8.9  no RHM: 3.5±9.6  P<0.01 | **6 months with intervention due to COPD-n(%):**  RHM: 18 (30)  no RHM: 18 (30)  **Mean±SD:**  RHM: 0.6±1.2  no RHM: 0.6±1.2  p=NR  **6 months post intervention due to COPD-n(%):**  RHM: 13 (22)  no RHM: 23 (38)  **Mean±SD:**  RHM: 0.7±1.6  no RHM: 1.2±2.1  p=0.34 | NR |
| Chau 2012(37)  Hong Kong | RHM: 30  no RHM: 23 | Mean  RHM: 65.2days  no RHM: 68.4 days | NR | **Number of patients-n(%):**  RHM: 7 (32)  no RHM: 3 (17)  p>0.05 | NR | NR | **Due to COPD:**  RHM: 2.2±4.7  no RHM: 0.8±1.9  p=NR | **Due to COPD-n(%):**  RHM: 7 (32)  no RHM: 3 (17)  p>0.05 | NR |
| Dinesen 2012(38)  Denmark | RHM: 60  no RHM: 51 | 10 months | NR | NR | NR | **Rate (95%CI):**  RHM: 0.5 (0.3, 0.7)  no RHM: 1.2 (0.6, 1.7)  p=0.04 | NR | NR | NR |
| Lewis 2010(39;44)  UK | RHM: 20  no RHM: 20 | 12 months | NR | **Median(IQR) number of patients in the 6 months with intervention:**  RHM: 0.0 (0.0- 0.0)  no RHM: 0.0 (0.0- 0.7)  p=0.16  **Median(IQR) number of patients in the 6 months post intervention:**  RHM: 0.0 (0.0*,* 1*.*0)  no RHM: 0.0 (0.0*,* 1*.*0)  p=0.54 | NR | NR | **In the 6 months with intervention due to COPD:**  RHM: 0.0 (0.0*,* 0.0)  no RHM: 0.0 (0.0*,* 1*.*5)  p=0.66  **In the 6 months post intervention due to COPD:**  RHM: 0.0 (0.0*,* 2*.*0)  no RHM: 0.0 (0.0*,* 3*.*8)  p=0.62 | **In the 6 months with intervention due to COPD- median(IQR):**  RHM: 0.0 (0.0*,* 0*.*8)  no RHM: 0.0 (0.0*,* 1*.*0)  p=0.24  **In the 6 months post intervention due to COPD- median(IQR):**  RHM; 0.0 (0.0*,* 1*.*5)  no RHM: 0.0 (0.0*,* 1*.*0)  p=0.26 | **In the 6 months with intervention due to COPD (GP)- median(IQR):**  RHM: 2.0 (1.0*,* 3*.*8)  no RHM: 4.0 (2.0*,* 6.0)  p=0.03  **In the 6 months post intervention due to COPD (GP)- median(IQR):**  RHM: 3.0 (2*.*0*, .*3*.*0)  no RHM: 3.0 (2*.*0*,* 4*.*5)  p=0.51  **In the 6 months with intervention, all(GP)- median(IQR):**  RHM: 1.0 (0.0*,* 2.0)  no RHM: 1.0 (1.0*,* 3.0)  p=0.23  **In the 6 months post intervention , all (GP)- median(IQR):**  RHM: 2.0 (0.0*,* 3*.*0)  no RHM: 3.0 (1*.*5*,* 5*.*0)  p=0.23 |
| Au 2015(40)  USA | RHM: 619  no RHM: 619 | 3 years | **Exacerbations requiring hospitalization- mean per quarter:**  RHM: 0.03   - Engaged†: 0.03 - Nonengaged†: 0.04   no RHM: 0.04  MD(95%CI):-0.01 (-0.03, 0.01)  p=0.34   - Engaged†:   MD(95%CI):--0.03 (-0.07, 0.00)  p=0.05   - Nonengaged†:   MD(95%CI):-0.01 (-0.02, 0.03)  p=0.60 | **Mean per quarter:**  RHM: 0.09   - Engaged†: 0.08 - Nonengaged†: 0.10   no RHM: 0.14  MD(95%CI):--0.06 (-0.09, -0.03)  P<0.01   - Engaged†:   MD(95%CI):--0.08 (-0.12, -0.03)  P<0.01   - Nonengaged†:   MD(95%CI):--0.05 (-0.09, -0.01)  p=0.03 | NR | **Mean per quarter:**  RHM: 0.18   - Engaged†: 0.16 - Nonengaged†: 0.20   no RHM: 0.20  MD(95%CI):-0.05 (-0.09, -0.01)  P<0.01   - Engaged†:   MD(95%CI):-0.07(-0.14, -0.02)  P<0.01   - Nonengaged†:   MD(95%CI):-0.03 (-0.08, 0.02)  p=0.26 | NR | **All-mean per quarter:**  RHM: 0.34   - Engaged†: 0.27 - Nonengaged†: 0.39   no RHM: 0.35  MD(95%CI):0.00 (-0.05, 0.07)  p=0.79   - Engaged†:   MD(95%CI): -0.02 (-0.10, 0.07)  p=0.76   - Nonengaged†:   MD(95%CI):0.02 (-0.06, 0.11)  p=0.50 | NR |
| Davis 2015(41)  USA | RHM: 58  no RHM: 174 | 3 months | NR | NR | NR | NR | NR | **All in 1 month - n(%):**  RHM: 4 (7)  no RHM: 19 (11)  OR(95%CI): 0.6 (0.2, 1.9)  p=0.15  **All in 3 months- n(%):**  RHM: 12 (21)  no RHM: 39 (22)  OR(95%CI): 0.9 (0.4, 1.9)  p=0.15  **All in 6 months- n(%):**  RHM: 18 (31)  no RHM: 55 (32)  OR(95%CI): 1.0 (0.5, 1.8)  p=0.13 | NR |
| ***RHM with feedback vs RHM with no feedback*** | | | | | | | | | |
| Sink 2018(42)  USA | RHM: 83  no RHM: 85 | 8 months | NR | **Total:**  RHM: 6  no RHM: 16  P<0.05 | RHM: NR  no RHM: NR  HR(95%CI): 2.4 (1.0, 5.4)  p=0.04 | NR | NR | NR | NR |
| Franke 2016(43)  Germany | Total: 53 | 6 months | NR | NR | NR | NR | NR | NR | NR |
| Notes: Tabak 2014a(28) and Tabak 2014b(29) used the same exercise monitoring device and smartphone technology. De San Miguel 2013 (33) and Lewis 2010 (39;44) used the same telemonitoring device. Segrelles 2014 (32) and Jodar-Sanchez 2013 (35) used the same devices to collect vital signs measures and modem technology to transmit collected measurements.  *Reported as mean (IQR).  ** Reported as total number.  † Patients were classified as “ engaged” if they used intervention technology at least once during the study. “Nonengaged” were patients who never used technology.  CI: confidence interval; COPD: chronic obstructive pulmonary disease; ER: emergency room; GP: general practitioner; HR: hazard ratio; IQR: interquartile range; IRR: incidence rate ratio; MD: mean difference; NA: not applicable; NR: not reported; OR: odds ratio; RR: risk ratio; RHM: remote home monitoring; SD: standard deviation; SEM: standard error of mean | | | | | | | | | |

| **Table S13. Adverse events and deaths during the follow-up period** | | | | |
| --- | --- | --- | --- | --- |
| **Study** | **Sample size** | **Adverse events n (%)** | **Deaths due to COPD n (%)** | **Deaths from all causes n (%)** |
| ***RHM (smartphones, apps, tablets) vs no RHM*** | | | | |
| Park 2020  *South Korea* | RHM: 23  no RHM: 21 | **6 months:** RHM: 0 (0) no RHM: 0 (0) p=NA | NR | NR |
| Boer 2019  *Netherlands* | RHM: 43  no RHM: 44 | NR | NR | **12 months (unspecified):** RHM: 2 (5)  no RHM: 0 (0) p=NR |
| Walker 2018  *Spain, UK, Slovenia, Estonia, Sweden* | RHM: 154  no RHM: 158 | NR | NR | **9 months (unspecified):** RHM: 3 (2)  no RHM: 4 (3) p=NR |
| Tabak 2014a  *Netherlands* | RHM: 15  no RHM: 14 | NR | NR | NR |
| Tabak 2014b  *Netherlands* | RHM: 18  no RHM: 16 | NR | NR | NR |
| ***RHM (dedicated monitoring devices) vs no RHM*** | | | | |
| Shany 2017  *Australia* | RHM: 21  no RHM: 21 | NR | **12 months:** RHM: 2 (10)  no RHM: 2 (10) p=NR | **12 months:** RHM: 3(14) no RHM: 3(14) p=NR |
| Vianello 2016  *Italy* | RHM: 230  no RHM: 104 | NR | **12 months:** RHM: 23(10)  no RHM: 9 (9)  RR(95%CI): 1.2 (0.6, 2.4) p=0.85 | **12 months:** RHM: 26(11) no RHM: 13(13) p=NR |
| Segrelles 2014  *Spain* | RHM: 30  no RHM: 30 | NR | **7 months:** RHM: 1 (3) no RHM: 3 (10)  p=NR | **7 months:** RHM: 2 (7) no RHM: 4 (13)  p=NR |
| De San Miguel 2013  *Australia* | RHM: 40  no RHM: 40 | NR | NR | NR |
| Jehn 2013  Germany | RHM: 32  no RHM: 30 | NR | NR | NR |
| Jodar-Sanchez 2013  *Spain* | RHM: 24  no RHM: 21 | NR | NR | **4 months (unspecified):** RHM: 1(4)  no RHM: 1(5) p=NR |
| Pare 2013  *Canada* | RHM: 60  no RHM: 60 | NR | NR | NR |
| Chau 2012  *Hong Kong* | RHM: 30  no RHM: 23 | NR | NR | NR |
| Dinesen 2012  *Denmark* | RHM: 60  no RHM: 51 | NR | NR | NR |
| Lewis 2010  *UK* | RHM: 20  no RHM: 20 | NR | NR | **12 months:** RHM: 2(10) no RHM: 0(0)  p=NR |
| Au 2015  *USA* | RHM: 619  no RHM: 619 | NR | NR | NR |
| Davis 2015  *USA* | RHM: 69  no RHM: 174 | NR | NR | **3 months (unspecified):** RHM: 2(3)  no RHM: 0(0) p=NR |
| ***RHM with feedback ( Phone calls, text messages) vs RHM with no feedback*** | | | | |
| Sink 2018  *USA* | RHM: 83  no RHM: 85 | NR | NR | NR |
| Franke 2016  *Germany* | Total: 53 | NR | NR | NR |
| Notes: Tabak 2014a and Tabak 2014b used the same exercise monitoring device and smartphone technology. De San Miguel 2013 and Lewis 2010 used the same telemonitoring device. Segrelles 2014 and Jodar-Sanchez 2013 used the same devices to collect vital signs measures and modem technology to transmit collected measurements.  COPD: Chronic Obstructive Pulmonary Disease; NA: not applicable; NR: not reported; RHM: remote home monitoring | | | | |

| Table S14. Exercise capacity and activity levels | | | | | | | | |
| --- | --- | --- | --- | --- | --- | --- | --- | --- |
| **Study** | **Sample size** | **Time** | **6MWT/ 6MWD in meters** | | | **Physical activity levels** | | |
|  |  |  | **RHM**  **Mean±SD** | **Comparison group**  **Mean±SD** | **Diff. between groups**  **MD(95%CI)** | **RHM**  **Mean±SD** | **Comparison group**  **Mean±SD** | **Diff. between groups**  **MD(95%CI)** |
| ***RHM (smartphones, apps, tablets) vs no RHM*** | | | | | | | | |
| Park 2020(25)  South Korea | RHM: 23  no RHM: 21 | Baseline | RHM(n=23): 378.3±97.0 | no RHM(n=20): 398.1± 78.7 | 0.48 | **Total activity count (per wear time):**  RHM(n=22): 215.6±103.2  **Daily steps:**  RHM(n=22): 5223.7±2899.6 | **Total activity count (per wear time):**  no RHM(n=20): 258.8±105.7  **Daily steps:**  no RHM(n=20): 6756.3±2978.8 | **Total activity count (per wear time):**  p=0.19  **Daily steps:**  p=0.10 |
|  |  | 6 months  *Diff. within group* | RHM(n=23): 433.2±107.2  *P<0.05* | no RHM(n=20): 437.6±83.6  *p>0.05* | NR  p=0.63 | **Total activity count (per wear time):**  RHM(n=22): 275.1±99.8  *P<0.05*  **Daily steps:**  RHM(n=22): 6546.8±2354.4  *P<0.05* | **Total activity count (per wear time):**  no RHM(n=20): 258.6±111.5  *p>0.05*  **Daily steps:**  no RHM(n=20): 6890.4±2967.7  *p>0.05* | **Total activity count (per wear time):**  NR  p=0.01  **Daily steps:**  NR  p=0.06 |
| Boer 2019(26)  Netherlands | RHM: 43  no RHM: 44 | NR | NR | NR | NR | NR | NR | NR |
| Walker 2018 (27)  Spain, UK, Slovenia, Estonia, Sweden | RHM: 154  no RHM: 158 | NR | NR | NR | NR | NR | NR | NR |
| Tabak 2014a(28)  Netherlands | RHM: 15  no RHM: 14 | Baseline | RHM(n=12): 409±29.5 | no RHM(n=12): 300.1±33.6 | NR | **BPAQ:**  RHM(n=11): 7.1±0.4 | **BPAQ:**  no RHM(n=11): 6.1±0.4 | NR |
|  |  | 1 month | NR | NR | NR | **BPAQ:**  RHM(n=11): 6.5±0.4 | **BPAQ:**  no RHM(n=11): 6.7±0.4 | NR |
|  |  | 3 months | RHM( n=11): 412±38.7 | no RHM(n=9): 312.4±44.0 | NR | **BPAQ:**  RHM(n=11): 7.2±0.4 | **BPAQ:**  no RHM(n=11): 6.2±0.4 | NR |
| Tabak 2014b (29)  Netherlands | RHM: 18  no RHM: 16 | Baseline | NR | NR | NR | **Daily steps:**  RHM(n=13): 5766±965 | **Daily steps:**  no RHM: 5256±865 | NR |
|  |  | 1 month | NR | NR | NR | **Daily steps:**  RHM(n=13): 5603±964 | **Daily steps:**  no RHM: 4617±865 | NR  p=0.38 |
| ***RHM (dedicated monitoring devices) vs no RHM*** | | | | | | | | |
| Shany 2016(30)  Australia | RHM: 21  no RHM: 21 | NR | NR | NR | NR | NR | NR | NR |
| Vianello 2016 (31)  Italy | RHM: 230  no RHM: 104 | NR | NR | NR | NR | NR | NR | NR |
| Segrelles 2014(32)  Spain | RHM: 30  no RHM: 30 | NR | NR | NR | NR | NR | NR | NR |
| De San Miguel 2013(33)  Australia | RHM: 40  no RHM: 40 | NR | NR | NR | NR | NR | NR | NR |
| Jehn 2013(34)  Germany | RHM: 32  no RHM: 30 | Baseline | RHM: 377.0±88.0 | no RHM: 377.0±78.0 | NR | NR | NR | NR |
|  |  | 9 months  *Diff. within group*  *MD±SD* | RHM(n=27): 464.0±60.5  *87.0±65.7*  *p=0.01* | no RHM(n=25): 400.0±72.4  *23.9±70.3*  *p=0.23* | NR | NR | NR | NR |
| Jodar-Sanchez 2013(35)  Spain | RHM: 24  no RHM: 21 | NR | NR | NR | NR | NR | NR | NR |
| Pare 2013(36)  Canada | RHM: 60  no RHM: 60 | NR | NR | NR | NR | NR | NR | NR |
| Chau 2012(37)  Hong Kong | RHM: 30  no RHM: 23 | NR | NR | NR | NR | NR | NR | NR |
| Dinesen 2012(38)  Denmark | RHM: 60  no RHM: 51 | NR | NR | NR | NR | NR | NR | NR |
| Lewis 2010(39;44)  UK | RHM: 20  no RHM: 20 | NR | NR | NR | NR | NR | NR | NR |
| Au 2015(40)  USA | RHM: 619  no RHM: 619 | NR | NR | NR | NR | NR | NR | NR |
| Davis 2015(41)  USA | RHM: 69  no RHM: 174 | NR | NR | NR | NR | NR | NR | NR |
| ***RHM with feedback vs RHM with no feedback*** | | | | | | | | |
| Sink 2018(42)  USA | RHM: 83  no RHM: 85 | NR | NR | NR | NR | NR | NR | NR |
| Franke 2016(43)  Germany | Total: 53 | Baseline | Total(n=44)*: 384.3±102.6 | | NR | **Godin total leisure activity:**  Total(n=44)*: 12.2±12.1 | | p=NA |
|  |  | 6 months | NR | NR | NR | **Godin total leisure activity:**  RHM phase(n=44)*: 36.3±16.3 | **Godin total leisure activity:**  no RHM phase(n=44)*: 33.7±17.3 | **Godin total leisure activity:**  NR  p<0.001 |
| Notes: Tabak 2014a(28) and Tabak 2014b(29) used the same exercise monitoring device and smartphone technology. De San Miguel 2013 (33) and Lewis 2010 (39;44) used the same telemonitoring device. Segrelles 2014 (32) and Jodar-Sanchez 2013 (35) used the same devices to collect vital signs measures and modem technology to transmit collected measurements.  *Cross-over randomized trial.  6MWD: 6 minutes walk distance; 6MWT: 6 minutes walk test; BPAQ: Baecke physical activity questionnaire; CI: confidence interval; MD: mean difference; NA: not applicable; NR: not reported; RHM: remote home monitoring; SD: standard deviation | | | | | | | | |

| Table S15. Mental health | | | | | | | | | | | |
| --- | --- | --- | --- | --- | --- | --- | --- | --- | --- | --- | --- |
| **Study** | **Sample size** | **Time** | **HADS anxiety** | | | **HADS depression** | | | **Other tools** | | |
|  |  |  | **RHM**  **Mean±SD** | **Comparison group**  **Mean±SD** | **Diff. between groups**  **MD(95%CI)** | **RHM**  **Mean±SD** | **Comparison group**  **Mean±SD** | **Diff. between groups**  **MD(95%CI)** | **RHM**  **Mean±SD** | **Comparison group**  **Mean±SD** | **Diff. between groups**  **MD(95%CI)** |
| ***RHM (smartphones, apps, tablets) vs no RHM*** | | | | | | | | | | | |
| Park 2020(25)  South Korea | RHM: 23  no RHM: 21 | Baseline | NR | NR | NR | NR | NR | NR | **Tension-anxiety (POMS):**  RHM(n=22): 4.9±2.6  **Depression (POMS):**  RHM(n=22): 3.5± 2.7 | **Tension-anxiety (POMS):**  no RHM(n=20): 5.7± 4.3  **Depression (POMS):**  no RHM(n=20): 5.2± 5.5 | **Tension-anxiety (POMS):**  p=0.42  **Depression (POMS):**  p=0.21 |
|  |  | 6 months  *Diff within group* | NR | NR | NR | NR | NR | NR | **Tension-anxiety (POMS):**  RHM(n=22): 5.2± 3.2  *p>0.05*  **Depression (POMS):**  RHM(n=22): 3.7± 3.3  *p>0.05* | **Tension-anxiety (POMS):**  no RHM(n=20): 5.8±4.6  *p>0.05*  **Depression (POMS):**  no RHM(n=20): 5.4± 6.9  *p>0.05* | **Tension-anxiety (POMS):**  NR  p=0.73  **Depression (POMS):**  NR  p=0.91 |
| Boer 2019(26)  Netherlands | RHM: 43  no RHM: 44 | NR | NR | NR | NR | NR | NR | NR | NR | NR | NR |
| Walker 2018 (27)  Spain, UK, Slovenia, Estonia, Sweden | RHM: 154  no RHM: 158 | Baseline | NR | NR | NR | NR | NR | NR | **Depression (PHQ-9):**  RHM: 6.3±5.7 | **Depression (PHQ-9):**  no RHM: 6.0±5.8 | NR |
|  |  | 8 months | NR | NR | NR | NR | NR | NR | **Depression (PHQ-9):**  RHM(n=150): 6.7±5.9 | **Depression (PHQ-9):**  no RHM(n=155): 6.3±5.4 | NR  p=0.61 |
| Tabak 2014a(28)  Netherlands | RHM: 15  no RHM: 14 | NR | NR | NR | NR | NR | NR | NR | NR | NR | NR |
| Tabak 2014b (29)  Netherlands | RHM: 18  no RHM: 16 | NR | NR | NR | NR | NR | NR | NR | NR | NR | NR |
| ***RHM (dedicated monitoring devices) vs no RHM*** | | | | | | | | | | | |
| Shany 2017(30)  Australia | RHM: 21  no RHM: 21 | Baseline | RHM: 7.8+4.7 | no RHM: 6.2+4.0 | p>0.05 | RHM: 6.0+3.0 | no RHM: 6.4+4.5 | p>0.05 | NR | NR | NR |
|  |  | 12 months | NR | NR | NR  p>0.05 | NR | NR | NR  p>0.05 | NR | NR | NR |
| Vianello 2016 (31)  Italy | RHM: 230  no RHM: 104 | Baseline | RHM: 4.7±3.4 | no RHM: 5.4±3.3 | p=0.09 | RHM: 5.1±4.4 | no RHM: 5.5±4.5 | p=0.60 | NR | NR | NR |
|  |  | 12 months  *Diff. within group*  *MD±SD* | RHM(n=181): 5.5±3.5  *0.85±3.68* | no RHM: 6.0±3.5  *0.62±3.6* | 0.2 (-0.7, 1.2)  p=0.65 | RHM(n=181): 5.6±4.4  *0.5±4.3* | no RHM: 6.2±4.2  *0.7±4.5* | -0.2 (-1.4, 0.9)  p=0.71 | NR | NR | NR |
| Segrelles 2014(32)  Spain | RHM: 30  no RHM: 30 | NR | NR | NR | NR | NR | NR | NR | NR | NR | NR |
| De San Miguel 2013(33)  Australia | RHM: 40  no RHM: 40 | NR | NR | NR | NR | NR | NR | NR | NR | NR | NR |
| Jehn 2013(34)  Germany | RHM: 32  no RHM: 30 | NR | NR | NR | NR | NR | NR | NR | NR | NR | NR |
| Jodar-Sanchez 2013(35)  Spain | RHM: 24  no RHM: 21 | NR | NR | NR | NR | NR | NR | NR | NR | NR | NR |
| Pare 2013(36)  Canada | RHM: 60  no RHM: 60 | NR | NR | NR | NR | NR | NR | NR | NR | NR | NR |
| Chau 2012(37)  Hong Kong | RHM: 30  no RHM: 23 | NR | NR | NR | NR | NR | NR | NR | NR | NR | NR |
| Dinesen 2012(38)  Denmark | RHM: 60  no RHM: 51 | NR | NR | NR | NR | NR | NR | NR | NR | NR | NR |
| Lewis 2010(39;44)  UK | RHM: 20  no RHM: 20 | Baseline | RHM: 5*.*6±3*.*5 | no RHM: 6*.*3±3*.*5 | p=0.56 | RHM: 6*.*3±3*.*5 | no RHM: 5*.*9±2*.*8 | p=0.70 | NR | NR | NR |
|  |  | 12 months | NR | NR | NR  p=0.83 | NR | NR | NR  p=0.70 | NR | NR | NR |
| Au 2015(40)  USA | RHM: 619  no RHM: 619 | NR | NR | NR | NR | NR | NR | NR | NR | NR | NR |
| Davis 2015(41)  USA | RHM: 58  no RHM: 174 | NR | NR | NR | NR | NR | NR | NR | NR | NR | NR |
| ***RHM with feedback vs RHM with no feedback*** | | | | | | | | | | | |
| Sink 2018(42)  USA | RHM: 83  no RHM: 85 | NR | NR | NR | NR | NR | NR | NR | NR | NR | NR |
| Franke 2016(43)  Germany | Total: 53 | NR | NR | NR | NR | NR | NR | NR | NR | NR | NR |
| Notes: Tabak 2014a(28) and Tabak 2014b(29) used the same exercise monitoring device and smartphone technology. De San Miguel 2013 (33) and Lewis 2010 (39;44) used the same telemonitoring device. Segrelles 2014 (32) and Jodar-Sanchez 2013 (35) used the same devices to collect vital signs measures and modem technology to transmit collected measurements.  CI: confidence interval; HADS: Hospital Anxiety and Depression Scale; MD: mean difference; NR: not reported; PHQ-9: Patient Health Questionnaire-9; POMS: Profile of Mood States-Short Form; RHM: remote home monitoring; SD: standard deviation | | | | | | | | | | | |

| Table S16. Self-efficacy | | | | | |
| --- | --- | --- | --- | --- | --- |
| **Study** | **Sample size** | **Time** | **Self-efficacy** | | |
|  |  |  | **RHM**  **Mean±SD** | **Comparison group**  **Mean±SD** | **Diff. between groups**  **MD(95%CI)** |
| ***RHM (smartphones, apps, tablets) vs no RHM*** | | | | | |
| Park 2020(25)  South Korea | RHM: 23  no RHM: 21 | Baseline | **Total (SEMCD):**  RHM(n=22): 6.7±1.9  **Managing dyspnea** **(SEMCD):**  RHM(n=22): 6.6± 2.2  **Managing exacerbation (SEMCD):**  RHM(n=22): 6.7±1.9  **Managing exercise (SEMCD):**  RHM(n=22): 7.4±1.5  **Increasing physical activity (SEMCD):**  RHM(n=22): 6.9±2.1  **Decreasing sedentary time (SEMCD):**  RHM(n=22): 7.2± 1.8 | **Total (SEMCD):**  no RHM(n=20): 6.5± 1.6  **Managing dyspnea (SEMCD):**  no RHM(n=20): 6.4± 2.1  **Managing exacerbation (SEMCD):**  no RHM(n=20): 6.2± 2.2  **Managing exercise (SEMCD):**  no RHM(n=20): 6.9±2.0  **Increasing physical activity (SEMCD):**  no RHM(n=20): 6.9±1.7  **Decreasing sedentary time (SEMCD):**  no RHM(n=20): 6.6±2.1 | **Total (SEMCD):**  p=0.66  **Managing dyspnea (SEMCD):**  p=0.78  **Managing exacerbation (SEMCD):**  p=0.46  **Managing exercise (SEMCD):**  p=0.32  **Increasing physical activity (SEMCD):**  p=0.99  **Decreasing sedentary time (SEMCD):**  p=0.18 |
|  |  | 6 months  *Diff. within group* | **Total (SEMCD):**  RHM(n=22): 6.9±1.7  *p>0.05*  **Managing dyspnea (SEMCD):**  RHM(n=22): 6.7±2.1  *p>0.05*  **Managing exacerbation (SEMCD):**  RHM(n=22): 6.9±2.0  *p>0.05*  **Managing exercise (SEMCD):**  RHM(n=22): 7.7±1.3  *p<0.05*  **Increasing physical activity (SEMCD):**  RHM(n=22): 7.9±1.7  *p<0.05*  **Decreasing sedentary time (SEMCD):**  RHM(n=22): 7.7±1.4  *p<0.05* | **Total (SEMCD):**  no RHM(n=20): 6.7±2.3  *p>0.05*  **Managing dyspnea (SEMCD):**  no RHM(n=20): 6.8±2.1  *p>0.05*  **Managing exacerbation (SEMCD):**  no RHM(n=20): 6.7±2.0  *p>0.05*  **Managing exercise (SEMCD):**  no RHM(n=20): 6.7± 2.3  *p>0.05*  **Increasing physical activity (SEMCD):**  no RHM(n=20): 6.7± 2.1  *p>0.05*  **Decreasing sedentary time (SEMCD):**  no RHM(n=20): 7.0± 1.8  *p>0.05* | **Total (SEMCD):**  NR  p=0.93  **Managing dyspnea (SEMCD):**  NR  p=0.53  **Managing exacerbation (SEMCD):**  NR  p=0.67  **Managing exercise (SEMCD):**  NR  p=0.46  **Increasing physical activity (SEMCD):**  NR  p=0.06  **Decreasing sedentary time (SEMCD):**  NR  p=0.86 |
| Boer 2019(26)  Netherlands | RHM: 43  no RHM: 44 | Baseline | **Exacerbation-related self-efficacy***  RHM: 2.9±0.4 | **Exacerbation-related self-efficacy***  no RHM: 2.8±0.4 | NR |
|  |  | 12 months | **Exacerbation-related self-efficacy***  RHM(n=35): 3.0±0.4 | **Exacerbation-related self-efficacy***  no RHM: 2.9±0.5 | **Exacerbation-related self-efficacy***  0.0 (-0.2, 0.2)  p=0.91 |
| Walker 2018 (27)  Spain, UK, Slovenia, Estonia, Sweden | RHM: 154  no RHM: 158 | NR | NR | NR | NR |
| Tabak 2014a(28)  Netherlands | RHM: 15  no RHM: 14 | NR | NR | NR | NR |
| Tabak 2014b (29)  Netherlands | RHM: 18  no RHM: 16 | NR | NR | NR | NR |
| ***RHM (dedicated monitoring devices) vs no RHM*** | | | | | |
| Shany 2017(30)  Australia | RHM: 21  no RHM: 21 | NR | NR | NR | NR |
| Vianello 2016 (31)  Italy | RHM: 230  no RHM: 104 | NR | NR | NR | NR |
| Segrelles 2014(32)  Spain | RHM: 30  no RHM: 30 | NR | NR | NR | NR |
| De San Miguel 2013(33)  Australia | RHM: 40  no RHM: 40 | NR | NR | NR | NR |
| Jehn 2013(34)  Germany | RHM: 32  no RHM: 30 | NR | NR | NR | NR |
| Jodar-Sanchez 2013(35)  Spain | RHM: 24  no RHM: 21 | NR | NR | NR | NR |
| Pare 2013(36)  Canada | RHM: 60  no RHM: 60 | NR | NR | NR | NR |
| Chau 2012(37)  Hong Kong | RHM: 30  no RHM: 23 | NR | NR | NR | NR |
| Dinesen 2012(38)  Denmark | RHM: 60  no RHM: 51 | NR | NR | NR | NR |
| Lewis 2010(39;44)  UK | RHM: 20  no RHM: 20 | NR | NR | NR | NR |
| Au 2015(40)  USA | RHM: 619  no RHM: 619 | NR | NR | NR | NR |
| Davis 2015(41)  USA | RHM: 69  no RHM: 174 | NR | NR | NR | NR |
| ***RHM(phone calls, text messages) vs no RHM*** | | | | | |
| Sink 2018(42)  USA | RHM: 83  no RHM: 85 | NR | NR | NR | NR |
| Franke 2016(43)  Germany | Total: 53 | NR | NR | NR | NR |
| Notes: Tabak 2014a(28) and Tabak 2014b(29) used the same exercise monitoring device and smartphone technology. De San Miguel 2013 (33) and Lewis 2010 (39;44) used the same telemonitoring device. Segrelles 2014 (32) and Jodar-Sanchez 2013 (35) used the same devices to collect vital signs measures and modem technology to transmit collected measurements.  *Study reported on the reliability of tool but not on the validity of the tool. Higher scores indicate better self-efficacy.  CI: confidence interval; MD: mean difference; NR: not reported; RHM: remote home monitoring; SD: standard deviation; SEMCD: Self-efficacy for Managing Chronic Disease 6-item Scale | | | | | |

| Table S17. Cost per patient | | | | | |
| --- | --- | --- | --- | --- | --- |
| **Study** | **Sample size** | **Study methods** | **Costs** | **Outcome measure** | **Findings**  **Mean±SD**  **Mean(95%CI)** |
| ***RHM (smartphones, apps, tablets) vs no RHM*** | | | | | |
| Park 2020(25)  South Korea | RHM: 23  no RHM: 21 | NR | NR | NR | NR |
| Boer 2019(26)  Netherlands | RHM: 43  no RHM: 44 | NR | NR | NR | NR |
| Walker 2018 (27)  Spain, UK, Slovenia, Estonia, Sweden | RHM: 154  no RHM: 158 | *Study type:*  Cost-utility analysis  *Patient population:*  312 participants with moderate or higher COPD  *Analysis:*  Study done alongside a RCT  *Perspective:*  Payer  *Time horizon:*  9 months | *Costs included:*  Equipment and technical support: Not included  Hospital resources: hospitalizations, ER visits, early discharge (UK only), hospital-at-home (UK only), outpatient visits and use of ambulance  Primary care resources: consultations with the GP or nurse at the GP office, and contacts with health professionals  *Information source:*  Clinical records and participants self-reported health care utilization.  *Other:*  Costs reported in Euros (€) | QALYs | *Cost per patient:*  Overall:  RHM: €3,547±€5,038  no RHM: €4,831±€10,250  p=0.01  Patients with HF or IHD:  RHM: €4,237±€6,154  no RHM: €6,520±€12,575  p=0.01  Patients with severe or very severe COPD:  RHM: €4,362±€6,072  no RHM: €5,704±€10,717  p=0.12  Patients with frequent exacerbations:  RHM: €3,847±€5,778  no RHM: €5,798±€12,221  p=0.01  *QALYs*  Overall:  RHM: 0.491±0.164  no RHM: 0.485±0.142  p=0.73  Patients with HF or IHD:  RHM: 0.430±0.185  no RHM: 0.433±0.192  p=0.97  Patients with severe or very severe COPD:  RHM: 0.473±0.153  no RHM: 0.484±0.176  p=0.68  Patients with frequent exacerbations:  RHM: 0.450±0.145  no RHM: 0.457±0.191  p=0.84 |
| Tabak 2014a(28)  Netherlands | RHM: 15  no RHM: 14 | NR | NR | NR | NR |
| Tabak 2014b (29)  Netherlands | RHM: 18  no RHM: 16 | NR | NR | NR | NR |
| ***RHM (dedicated monitoring devices) vs no RHM*** | | | | | |
| Shany 2017(30)  Australia | RHM: 21  no RHM: 21 | *Study type:*  Cost-analysis  *Patient population:*  42 participants with COPD and at least one hospital admission due to an exacerbation over last year  *Analysis:*  Study done alongside a RCT  *Perspective:*  Payer  *Time horizon:*  12 months | *Costs included:*  Equipment and technical support: Not included  Hospital resources: wards, operating theatres (e.g. bronchoscopy), imaging, pathology, pharmacy, medical, allied health consultations and administrative-related costs.  Primary care resources: not included  *Information source:*  Electronic health records and billing hospital data  *Other:*  Costs reported in Australian dollars (AUS) | NA | *Cost of hospital admission per patient:*  RHM: AUS$20,440± AUS$19,436  no RHM: AUS$25,722± AUS$31,882  p=NR |
| Vianello 2016 (31)  Italy | RHM: 230  no RHM: 104 | NR | NR | NR | NR |
| Segrelles 2014(32)  Spain | RHM: 30  no RHM: 30 | NR | NR | NR | NR |
| De San Miguel 2013(33)  Australia | RHM: 40  no RHM: 40 | *Study type:*  Cost-analysis  *Patient population:*  80 participants with COPD receiving long-term oxygen therapy  *Analysis:*  Study done alongside a RCT  *Perspective:*  Payer  *Time horizon:*  6 months | *Costs included:*  Equipment and technical support: equipment costs depreciated over three years, monitoring system, nurse home visit, nurse monitoring labour costs  Hospital resources: visits to specialist, ED visits, length of hospitalization  Primary care resources: visits with GP  *Information source:*  Published literature, Australian Bureau of Statistics health service, direct observations  *Other:*  Costs reported in Australian dollars (AUS) | NA | *Annual cost saved per patient using RHM:*  RHM: AUS$2,931±NR  no RHM: NA  p=NA |
| Jehn 2013(34)  Germany | RHM: 32  no RHM: 30 | NR | NR | NR | NR |
| Jodar-Sanchez 2013(35)  Spain | RHM: 24  no RHM: 21 | NR | NR | NR | NR |
| Pare 2013(36)  Canada | RHM: 60  no RHM: 60 | *Study type:*  Cost minimization analysis  *Patient population:*  120 participants with very serious COPD requiring frequent home visits  *Analysis:*  Study done alongside a RCT  *Perspective:* Payer  *Time horizon:*  24 months (12 months before introduction of intervention and 12 months after introduction of intervention) | *Costs included:*  Equipment and technical support: equipment costs depreciated over three years, installation at patients’ homes, hosting and maintaining regional service, training for nurses  Hospital resources: ER visits, hospitalizations  Primary care resources: home visits by nurses and respiratory therapists  *Information source:*  Administrative database, manufacturer  *Other:*  Costs reported in Canadian dollars (CAD) | NA | *Cost per patient 12 months before introducing RHM:*  RHM: CAD12,657± CAD 12,463  no RHM: CAD13,531±CAD11,695  p=NR  *Cost per patient after introducing RHM:*  Within 6 months using intervention:  RHM: CAD4,141±CAD5,241  no RHM: CAD5,564±12,088  p=NR  6 months after RHM technology withdrawal:  RHM: CAD3,697± CAD7,412  No RHM: CAD4,763± CAD7,470  p=NR |
| Chau 2012(37)  Hong Kong | RHM: 30  no RHM: 23 | NR | NR | NR |  |
| Dinesen 2012(38)  Denmark | RHM: 60  no RHM: 51 | *Study type:*  Cost-analysis  *Patient population:*  111 participants with severe or very severe COPD  *Analysis:*  Study done alongside a RCT  *Perspective:*  Payer  *Time horizon:*  10 months | *Costs included:*  Equipment and technical support: Not included  Hospital resources: hospitalization-related costs  Primary care resources: not included  *Information source:*  Patient reimbursement data  *Other:*  Costs reported in Euros (€) |  | *Cost of admission per patient:*  RHM: €3,461 (€679, €6,243)  no RHM: €4,576 (€2,476, €6,677)  p=NR |
| Lewis 2010(39;44)  UK | RHM: 20  no RHM: 20 | NR | NR | NR | NR |
| Au 2015(40)  USA | RHM: 619  no RHM: 619 | NR | NR | NR | NR |
| Davis 2015(41)  USA | RHM: 58  no RHM: 174 | NR | NR | NR | NR |
| ***RHM with feedback vs RHM with no feedback*** | | | | | |
| Sink 2018(42)  USA | RHM: 83  no RHM: 85 | NR | NR | NR | NR |
| Franke 2016(43)  Germany | Total: 53 | NR | NR | NR | NR |
| Notes: Tabak 2014a(28) and Tabak 2014b(29) used the same exercise monitoring device and smartphone technology. De San Miguel 2013 (33) and Lewis 2010 (39;44) used the same telemonitoring device. Segrelles 2014 (32) and Jodar-Sanchez 2013 (35) used the same devices to collect vital signs measures and modem technology to transmit collected measurements.  CI: confidence interval; COPD: chronic obstructive pulmonary disease; ER: emergency room; GP: general practitioner; HF: heart failure; HR: hazard ratio; IHD: Ischemic heart disease; NA: not applicable; NR: not reported; QALY: quality-adjusted life years; RCT: randomized controlled trial; RHM: remote home monitoring; SD: standard deviation | | | | | |

| Table S18. Provider experience | | | |
| --- | --- | --- | --- |
| **Study** | **Sample size** | **Method** | **Findings** |
| ***RHM (smartphones, apps, tablets) vs no RHM*** | | | |
| Park 2020(25)  South Korea | RHM: 23  no RHM: 21 | NR | NR |
| Boer 2019(26)  Netherlands | RHM: 43  no RHM: 44 | NR | NR |
| Walker 2018 (27)  Spain, UK, Slovenia, Estonia, Sweden | RHM: 154  no RHM: 158 | NR | NR |
| Tabak 2014a(28)  Netherlands | RHM: 15  no RHM: 14 | NR | NR |
| Tabak 2014b (29)  Netherlands | RHM: 18  no RHM: 16 | NR | NR |
| ***RHM (dedicated monitoring devices) vs no RHM*** | | | |
| Shany 2017(30)  Australia | RHM: 21  no RHM: 21 | Providers were asked to complete an usability survey. No further details were provided.  *Providers who completed the survey:* two nurses | - Providers became more positive about the usability and value of RHM by the end of follow-up - Providers reported that due to the telehealth units they knew where to prioritize and assist patients - One provider wanted to continue using telehealth for a larger patient group, while another was unsure - Both nurses agreed that they would recommend the telehealth unit to patients and healthcare workers |
| Vianello 2016 (31)  Italy | RHM: 230  no RHM: 104 | NR | NR |
| Segrelles 2014(32)  Spain | RHM: 30  no RHM: 30 | NR | NR |
| De San Miguel 2013(33)  Australia | RHM: 40  no RHM: 40 | NR | NR |
| Jehn 2013(34)  Germany | RHM: 32  no RHM: 30 | NR | NR |
| Jodar-Sanchez 2013(35)  Spain | RHM: 24  no RHM: 21 | Providers were asked to complete a survey on satisfaction with the telehealth program. The survey comprised 7 questions on a 4-point rating scale ranging from strongly disagree to strongly agree. They survey also asked providers to rate their overall level of satisfaction with the program from 1 (very poor) to 10 (very good).  *Providers who completed the survey:* two nurses and two respiratory physicians | **Telehealth program improved patient control and follow-up compared with conventional care- n(%agreed)*:**  RHM: 4(100)  **Compared to conventional care, telehealth program reduced referrals to long specialists- n(%agreed)*:**  RHM: 3(75)  **Compared to conventional care, telehealth program reduced hospital admissions- n(%agreed)*:**  RHM: 4(100)  **Equipment is easy to use for patients- n(%agreed)*:**  RHM: 3(75)  **Alerts generated by monitoring are reliable- n(%agreed)*:**  RHM: 1(25)  **Telehealth program improved patients’ quality of life- n(%agreed)*:**  RHM: 3(75)  **Patients are more satisfied with telehealth program compared to conventional care on its own- n(%agreed)*:**  RHM: 4(100)  **Overall level of satisfaction- mean±SD:**  RHM: 6.7±10.0 |
| Pare 2013(36)  Canada | RHM: 60  no RHM: 60 | NR | NR |
| Chau 2012(37)  Hong Kong | RHM: 30  no RHM: 23 | NR | NR |
| Dinesen 2012(38)  Denmark | RHM: 60  no RHM: 51 | NR | NR |
| Lewis 2010(39;44)  UK | RHM: 20  no RHM: 20 | NR | NR |
| Au 2015(40)  USA | RHM: 619  no RHM: 619 | NR | NR |
| Davis 2015(41)  USA | RHM: 69  no RHM: 174 | NR | NR |
| ***RHM with feedback vs RHM with no feedback*** | | | |
| Sink 2018(42)  USA | RHM: 83  no RHM: 85 | NR | NR |
| Franke 2016(43)  Germany | Total: 53 | NR | NR |
| Notes: Tabak 2014a(28) and Tabak 2014b(29) used the same exercise monitoring device and smartphone technology. De San Miguel 2013 (33) and Lewis 2010 (39;44) used the same telemonitoring device. Segrelles 2014 (32) and Jodar-Sanchez 2013 (35) used the same devices to collect vital signs measures and modem technology to transmit collected measurements.  *Number is based on patients who reported “strongly agreed” and “agreed”.  NR: not reported; RHM: remote home monitoring; SD: standard deviation | | | |

| Table S19. Lung function | | | | | | | | | | | |
| --- | --- | --- | --- | --- | --- | --- | --- | --- | --- | --- | --- |
| **Study** | **Sample size** | **Time** | **FEV_1_ and FVC** | | | **Dyspnea/ breathlessness** | | | **Other symptoms** | | |
|  |  |  | **RHM**  **Mean±SD**  **Median (IQR)** | **Comparison group**  **Mean±SD**  **Median (IQR)** | **Diff. between groups**  **MD(95%CI)** | **RHM**  **Mean±SD** | **Comparison group**  **Mean±SD** | **Diff. between groups**  **MD(95%CI)** | **RHM**  **Mean±SD** | **Comparison group**  **Mean±SD** | **Diff. between groups**  **MD(95%CI)** |
| ***RHM (smartphones, apps, tablets) vs no RHM*** | | | | | | | | | | | |
| Park 2020(25)  South Korea | RHM: 23  no RHM: 21 | Baseline | **FEV_1_% predicted:**  RHM(n=22): 61.0±18.7  **FEV_1_/ FVC ratio:**  RHM(n=22): 62.8±20.8 | **FEV_1_% predicted:**  no RHM(n=20): 69.4±24.0  **FEV_1_/ FVC ratio:**  no RHM(n=20): 65.6±17.9 | **FEV_1_% predicted:**  p=0.21  **FEV_1_/ FVC ratio:**  p=0.64 | **Dyspnea (UCSD-SOB):**  RHM(n=22): 21.2±16.0 | **Dyspnea (UCSD-SOB):**  no RHM(n=20): 19.2±13.8 | **Dyspnea (UCSD-SOB):**  p=0.68 | NR | NR | NR |
|  |  | 6 months  *Diff. within group* | NR | NR | NR | **Dyspnea (UCSD-SOB):**  RHM(n=22): 21.4±17.8  *p>0.05* | **Dyspnea (UCSD-SOB):**  no RHM(n=20): 19.7±14.3  *p>0.05* | **Dyspnea (UCSD-SOB):**  p=0.97 | NR | NR | NR |
| Boer 2019(26)  Netherlands | RHM: 43  no RHM: 44 | Baseline | **FEV_1_% predicted:**  RHM: 53.0±21.5 | **FEV_1_% predicted:**  no RHM: 52.1±19.8 | NR | **Dyspnea emotions (NCSI):**  RHM: 8.8±2.4 | **Dyspnea emotions (NCSI):**  no RHM: 11.6±3.9 | NR | **Subjective impairment (NCSI):**  RHM: 11.8±5.8  **Behavioral impairment(NCSI):**  RHM: 22.1±17.9  **Subjective symptoms(NCSI):**  RHM: 9.9±4.9  **Dyspnea emotions(NCSI):**  RHM: 8.8±2.4  **Fatigue(NCSI):**  RHM: 35.9±11.0 | **Subjective impairment(NCSI):**  no RHM: 14.4±6.6  **Behavioral impairment(NCSI):**  no RHM: 19.1±17.3  **Subjective symptoms(NCSI):**  no RHM: 11.9±4.9  **Dyspnea emotions(NCSI):**  no RHM: 11.6±3.9  **Fatigue(NCSI):**  no RHM: 37.3±10.2 | NR |
|  |  | 12 months | NR | NR | NR | **Dyspnea emotions (NCSI):**  RHM(n=35): 8.7±2.9 | **Dyspnea emotions (NCSI):**  no RHM(n=41): 10.7±4.3 | **Dyspnea emotions (NCSI):**  0.8 (−0.58, 2.3)  p=0.24 | **Subjective impairment(NCSI):**  RHM(n=35): 11.2±4.1  **Behavioral impairment(NCSI):**  RHM(n=35): 20.5±15.6  **Subjective symptoms(NCSI):**  RHM(n=35): 9.2±4.4  **Fatigue(NCSI):**  RHM(n=35): 35.2±9.4 | **Subjective impairment(NCSI):**  no RHM(n=41): 13.2±6.6  **Behavioral impairment(NCSI):**  no RHM(n=41): 20.4±21.8  **Subjective symptoms(NCSI):**  no RHM(n=41): 11.0±4.8  **Fatigue(NCSI):**  no RHM(n=41): 37.7±10.2 | **Subjective impairment(NCSI):**  0.7(−1.4, 2.8)  p=0.50  **Behavioral impairment(NCSI):**  −1.8(−7.2, 3.7)  p=0.52  **Subjective symptoms(NCSI):**  0.3 (−1.4, 2.11)  p=0.69  **Fatigue(NCSI):**  −1.8 (−5.4, 1.8)  p=0.33 |
| Walker 2018 (27)  Spain, UK, Slovenia, Estonia, Sweden | RHM: 154  no RHM: 158 | Baseline | **FEV_1_% predicted:**  RHM: 49.4 (37.1–59.2)  **FEV_1_/ FVC ratio:**  RHM: 50 (40–60) | **FEV_1_% predicted:**  no RHM: 50.4 (38.0–63.9)  **FEV_1_/ FVC ratio:**  no RHM: 50 (40–60) | NR | NR | NR | NR | NR | NR | NR |
| Tabak 2014a(28)  Netherlands | RHM: 15  no RHM: 14 | Baseline | **FEV_1_% predicted:**  RHM(n=12): 50.0 (33.3-61.5) | **FEV_1_% predicted:**  no RHM(n=12): 36.0(26.0- 53.5) | **FEV_1_% predicted:**  p=0.25 | NR | NR | NR | **General fatigue (MFI):**  RHM: 12.3±1.4  **Physical fatigue (MFI):**  RHM: 12.2±3.2  **Reduced activity (MFI):**  RHM: 8.3±1.2  **Reduced motivation (MFI):**  RHM: 9.3±1.1  **Mental fatigue (MFI):**  RHM: 8.5±1.1 | **General fatigue (MFI):**  no RHM: 15.0±1.4  **Physical fatigue (MFI):**  no RHM: 16.5±3.2  **Reduced activity (MFI):**  no RHM: 12.5±1.3  **Reduced motivation (MFI):**  no RHM: 9.4±1.2  **Mental fatigue (MFI):**  no RHM: 7.0±1.2 | NR |
|  |  | 1 month | NR | NR | NR | NR | NR | NR | **General fatigue (MFI):**  RHM(n=11): 11.0±1.1  **Physical fatigue (MFI):**  RHM(n=11): 11.9±3.2  **Reduced activity (MFI):**  RHM(n=11): 9.3±0.9  **Reduced motivation (MFI):**  RHM(n=11): 7.4 ±0.9  **Mental fatigue (MFI):**  RHM(n=11): 9.9±1.4 | **General fatigue (MFI):**  no RHM(n=9): 13.6±1.3  **Physical fatigue (MFI):**  no RHM(n=9): 14.4±3.2  **Reduced activity (MFI):**  no RHM(n=9): 11.7±1.1  **Reduced motivation (MFI):**  no RHM(n=9): 8.2±1.0  **Mental fatigue (MFI):**  no RHM(n=9): 8.0±1.7 | NR |
|  |  | 3 months | NR | NR | NR | NR | NR | NR | **General fatigue (MFI):**  RHM(n=11): 10.4±1.3  **Physical fatigue (MFI):**  RHM(n=11): 11.0±3.3  **Reduced activity (MFI):**  RHM(n=11): 8.2±1.2  **Reduced motivation (MFI):**  RHM(n=11): 7.5±1.2  **Mental fatigue (MFI):**  RHM(n=11): 6.6±0.9 | **General fatigue (MFI):**  no RHM(n=9): 13.8±1.5  **Physical fatigue (MFI):**  no RHM(n=9): 14.4±3.3  **Reduced activity (MFI):**  no RHM(n=9): 10.5±1.3  **Reduced motivation (MFI):**  no RHM(n=9): 8.8±1.3  **Mental fatigue (MFI):**  no RHM(n=9): 7.3±1.1 | NR |
| Tabak 2014b (29)  Netherlands | RHM: 18  no RHM: 16 | Baseline | **FEV_1_% predicted:**  RHM(n=14): 48.7±16.7 | **FEV_1_% predicted:**  no RHM: 56.4±10.6 | **FEV_1_% predicted:**  p>0.05 | **MRC**  RHM(n=14): 2.0±0.9 | **MRC**  no RHM(n=15): 2.3±1.4 | NR | **General fatigue (MFI):**  RHM(n=14): 12.5±4.0  **Physical fatigue (MFI):**  RHM(n=14): 13.1±3.7  **Reduced activity (MFI):**  RHM(n=14): 9.9±3.7  **Reduced motivation (MFI):**  RHM(n=14): 9.0±3.8  **Mental fatigue (MFI):**  RHM(n=14): 6.8±3.2 | **General fatigue (MFI):**  no RHM(n=15): 11.6±4.4  **Physical fatigue (MFI):**  no RHM(n=15): 11.6±3.5  **Reduced activity (MFI):**  no RHM(n=15): 9.9±4.0  **Reduced motivation (MFI):**  no RHM(n=15): 9.3±3.4  **Mental fatigue (MFI):**  no RHM(n=15): 6.6±3.8 | NR |
|  |  | 1 month  *Diff within group MD±SD* | NR | NR | NR | **MRC**  RHM(n=14): NR  *-0.3±0.7*  *p>0.05* | **MRC**  no RHM(n=15): NR  *-0.2±0.9*  *p>0.05* | NR | **General fatigue (MFI):**  RHM(n=14): NR  *–0.9±2.7*  *p>0.05*  **Physical fatigue (MFI):**  RHM(n=14): NR  *–1.1±3.8*  *p>0.05*  **Reduced activity (MFI):**  RHM(n=14): NR  *–1.4±2.7*  *p>0.05*  **Reduced motivation (MFI):**  RHM(n=14): NR  *–0.6±2.2*  *p=0.07*  **Mental fatigue (MFI):**  RHM(n=14): NR  *–0.5±3.5*  *p>0.05* | **General fatigue (MFI):**  no RHM(n=15): NR  *–0.3±2.6*  *p>0.05*  **Physical fatigue (MFI):**  no RHM(n=15): NR  *–0.4±2.7*  *p>0.05*  **Reduced activity (MFI):**  no RHM(n=15): NR  *–0.2±3.6*  *p>0.05*  **Reduced motivation (MFI):**  no RHM(n=15): NR  *–1.1±1.6*  *p=0.02*  **Mental fatigue (MFI):**  no RHM(n=15): NR  *–0.6±3.7*  *p>0.05* | NR |
| ***RHM (dedicated monitoring devices) vs no RHM*** | | | | | | | | | | | |
| Shany 2017(30)  Australia | RHM: 21  no RHM: 21 | Baseline | **FEV_1_% predicted:**  RHM: 32.1+16.0 | **FEV_1_% predicted:**  no RHM: 39.7+13.2 | **FEV_1_% predicted:**  p>0.05 | NR | NR | NR | NR | NR | NR |
| Vianello 2016 (31)  Italy | RHM: 230  no RHM: 104 | Baseline | **FEV_1_% predicted:**  RHM: 41.9±8.6 | **FEV_1_% predicted:**  no RHM: 41.9±8.3 | **FEV_1_% predicted:**  p=0.56 | NR | NR | NR | NR | NR | NR |
| Segrelles 2014(32)  Spain | RHM: 30  no RHM: 30 | Baseline | **FEV_1_% predicted:**  RHM(n=29): 38.3±11.9 | **FEV_1_% predicted:**  no RHM: 37.1±10.8 | **FEV_1_% predicted:**  p=0.52 | NR | NR | NR | NR | NR | NR |
| De San Miguel 2013(33)  Australia | RHM: 40  no RHM: 40 | NR | NR | NR | NR | NR | NR | NR | NR | NR | NR |
| Jehn 2013(34)  Germany | RHM: 32  no RHM: 30 | Baseline | **FEV_1_% predicted:**  RHM: 50.2±15.0 | **FEV_1_% predicted:**  no RHM: 52.6±17.5 | p=0.73 | NR | NR | NR | NR | NR | NR |
|  |  | 9 months  *Diff. within group* | **FEV_1_% predicted:**  RHM(n=27): 52.7±16.7  *2.5±5.2*  *p=0.10* | **FEV_1_% predicted:**  no RHM(n=25): 52.6±19.8  *-0.7±9.2*  *p=0.99* | NR | NR | NR | NR | NR | NR | NR |
| Jodar-Sanchez 2013(35)  Spain | RHM: 24  no RHM: 21 | Baseline | **FEV_1_% predicted:**  RHM: 38±10 | **FEV_1_% predicted:**  no RHM: 37±13 | **FEV_1_% predicted:**  p=0.33 | NR | NR | NR | NR | NR | NR |
| Pare 2013(36)  Canada | RHM: 60  no RHM: 60 | Baseline | **FEV_1_% predicted:**  RHM:28.6±NR | **FEV_1_% predicted:**  no RHM: 28.5±NR | **FEV_1_% predicted:**  p=0.97 | NR | NR | NR | NR | NR | NR |
| Chau 2012(37)  Hong Kong | RHM: 30  no RHM: 23 | Baseline | **FEV_1_% predicted:**  RHM(n=22): 38.8±12.9  **FEV_1_/ FVC ratio:**  RHM(n=22): 42.1±10.8 | **FEV_1_% predicted:**  no RHM(n=18): 37.7±16.5  **FEV_1_/ FVC ratio:**  no RHM(n=18): 41.0±12.1 | **FEV_1_% predicted:**  p>0.05  **FEV_1_/ FVC ratio:**  p>0.05 | NR | NR | NR | NR | NR | NR |
|  |  | Mean  RHM: 65.2 days  no RHM: 68.4 days | **FEV_1_% predicted:**  RHM(n=22): 33.6±14.6  **FEV_1_/ FVC ratio:**  RHM(n=22): 44.1±13.8 | **FEV_1_% predicted:**  no RHM(n=18): 39.8±15.4  **FEV_1_/ FVC ratio:**  no RHM(n=18): 50.8±16.6 | **FEV_1_% predicted:**  NR  p=0.48  **FEV_1_/ FVC ratio:**  NR  p=0.24 | NR | NR | NR | NR | NR | NR |
| Dinesen 2012(38)  Denmark | RHM: 60  no RHM: 51 | NR | NR | NR | NR | NR | NR | NR | NR | NR | NR |
| Lewis 2010(39;44)  UK | RHM: 20  no RHM: 20 | Baseline | **FEV_1_% predicted:**  RHM: 38±16 | **FEV_1_% predicted:**  no RHM: 40±15 | **FEV_1_% predicted:**  p=0.73 | NR | NR | NR | NR | NR | NR |
| Au 2015(40)  USA | RHM: 619  no RHM: 619 | NR | NR | NR | NR | NR | NR | NR | NR | NR | NR |
| Davis 2015(41)  USA | RHM: 58  no RHM: 174 | NR | NR | NR | NR | NR | NR | NR | NR | NR | NR |
| ***RHM with feedback vs RHM with no feedback*** | | | | | | | | | | | |
| Sink 2018(42)  USA | RHM: 83  no RHM: 85 | Baseline | **FEV_1_% predicted:**  RHM(n=63):  65±3  **FEV_1_/ FVC ratio:**  RHM(n=63):  64±2 | **FEV_1_% predicted:**  no RHM(n=72):  63±2  **FEV_1_/ FVC ratio:**  no RHM(n=72):  61±2 | **FEV_1_% predicted:**  p=0.69  **FEV_1_/ FVC ratio:**  p=0.17 | NR | NR | NR | NR | NR | NR |
| Franke 2016(43)  Germany | Total: 53 | Baseline | **FEV_1_% predicted:**  RHM and no RHM(n=44): 47.5±15.8 | | **FEV_1_% predicted:**  p=NA |  | NR | NR | NR | NR | NR |
| Notes: 2014a(28) and Tabak 2014b(29) used the same exercise monitoring device and smartphone technology. De San Miguel 2013 (33) and Lewis 2010 (39;44) used the same telemonitoring device. Segrelles 2014 (32) and Jodar-Sanchez 2013 (35) used the same devices to collect vital signs measures and modem technology to transmit collected measurements.  CI: confidence interval; FEV_1_: forced expiratory volume in one second; FVC: forced vital capacity; IQR: interquartile range; MCR: Medical Research Council; MD: mean difference; MFI: Multidimensional Fatigue Inventory; NA: not applicable; NCSI: Nijmegen Clinical Screening Instrument; NR: not reported; RHM: remote home monitoring; SD: standard deviation | | | | | | | | | | | |
